# Unexpected Synthesis of a Furoxan Derivative from 3-Acetyl-2,4,6-Trimethylpyridine: Structural Characterization and Biological Evaluation

**DOI:** 10.3390/molecules31162842

**Published:** 2026-08-14

**Authors:** Aida S. Rakhimzhanova, Irina A. Pustolaikina, Alfiya F. Kurmanova, Ruslan A. Muzaparov, Tatyana V. Rybalova, Zarina T. Shulgau, Alena L. Stalinskaya, Ivan V. Kulakov

**Affiliations:** 1 Department of Physical and Analytical Chemistry, Buketov Karaganda National Research University, Karaganda 100024, Kazakhstan; aida_ekb@mail.ru (A.S.R.); alfiya_kurmanova@mail.ru (A.F.K.); mega.muzaparov@mail.ru (R.A.M.); i.v.kulakov@utmn.ru (I.V.K.); 2 N. N. Vorozhtsov Novosibirsk Institute of Organic Chemistry of the Siberian Branch of Russian Academy of Sciences, Novosibirsk 630090, Russia; rybalova@nioch.nsc.ru; 3 Department of Science Development, Astana Medical University, Astana 010000, Kazakhstan; shulgau.z@amu.kz; 4 Higher School of Natural Science, University of Tyumen, Tyumen 625003, Russia; a.l.stalinskaya@utmn.ru

**Keywords:** acetylpyridines, furoxans, cyclization, pyridine derivatives, nitration, 3-acetyl-2,4,6-trimethylpyridine, pseudo-multicomponent reactions, 1,2,5-oxadiazole 2-oxides, 1,2,5-oxadiazoles, crystal structure, DFT calculations, perturbation theory, molecular docking, MMP-9, hemorheological activity

## Abstract

Herein, we report an unexpected pseudo-multicomponent transformation discovered during attempts to selectively nitrate the pyridine core of 3-acetyl-2,4,6-trimethylpyridine (**3**). Despite employing standard nitration conditions, including KNO_3_–H_2_SO_4_ and HNO_3_–H_2_SO_4_ mixtures, electrophilic substitution of the aromatic ring did not occur. Instead, the reaction sequence promoted an in situ nitrozation, dehydration to nitrile oxide intermediates, and subsequent [3+2]-cycloaddition involving two substrate molecules. This process yielded a novel, highly functionalized furoxan derivative, precisely identified as 3,4-bis(2,4,6-trimethylnicotinoyl)-1,2,5-oxadiazole 2-oxide (**5**). The molecular architecture of compound **5** was established by ^1^H and ^13^C NMR spectroscopy, mass spectrometry, elemental analysis, and single-crystal X-ray diffraction (XRD) analysis. To elucidate the stereochemical and electronic features governing compound **5**, DFT calculations were performed at the ωB97X-D/6-311++G(d,p) level of theory. The experimental crystallographic disorder of the N-oxide oxygen atom was computationally rationalized by the thermodynamic near-degeneracy (ΔG < 0.63 kcal/mol) of two orientational isomers (**5a** and **5b**). Furthermore, frontier molecular orbital analysis within the framework of perturbation theory accounted for the head-to-tail regioselectivity during cyclization, while wide energy gaps (ΔE = 8.13–8.27 eV) and high chemical hardness (η = 4.07–4.14 eV) underscored the kinetic stability of the heterocycle. Phenotypic and target-specific in silico profiling using PASS Online identified Matrix Metalloproteinase-9 (MMP-9) as a relevant target for potential hemorheological and cardioprotective applications. Validated molecular docking simulations across three human MMP-9 crystallographic domains (PDB: **8K5Y, 6ESM, 4XCT**) demonstrated competitive binding affinities and balanced Ligand Efficiency metrics (LE = 0.26–0.29 kcal/mol/heavy atom), anchoring compound **5** within the catalytic pocket via conventional hydrogen bonds and *π*-mediated interactions. Finally, in vitro evaluations using a blood hyperviscosity model confirmed significant hemorheological efficacy, as compound **5** effectively prevented the rise in blood viscosity, outperforming the reference drug pentoxifylline. The convergence of computational insights and experimental functional activity establishes this novel bis(nicotinoyl)furoxan framework as a promising candidate for further hemorheological and cardioprotective applications.

## 1. Introduction

Pyridine derivatives are essential components of vital vitamins such as B5 and B6 and play a crucial role in biological processes [1,2]. These compounds are widely utilized in medicine as pharmaceuticals with a broad spectrum of therapeutic activities, including antibacterial, antitubercular, antidepressant, antihistamine, analgesic, psychotropic, and nootropic effects [3,4]. Furthermore, they find extensive application in agriculture as highly effective fungicides, herbicides, and plant growth regulators [5].

**Figure 1:**
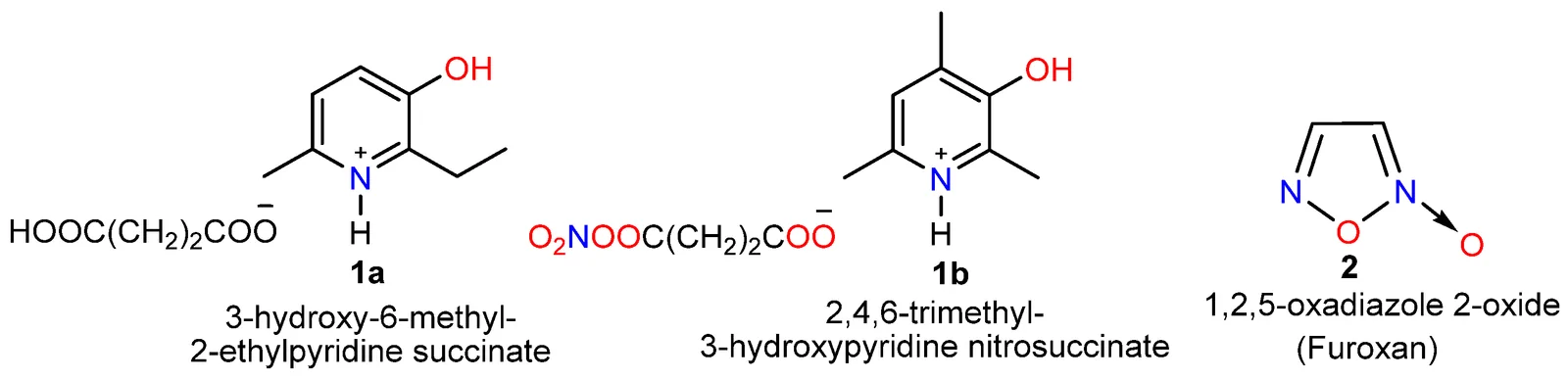
Chemical structures of Mexidol succinate (**1a**), its structural analogue 2,4,6-trimethylpyridin-3-ol nitrosuccinate (**1b**), and the 1,2,5-oxadiazole 2-oxide (furoxan) core (**2**).

The structural characteristics of active substances, particularly the spatial maintenance of pharmacophores in their active conformations, play a critical role in determining the pharmacological effect [6]. The presence of hydroxyl and alkyl groups, which possess varying electron-donating properties, contributes to the antioxidant activity of pyridine derivatives [7]. For instance, Mexidol (2-ethyl-6-methylpyridin-3-ol succinate, Figure 1, **1a**) exhibits pronounced antioxidant, membrane-protective, and metabolic modulating properties [6]. In 2005, a closely related analogue of Mexidol, 2,4,6-trimethylpyridin-3-ol nitrosuccinate (Figure 1, **1b**), was synthesized and patented [8]. This collidine derivative (**1b**) represents a promising anti-ischemic agent with a vasodilatory effect, capable of exerting a pronounced protective action against secondary tissue necrosis [9].

**Figure 2:**
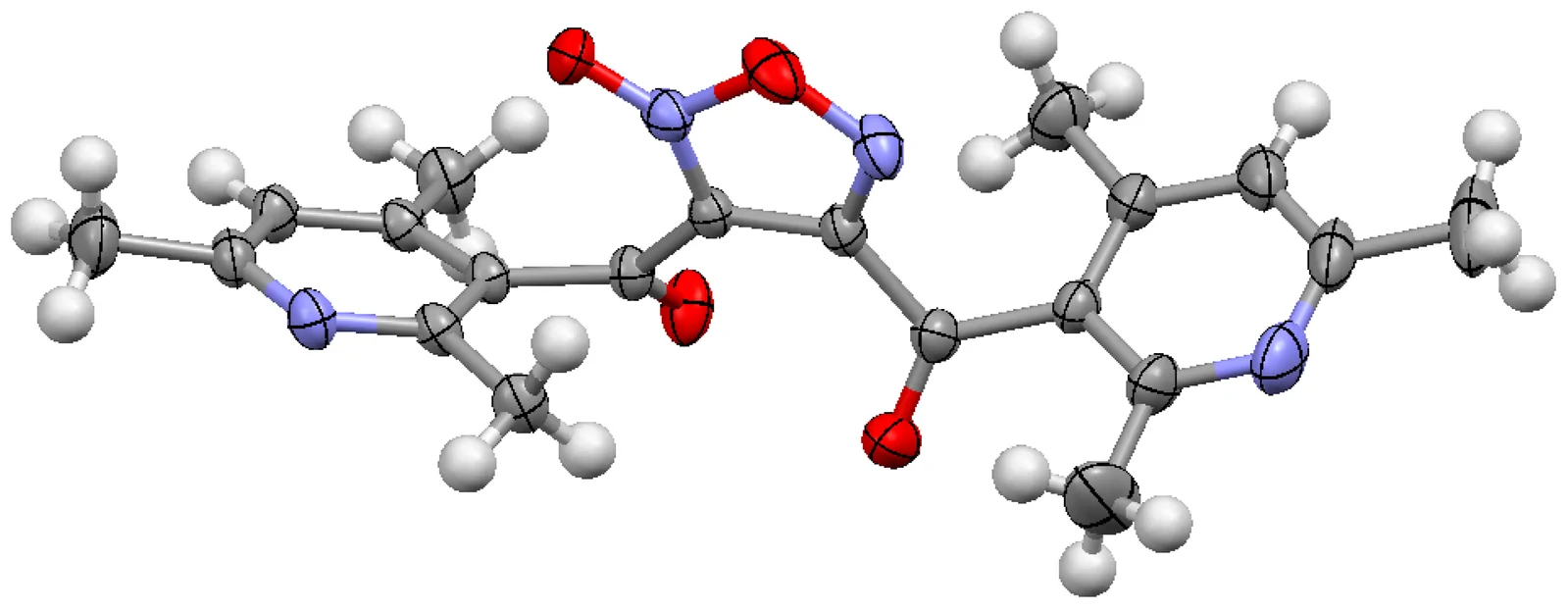
Spatial structure of 3,4-bis(2,4,6-trimethylnicotinoyl)-1,2,5-oxadiazole-2-*N*-oxide (**5**).

On the other hand, furoxans (1,2,5-oxadiazole 2-oxides, Figure 1, **2**) represent a remarkably important class of heterocyclic compounds widely recognized for their rich medicinal chemistry applications [10,11]. The unique pharmacophoric feature of the furoxan ring stems from its ability to act as a nitric oxide (NO) donor under physiological conditions upon reaction with endogenous thiols [12]. Owing to controlled NO release, furoxan derivatives display a broad spectrum of biological activities, including vasodilatory, antiplatelet, antitumor, hybrid cardiovascular, and antibacterial effects [13].

From a synthetic perspective, furoxans are traditionally accessed via the dimerization of in situ generated nitrile oxides, oxidation of dioximes, or nitration of alkenes and activated carbonyl compounds [14]. In recent years, pseudo-multicomponent reactions (pseudo-MCRs)—wherein three or more molecules assemble in a single operational step to construct complex scaffolds—have emerged as powerful tools in heterocyclic chemistry due to their high step-economy and operational simplicity [15]. The unexpected formation of furoxan architectures via pseudo-MCR pathways represents a highly desirable yet challenging synthetic transformation [16]. In this context, acetyl derivatives of pyridine serve as versatile building blocks for the construction of structural analogues with tailored properties. The aim of this work was to investigate the chemical transformations of 3-acetyl-2,4,6-trimethylpyridine (**3**). Unexpectedly, the nitration protocol led to a pseudo-multicomponent coupling reaction yielding a novel furoxan derivative. Herein, we report the synthesis, structural characterization and biological evaluation of 3,4-bis(2,4,6-trimethylnicotinoyl)-1,2,5-oxadiazole 2-oxide (**5**).

## 2. Results and Discussion

### 2.1. Description of Syntheses and Characterization of New 3,4-bis(2,4,6-Trimethylnicotinoyl)-1,2,5-oxadiazole-2-N-oxide (***5***)

As early as 1947, a remarkably simple, one-step laboratory method for synthesizing 3-acetyl-2,4,6-trimethylpyridine (**3**), a functional acetyl derivative of collidine, was reported [17]. However, since this initial synthesis was published, no further data regarding its potential chemical modifications have appeared in the literature.

To introduce electron-withdrawing substituents into the 3-acetylcollidine core, nitration appears to be a highly reasonable approach. The three electron-donating methyl groups not only facilitate electrophilic substitution of the pyridine ring but also direct the nitro group regioselectively to the vacant 5-position.

**Scheme 1:**
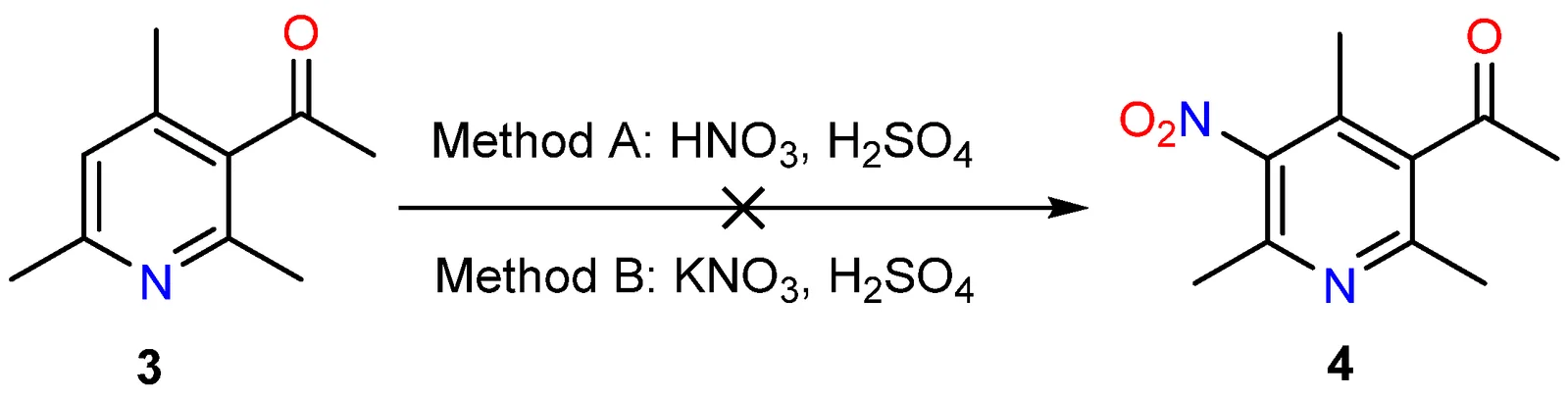
Nitration reaction of 3-acetyl-2,4,6-trimethylpyridine **3**.

For this purpose, a series of nitration reactions of 3-acetyl-2,4,6-trimethylpyridine (**3**) were carried out under different conditions: using a nitrating mixture of concentrated HNO_3_–concentrated H_2_SO_4_ (Method A) and using KNO_3_ in the presence of concentrated H_2_SO_4_ (Method B) (Scheme 1). The selection of these classic nitrating systems, commonly employed for lutidines and picolines [18,19,20,21], was dictated by the complex electronic nature of substrate **3**. While the three electron-donating methyl groups activate the pyridine core toward electrophilic attack, the presence of the strongly electron-withdrawing acetyl moiety significantly deactivates the aromatic nucleus. In a strongly acidic medium, complete protonation of the pyridine nitrogen prevents undesirable N-oxidation while simultaneously promoting the efficient in situ generation of active electrophilic species to overcome the deactivating effect of the acetyl group.

In the former case (Method A: a 1:1 mixture of concentrated nitric and sulfuric acids, 0 to 20 °C), reaction monitoring via thin-layer chromatography (TLC) indicated that nitration did not occur under these conditions, with the starting 3-acetyl-2,4,6-trimethylpyridine (**3**) remaining intact in the reaction mixture (Figure S1, Supplementary Materials). Increasing the temperature to 60–90 °C likewise failed to yield the desired nitration product (**4**).

In the latter case (Method B: employing the inorganic nitrating reagent potassium nitrate in concentrated sulfuric acid, 0 to 70 °C), a very interesting result was obtained. Gas chromatography–mass spectrometry (GC-MS) analysis of the processed reaction mixture revealed the formation of a product with a molecular mass of 364 (molecular ion peak [M]^+^ = 364.10 m/z) (Figure S2, Supplementary Materials), rather than the expected value of 208 for the targeted nitropyridine (**4**).

**Scheme 2:**
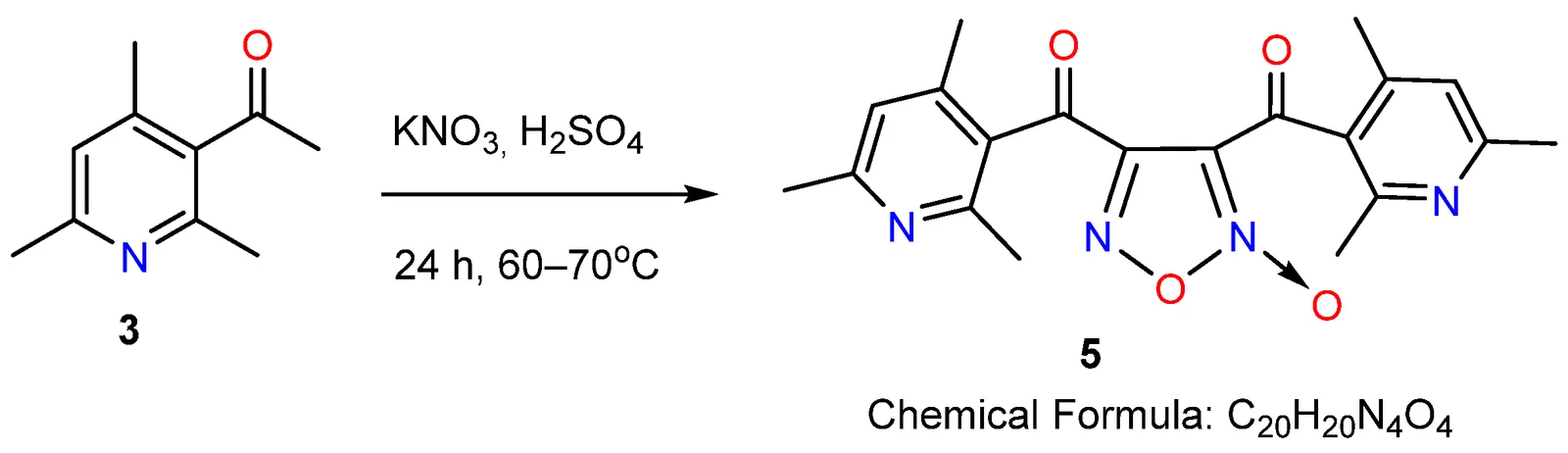
Synthesis of 3,4-bis(2,4,6-trimethylnicotinoyl)-1,2,5-oxadiazol-2-N-oxide (**5**).

The reaction product was isolated in pure form as white crystals with a melting point of 95–97 °C and further characterized by NMR spectroscopy (Figure S3, Supplementary Materials). Comprehensive analysis of the NMR spectra revealed that the compound (**5**) contains two trimethylpyridine rings, featuring non-equivalent protons for all methyl groups. Notably, while the acetyl proton signals were absent, the ^13^C NMR spectrum exhibited resonances for all twenty carbon atoms originating from the two starting pyridine rings, including the carbonyl carbons. These findings indicate that the product (**5**) was formed via the condensation of two pyridine (**3**) molecules at the methylene component of the acetyl groups (Scheme 2).

The structure of the synthesized heterocyclic system, connecting two nicotinoyl fragments, was established by single-crystal X-ray diffraction analysis. Thus, the isolated compound is a diacylfuroxan derivative—3,4-bis(2,4,6-trimethylnicotinoyl)-1,2,5-oxadiazole-2-*N*-oxide (**5**) (Figure 2).

Furthermore, the structure of furoxan **5** was unambiguously confirmed by NMR spectroscopy, mass spectrometry, and elemental analysis. Analysis of the NMR spectra revealed that compound **5** contains two 2,4,6-trimethylpyridine rings featuring magnetic non-equivalence across the protons of all three methyl groups. Distinct singlets are observed for the methyl groups at C-2/C-2′ (δ_H_ = 2.23 and 2.28 ppm), C-6/C-6′ (δ_H_ = 2.42 and 2.44 ppm), and C-4/C-4′ (δ_H_ = 2.49 and 2.50 ppm) of the two pyridine rings, whereas the two aromatic protons (H-5 and H-5′ of the Py rings) resonate as a single overlapping singlet at δ_H_ = 6.92 ppm. The non-equivalent nature of the methyl protons belonging to the two 2,4,6-trimethylnicotinoyl fragments attached to the asymmetric furoxan ring is presumably driven by the spatial anisotropic influence of the furoxan N-oxide moiety. This group exerts a deshielding effect on the nearby C-2′-CH_3_, C-4′-CH_3_, and C-6′-CH_3_ protons of one pyridine ring, downfield-shifting their resonances compared to the methyl protons of the second pyridine core, which lies beyond the direct influence of the electron-withdrawing N-oxide group (Figures S2 and S3). This magnetic non-equivalence is further corroborated by ^13^C NMR spectroscopy, which exhibits five distinct resonances for the methyl carbons: four individual signals (δ_C_ = 19.2, 19.6, 22.9, and 23.6 ppm) and one overlapping signal corresponding to two carbons (δ_C_ = 24.6 ppm). Additionally, two characteristic carbonyl carbon resonances are recorded at δ_C_ = 183.7 and 187.1 ppm, corresponding to the acyl substituents at the C-3 and C-4 positions of the central 1,2,5-oxadiazole 2-oxide ring. The mass spectrum displays the expected peak at M/Z = 364.07, corresponding to the characteristic loss of an oxygen atom [M-O]^+∙^ from the N-oxide core, along with key fragment ions resulting from the cleavage of the carbonyl–furoxan bonds, thereby confirming the proposed 3,4-diacylfuroxan structure.

A literature search on diacylfuroxan derivatives revealed that analogous compounds are typically synthesized via the reaction of aromatic methyl ketones with nitrosating or nitrating agents, such as dilute HNO_3_ in the presence of AcOH, concentrated HNO_3_ containing nitrogen oxides [22], Fe(NO_3_)_3_·9H_2_O with P_2_O_5_ [23], or NaNO_2_ in AcOH [24]. Furoxan derivatives exhibit a broad spectrum of biological properties, including neuroprotective [25], cytotoxic [26], antiparasitic [27,28], antibacterial [29], and antiaggregant activities [30]. This diverse pharmacological profile is primarily attributed to their ability to act as exogenous nitric oxide (NO) donors [31,32,33].

The non-standard pathway observed for this reaction (Scheme 2) is likely driven by the presence of the free acetyl group at the 3-position of pyridine (**3**). We hypothesized that preventing this side reaction would require prior protection of this functional group, for instance, via its reduction to the corresponding hydroxyl group.

**Scheme 3:**
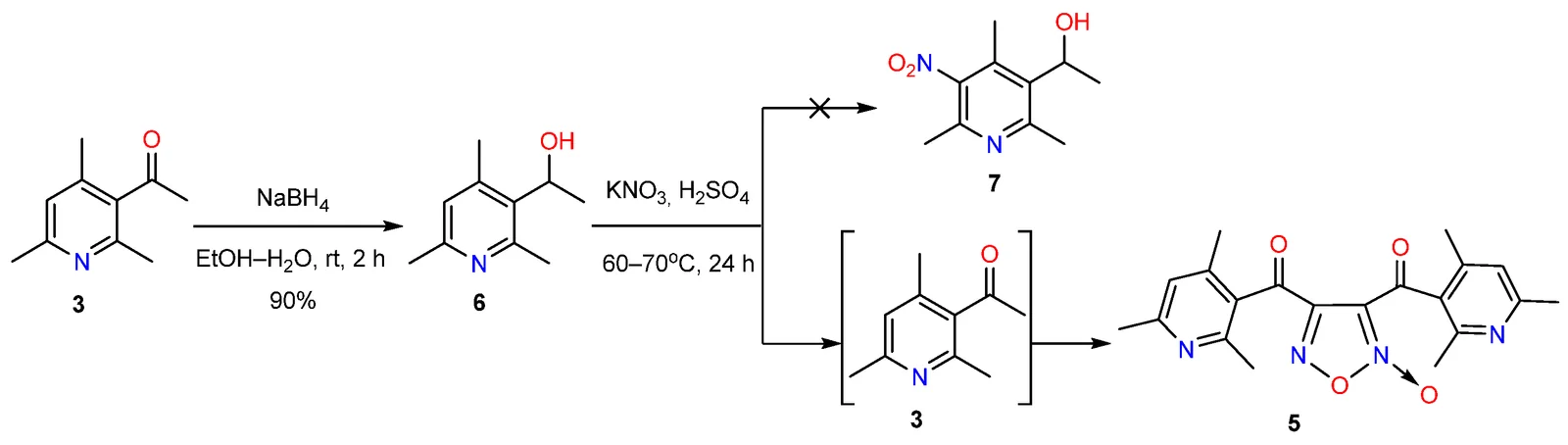
Reduction of 3-acetyl-2,4,6-trimethylpyridine (**3**) to 1-(2,4,6-trimethylpyridin-3-yl)ethan-1-ol (**6**) and its subsequent nitration.

Based on the aforementioned rationale, the reduction of the acetyl group in 3-acetyl-2,4,6-trimethylpyridine (**3**) to the corresponding hydroxyl group was carried out using a 5-fold excess of sodium borohydride in aqueous ethanol (Scheme 3, Table S1) [34].

In the subsequent step, the reduction product (**6**) was subjected to nitration under the previously established conditions utilizing KNO_3_ in the presence of concentrated H_2_SO_4_ (Scheme 3). However, GC-MS analysis of the reaction mixture revealed that under these conditions, in situ oxidation of the hydroxyl group back to the acetyl moiety occurs, followed by intermolecular heterocyclization to afford furoxan derivative (**5**).

**Scheme 4:**
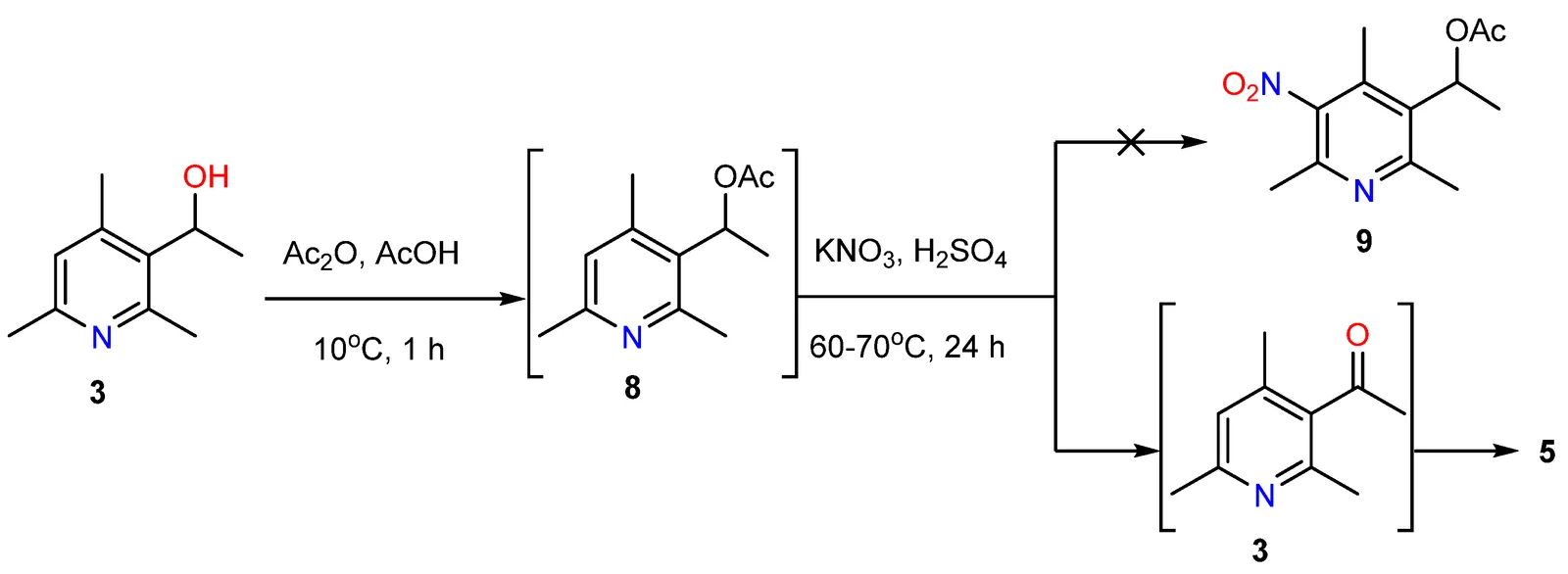
Acylation of the hydroxyl group of pyridine **6** with acetic anhydride.

Therefore, to enhance the stability of the hydroxyl group in pyridine (**6**), acetylation was subsequently carried out using acetic anhydride in the presence of acetic acid (Scheme 4). The formation of acetylated derivative (**8**) was confirmed via GC-MS analysis; following this, the intermediate was subjected to nitration under classical conditions without prior isolation from the reaction mixture.

Chromatographic analysis of the reaction mixture revealed analogous results: instead of the expected nitration product (**9**), deprotection occurred, followed by oxidation of the hydroxyl group to the carbonyl moiety, and subsequent coupling of two pyridine molecules to yield furoxan (**5**).

It is worth noting that when the inorganic nitrating reagent, potassium nitrate, was replaced with concentrated nitric acid in the two aforementioned reactions, the targeted nitration product (**9**) was likewise not observed, mirroring the outcome obtained with the unprotected acetyl group of pyridine (**3**) (Scheme 1, Method A).

Thus, all attempts to selectively nitrate the pyridine core in the presence of an acetyl group or its protected derivatives invariably led to a cascade process involving oxidation, intermolecular condensation, and subsequent formation of the furoxan core. This unusual chemical behavior is presumably driven by the high thermodynamic stability of the resulting 1,2,5-oxadiazole 2-oxide system, combined with the specific electronic effects of the collidine scaffold that promote unexpected in situ oxidative pathways under nitrating conditions. Consequently, the discovered transformation represents a novel, unexpected, yet efficient one-pot synthetic route to highly functionalized bis(nicotinoyl)furoxan derivatives, expanding the synthetic utility of sterically hindered acetylpyridines.

**Scheme 5:**
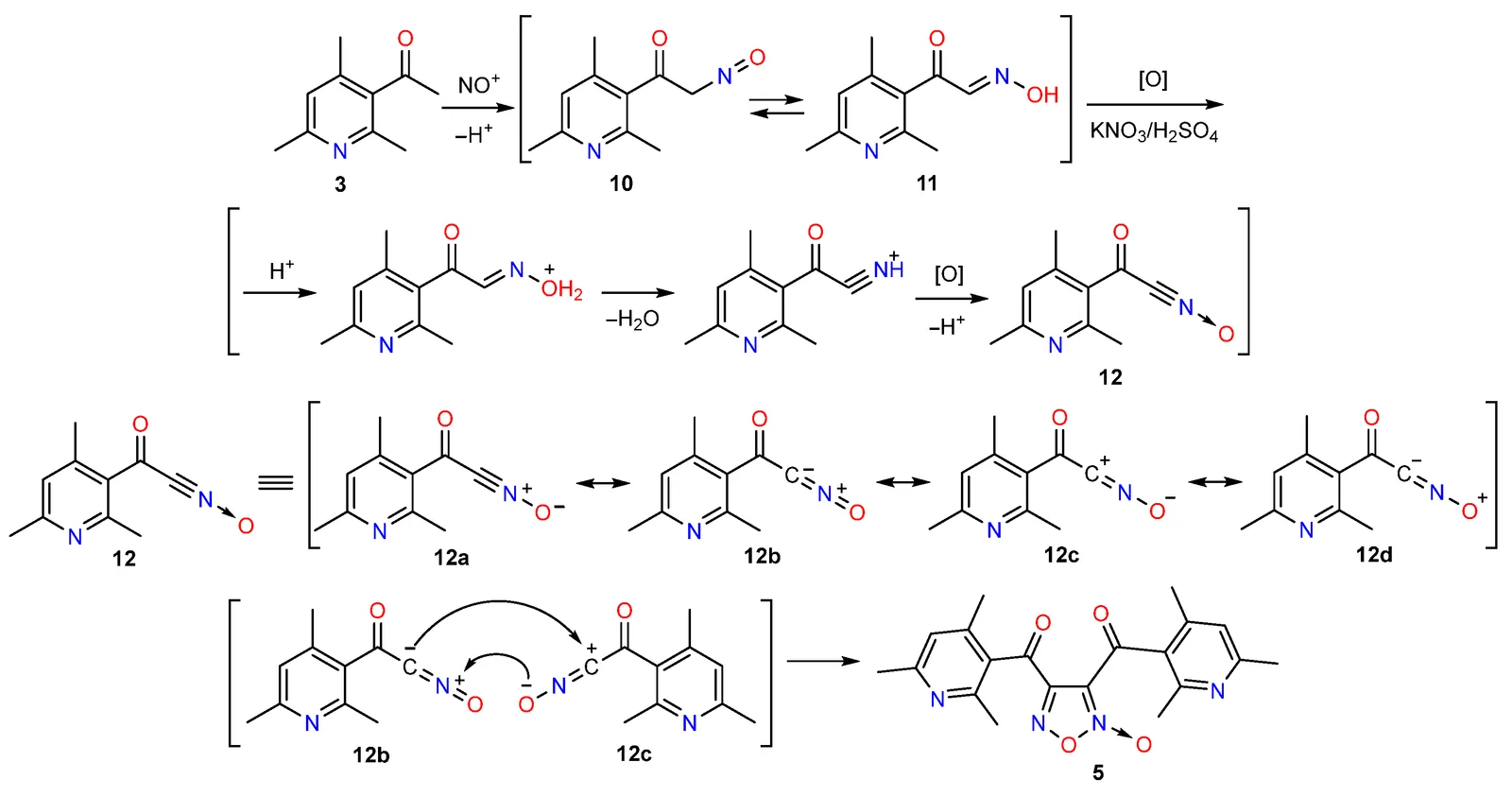
Proposed mechanism of the pseudo-three-component reaction.

Based on the literature data [35,36], the formation of 3,4-disubstituted furoxan **5** from ketone **3** under the optimized nitration conditions (${\mathrm{KNO}}_{3}/H_{2}{\mathrm{SO}}_{4}$ mixture) can be rationalized as a pseudo-three-component reaction, wherein the same starting substrate sequentially participates in multiple assembly stages of the target heterocycle. The proposed mechanism proceeds via electrophilic nitrosation, keto–nitroso/oxime tautomerization, oxidative generation of an in situ nitrile oxide intermediate, and its subsequent dimerization/cyclization (Scheme 5) [37,38,39,40,41].

In the initial step, two equivalents of pyridinyl ketone **3** undergo electrophilic nitrozation of the aceto group driven by the nitrosyl cation NO^+^, generated in situ from nitrate ions in a strongly acidic medium, rather than direct nitration of the aromatic core. As a result, two molecules of α-nitrosoketone (**10**) are formed, which quickly tautomerize into the corresponding α-oximinoketones (**11**). Due to the strong oxidizing environment (KNO_3_/H_2_SO_4_), which generates NO^+^ that acts as an oxidizing agent, oximes (**11**) undergo oxidative dehydrogenation to form highly reactive 1,3-dipoles, namely aroylformonitrile oxides (**12**). Nitrile oxides (**12**) exist as resonance hybrids of canonical forms (**12a**–**d**). Due to this, the nitrile oxides exhibit ambiphilic reactivity, allowing it to act either as a nucleophile (via forms **12b** or **12d**, bearing a negative charge on carbon) or as an electrophile (via form **12c**, bearing a positive charge on carbon).

The key step of the process is the spontaneous dimerization of nitrile oxides (**12**), which proceeds via a concerted [3+2]-cycloaddition. In the transition state, one molecule reacts as a nucleophile (form **12b**), the other as an electrophile (form **12c**). This cross-linking interaction forms a C–C bond between two identical molecules. Ring closure yields the furoxan core in compound (**5**) with acyl substituents at positions C-3 and C-4.

### 2.2. Molecular Structure of 3,4-bis(2,4,6-Trimethylnicotinoyl)-1,2,5-oxadiazole-2-N-oxide ***5***: XRD and DFT Study

To gain deeper insights into the spatial configuration and electronic properties of the newly synthesized furoxan derivative (**5**), a comprehensive study combining single-crystal X-ray diffraction (XRD) analysis with density functional theory (DFT) calculations was performed.

The single-crystal X-ray diffraction analysis unambiguously established that compound (**5**) is a coupling product of two 3-acetylpyridine rings linked by a central 1,2,5-oxadiazole five-membered heterocycle, precisely identified as 3,4-bis(2,4,6-trimethylnicotinoyl)-1,2,5-oxadiazole 2-oxide (Figure 2). The exocyclic oxygen atom of the central furoxan core exhibits orientational disorder with a statistical occupancy ratio of approximately 4:1 (~80:20%). This phenomenon arises from the co-crystallization of two 180°-rotated configurations, in which the central ring adopts two distinct spatial orientations while the flexible side-chain substituents adjust to satisfy crystal packing requirements.

Following the determination of the spatial structure of compound (**5**), a search for similar architectures was performed using the SciFinder database [42]. This revealed closely related structures previously reported in patent [43], which describes furoxan derivatives characterized by prominent biological properties. Specifically, these compounds act as nitric oxide donors that activate the soluble form of guanylate cyclase, inhibit platelet aggregation, and exhibit significant spasmolytic, vasodilating, and hypotensive effects.

**Figure 3:**
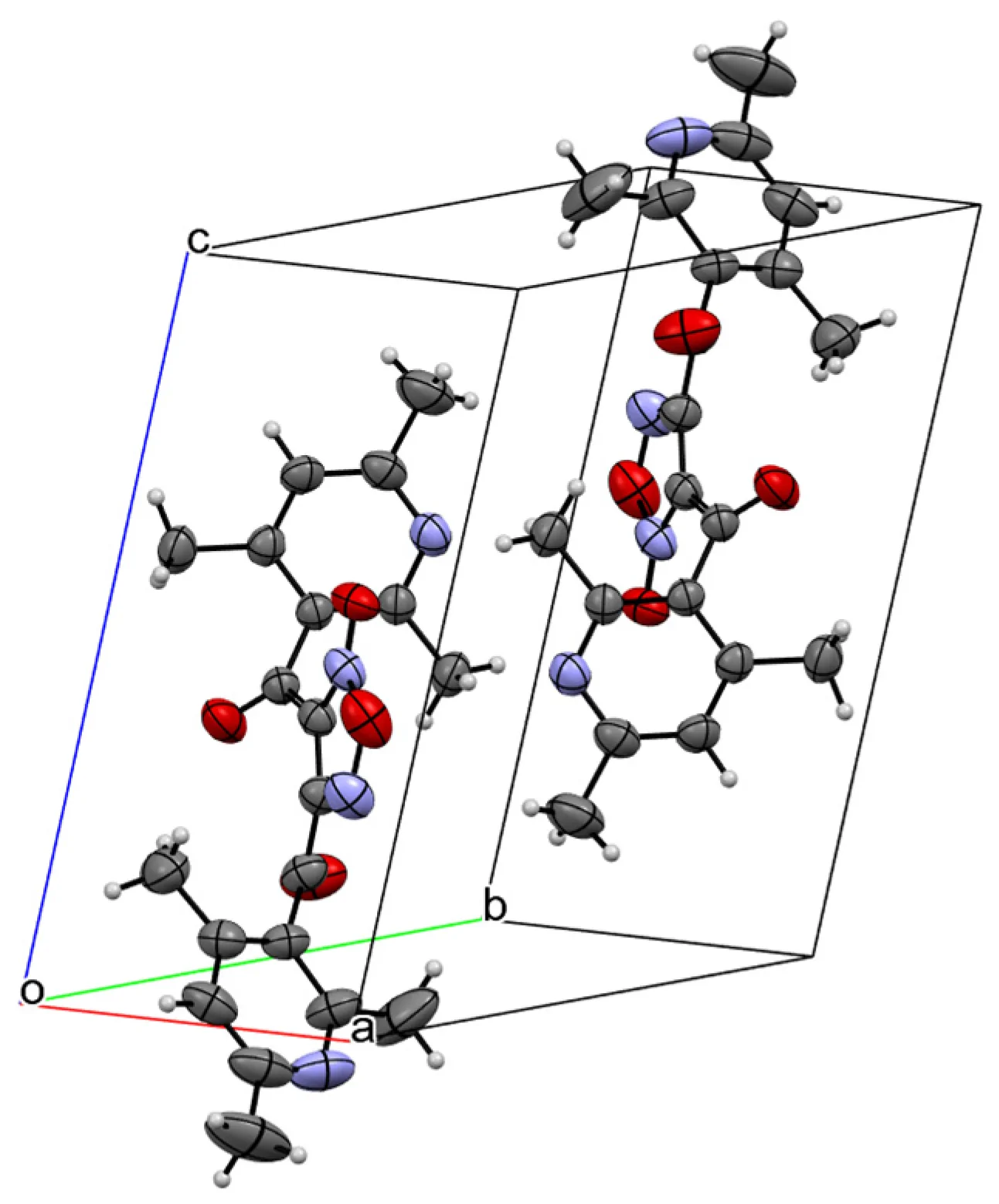
Crystal packing of 3,4-bis(2,4,6-trimethylnicotinoyl)-1,2,5-oxadiazole 2-oxide (**5**) within the triclinic unit cell. Displacement ellipsoids are drawn at the 50% probability level; hydrogen atoms are shown as spheres of arbitrary radii. The crystallographic axes a, b, and c are color-coded in red, green, and blue, respectively.

To better understand the spatial organization of compound (**5**) architecture in the solid state, the detailed arrangement of the molecules within the crystal lattice was examined, as illustrated in the unit cell packing diagram (Figure 3).

Single-crystal X-ray diffraction analysis established the molecular structure and packing parameters of compound (**5**). Single crystals of (**5**) belong to the triclinic crystal system and pack in the centrosymmetric space group $P\bar{1}$ with the following unit cell parameters: a = 8.4541(4) Å, b = 9.2837(4) Å, c = 13.2823(6) Å, α = 77.403(2)°, β = 76.916(2)°, γ = 80.939(2)°, and V = 984.53 Å^3^. The asymmetric unit contains a single independent molecule (Z′ = 1), while the total number of molecules per unit cell is Z = 2. A reliable R-factor of 5.75% confirmed the high quality of the structural data.

The crystal packing of 3,4-bis(2,4,6-trimethylnicotinoyl)-1,2,5-oxadiazole 2-oxide (**5**) is primarily stabilized by a dense network of weak intermolecular interactions (Figure 3). Slightly shortened O…H (2.43–2.63 Å), N…H (2.65 Å), and O…C (3.124(3) Å) compared to van der Waals radius sums for O…H (2.68 Å), N…H (2.74 Å) and O…C (3.35(3) Å) [44]. Within the unit cell, the sterically hindered trimethylpyridine fragments adopt a mutually turned conformation relative to the central furoxan ring, minimizing steric hindrances and ensuring efficient spatial arrangement. The displacement ellipsoids plotted at the 50% probability level illustrate the rigidity of the core heteroaromatic systems, alongside the expected higher thermal mobility observed for the peripheral methyl groups.

**Figure 4:**
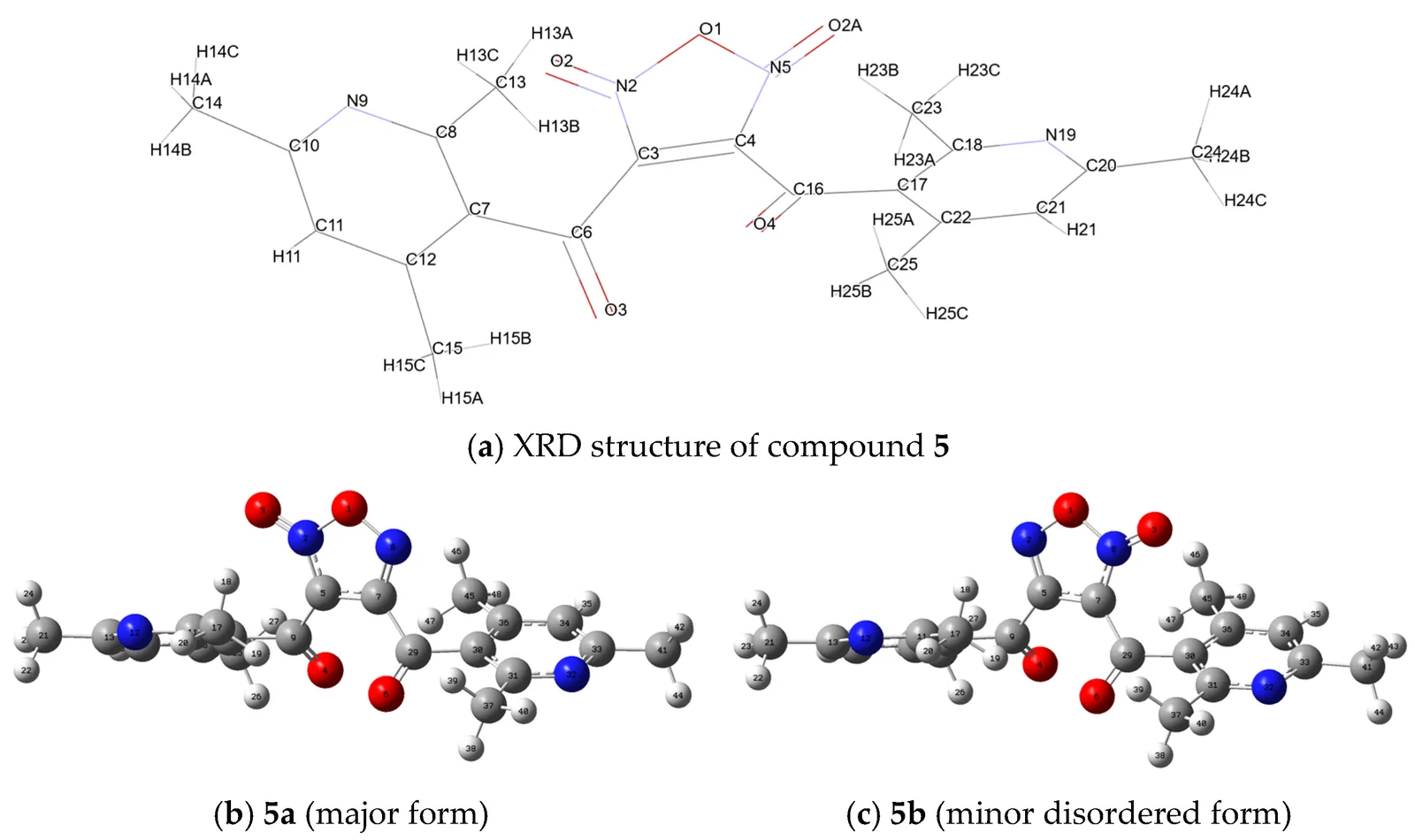
Structure of compound **5**: (**a**) Experimental single-crystal X-ray structure showing the atom-numbering scheme; the apparent presence of exocyclic oxygen atoms on both ring nitrogen atoms is a result of crystallographic orientational disorder, where the central furoxan ring adopts two 180-rotated configurations in the crystal lattice with a major/minor occupancy ratio of approximately 80:20. Optimized geometries at the ωB97X-D/6-311++G(d,p) level of theory for the major (**5a**, (**b**)) and minor (**5b**, (**c**)) orientational isomers.

To complement the experimental X-ray diffraction data and gain deeper insights into the conformational behavior and electronic structure of compound (**5**), quantum chemical calculations were performed using the Gaussian 16 software [45]. Full geometry optimizations followed by vibrational frequency calculations (OPT + FREQ) [46] of compound (**5**), starting from the respective CIF data, were systematically carried out both in the gas phase (vacuum) and in a solvent medium (water) utilizing the Conductor-like Polarizable Continuum Model (CPCM) [47]. For a comprehensive comparative analysis, three density functional theory (DFT) methods were employed: M06-2X/def2-TZVP [48,49], ωB97X-D/6-311++G(d,p) [50,51], and B3LYP-D3/6-311++G(d,p) [52,53,54] (Table S2). Since the experimental X-ray diffraction data revealed structural disorder of the N→O oxygen atom within the central heterocycle of compound **5**, quantum chemical calculations were systematically performed for two distinct isomers, **5a** and **5b**, which differ in the regiochemical position of the N-oxide group corresponding to the major and the minor disordered forms, respectively (Figure 4, Table 1, Tables S3 and S4).

The calculated Gibbs free energies (ΔG) in Table 1 explain the structural behavior of the system. In vacuum, all functionals show that major isomer **5a** is slightly more stable (ΔG = +0.04 to +0.22 kcal/mol), matching the *N*-oxide disorder observed via XRD. Polar medium simulation (water, CPCM) slightly favors minor form **5b** by −0.14 to −0.63 kcal/mol due to enhanced electrostatic stabilization. Ultimately, both isomers are thermodynamically almost identical, which explains the statistical distribution of the disordered oxygen atoms in the crystal lattice. Consequently, these conformers (**5a** and **5b**) were utilized to evaluate the correlation between the calculated and experimental XRD data in the gas phase and water, respectively. This thermodynamic equivalence confirms that both orientation isomers are virtually isoenergetic, providing a robust theoretical basis for the observed crystallographic disorder. The corresponding comparative analysis of geometric parameters is compiled in Tables S2–S4 (Supplementary Materials).

The comparative structural analysis of Compound **5** reveals a strong correlation between the experimental XRD parameters and the calculated data across all utilized functionals (M06-2X, ωB97X-D, and B3LYP-D3) in both media. The endocyclic and exocyclic bond lengths predicted in the gas phase and water replicate the experimental values with remarkable precision; for instance, the central furoxan ring bonds and the bridging carbonyl linkers show only minor deviations within approximately 0.01–0.03 Å. Furthermore, the valence bond angles in the central heterocycle and the nicotinoyl fragments are highly consistent with the crystallographic results, maintaining deviations well within 1.5–3.5°. The calculated torsion angles successfully reproduce the planar geometry of the central 1,2,5-oxadiazole ring (experimental τ ≈ 0.0–0.4° vs. theoretical τ < 0.5°). In addition, these parameters accurately map the rotation of the bridging carbonyl moieties and the spatial orientations of both disordered N-oxide oxygen atoms (**5a** and **5b**). Minor discrepancies between the computed and XRD data are primarily attributed to crystal lattice packing effects, which are naturally absent in isolated-molecule simulations.

**Figure 5:**
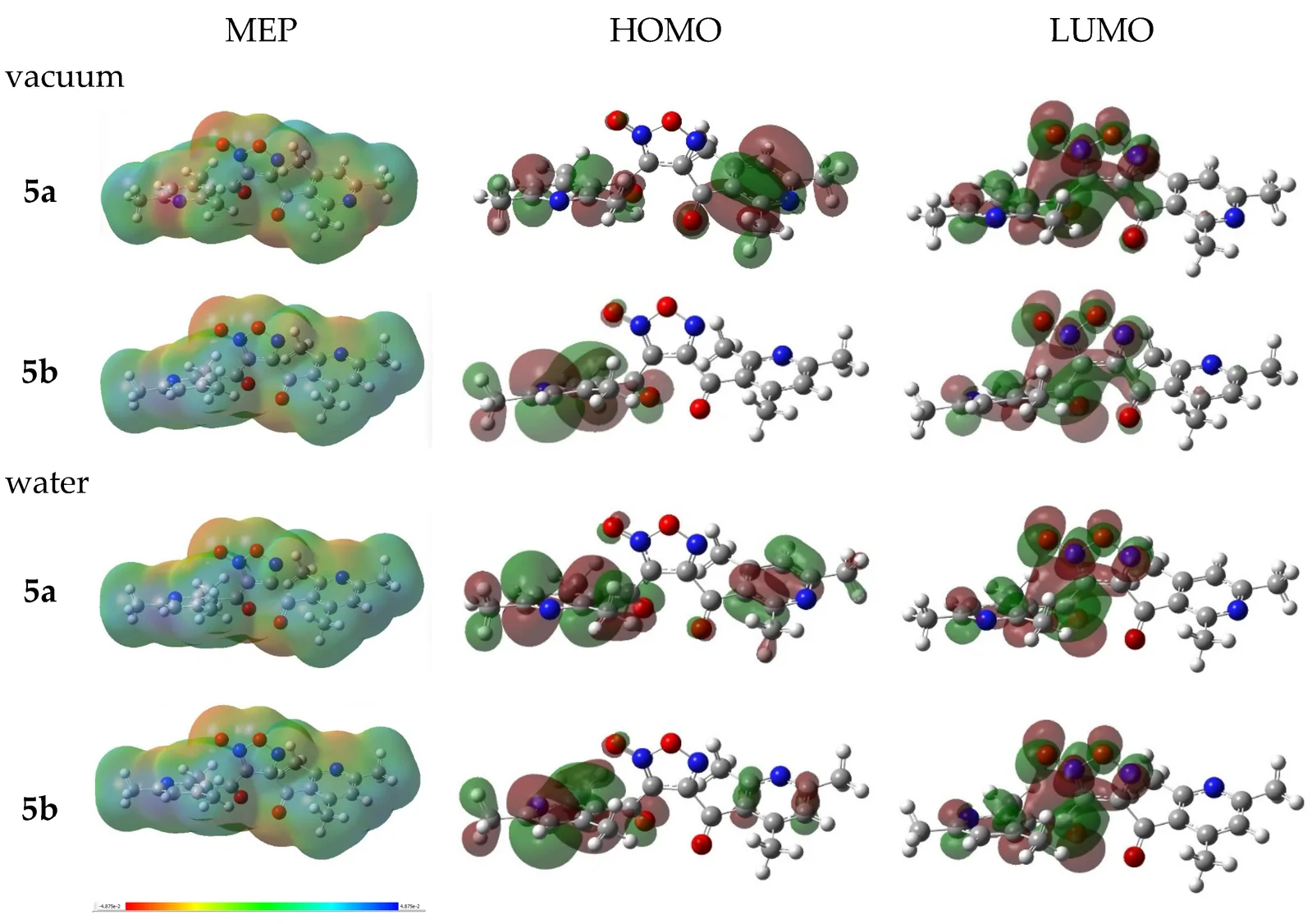
Molecular electrostatic potential (MEP) surfaces and spatial distribution of the HOMO/LUMO of conformers **5a** and **5b** calculated at the *ω*B97X-D/6-311++G(d,p) level in the gas phase (vacuum) and water (CPCM model). Isointensity surfaces for orbitals are mapped at 0.001 a.u.

Overall, the structural parameters computed across all three functionals show an exceptionally strong alignment with the crystallographic data, with ωB97X-D providing the highest precision for the core geometry in the gas phase and for the conformational twist in water (Table S5). Given its superior accuracy in reproducing both the framework and the spatial features of Compound **5**, the ωB97X-D/6-311++G(d,p) method was subsequently employed to evaluate the frontier molecular orbitals (HOMO/LUMO) [55] and molecular electrostatic potential (MEP) surfaces [56] (Figure 5, Tables S6 and S7).

The molecular electrostatic potential (MEP) surfaces of compound **5** clearly map its amphiphilic charge distribution, pinpointing the key nucleophilic and electrophilic centers. The primary nucleophilic sites are highly localized within the negative potential regions with red/yellow zones, surrounding the exocyclic oxygen atoms, specifically the polar N-oxide oxygen of the furoxan ring and the carbonyl C=O oxygen atoms, which act as potent hydrogen bond acceptors. Conversely, the peripheral areas, dominated by the electron-deficient methyl groups of the collidine rings and aromatic C–H protons, exhibit a strong positive potential with blue/light blue zones, establishing these regions as key electrophilic centers capable of participating in weak non-covalent interactions such as C–H...O hydrogen bonding and π-stacking.

The spatial distributions of the frontier molecular orbitals (HOMO and LUMO) for conformers **5a** and **5b** exhibit slight but important variations for understanding the structural features. While the overall topology of the orbital localization remains similar, with the HOMO density predominantly residing over the electron-rich furoxan core and the LUMO shifting toward the electrophilic carbonyl linkers and pyridine rings, the internal orientation of the orbital lobes closely follows the inversion of the N-oxide oxygen atom. The shift of the exocyclic oxygen from N2 (in **5a**) to N5 (in **5b**) alters the localized polarization of the central 1,2,5-oxadiazole ring, subsequently inducing minor asymmetry in the π-conjugation extended to the peripheral nicotinoyl branches. This delicate electronic reconfiguration, combined with the nearly identical Gibbs free energies of both forms, provides a robust quantum-chemical rationale for the statistical N-oxide disorder encountered in the crystal lattice, confirming that the molecular environment can easily accommodate both orbital states without disrupting the overall crystal packing.

Furthermore, the frontier molecular orbital analysis provides theoretical insights into the regioselectivity of the cyclization step (Scheme 5). As illustrated in Figure 5, the HOMO–LUMO frontier orbital distributions and electrostatic potentials demonstrate a pronounced spatial polarization and asymmetrical electron density distribution across the core and carbonyl linkers. In the framework of perturbation theory for chemical reactions, such strong electronic asymmetry and charge redistribution upon frontier orbital interactions promote effective orbital mixing along the reaction coordinate of the [3+2]-cycloaddition between nitrile oxide intermediates (**12**). This symmetry-breaking electronic perturbation probably lowers the activation barrier for the head-to-tail coupling pathway, thereby rationalizing the preferential formation of the asymmetric 3,4-diacylfuroxan framework **5** over alternative symmetrical reaction channels.

The calculated energy values of the frontier molecular orbitals (HOMO and LUMO), their corresponding energy gaps (ΔE), and derived global chemical reactivity descriptors for both conformers (**5a** and **5b**) in vacuum and water are summarized in Table 2.

As can be seen in Table 2, both isomers exhibit significant energy gaps ranging from 8.13 to 8.27 eV, which indicates high kinetic stability and pronounced chemical hardness (η = 4.07–4.14 eV) of the conjugated molecular system. The transition from the gas phase to the polar aqueous medium induces a systematic narrowing of the ΔE_HOMO_***_−_***_LUMO_ gap by approximately 0.08–0.10 eV for both forms, primarily driven by the enhanced electrostatic stabilization of the lowest unoccupied molecular orbitals (LUMO). Notably, the minor conformer **5b** features slightly lower energy gaps and marginally higher global chemical softness (S = 0.25 eV^−1^ in water) than the major form **5a** across both simulated environments. This subtle electronic differentiation suggests that while both conformers remain thermodynamically and chemically close, the minor form 5b possesses a slightly higher molecular polarizability, which correlates with its enhanced stabilization in a polar medium.

In summary, the combination of single-crystal X-ray diffraction analysis and DFT calculations provides a definitive stereochemical and electronic profile of compound **5**. The good convergence between the experimental crystallographic parameters and the geometries optimized via the ωB97X-D/6-311++G(d,p) functional validates the high reliability of the theoretical models. The structural disorder of the N-oxide oxygen atom observed in the crystal lattice finds its computational rationale in the thermodynamic near-degeneracy of conformers **5a** and **5b**, coupled with the flexible polarization of the central furoxan core, as evidenced by the molecular electrostatic potential (MEP) mapping. Furthermore, a wide frontier orbital gaps (>8.1 eV) and pronounced chemical hardness descriptors underscore the kinetic stability and low redox-reactivity of the synthesized compound **5**. Ultimately, the specific spatial distribution of the electrophilic and nucleophilic centers on the molecular electrostatic potential surfaces strongly implies a significant bioactive potential, which will be further evaluated in detail through subsequent in silico and in vitro studies.

### 2.3. In Silico Evaluation of Biological Activity Profiles of 3,4-bis(2,4,6-Trimethylnicotinoyl)-1,2,5-oxadiazole-2-N-oxide ***5***

To evaluate the pharmacological potential of the newly synthesized furoxan derivative (**5**), a virtual screening of its biological activity spectrum was performed for the major conformer **5a** using the PASS Online platform (Prediction of Activity Spectra for Substances https://way2drug.com/PassOnline/, accessed on 25 May 2026) [58]. This computational tool utilizes structural descriptors of chemical entities to predict their potential biological profiles, generating probability of presence (Pa) and probability that the compound belongs to the class of inactive compounds (Pi) for the predicted biological activity values for over 4000 distinct pharmacological effects [59]. These include transporter-related activities, specific pharmacotherapeutic outcomes, gene expression regulation, and intrinsic biochemical mechanisms (Table S8). The most significant predicted types of biological activity for compound **5**, along with their respective Pa and Pi parameters, are compiled in Table 3.

As summarized in Table 3, the in silico functional characterization of major conformer **5a** points toward a highly pronounced cardiotropic and hemorheological potential. The cardiotropic profile is dominated by exceptionally high probability values for Cardiotonic (Pa = 0.865) and Heart failure treatment (Pa = 0.823) activities, strongly suggesting that the synthesized furoxan framework holds potential for strengthening myocardial performance. This is further validated by predicted direct inotropic effects (Pa = 0.349), along with notable antianginal (Pa = 0.480) and coronary vasodilating (Pa = 0.357) mechanisms. At the molecular level, these systemic responses are supported by the predicted activation of voltage-sensitive calcium channels (Pa = 0.517). Crucially, the screening reveals a promising hemorheological performance, characterized by significant scores for both Platelet aggregation inhibitor (Pa = 0.505) and Platelet adhesion inhibitor (Pa = 0.369) activities. This dual antiplatelet and cardiotonic action is characteristic of nitric oxide-donating compounds and confirms that the core architectural assembly of compound **5** is highly optimized for target-specific cardiovascular evaluations. Also these findings match the previously discussed literature data for related furoxan-based nitric oxide donors and provide a solid state theoretical justification for the subsequent in vitro cardiotropic and antiplatelet evaluations.

To further elucidate the molecular mechanism underlying the predicted hemorheological and cardiotropic profiles, an inverse virtual screening was performed using the PASS Targets server (https://way2drug.com/passtargets/represent.php, accessed on 15 May 2026) [60]. The computational analysis identified key protein classes and biological processes highly correlated with the compound’s structure, most notably extracellular matrix organization and the metabolic process (Table S8). Among the probable targets, Matrix metalloproteinase-9 (MMP-9, ChEMBL321 [61]) emerged as the dominant candidate with the highest activity score (0.5879), predicted to act via an inhibitory pathway. Given the pivotal role of MMP-9 in endothelial dysfunction, vascular wall degradation, and post-ischemic myocardial remodeling, this enzyme was selected as the primary therapeutic target for deeper structural validation of compound **5** via a molecular docking approach using AutoDock Vina 1.2.0 [62,63].

Based on the ChEMBL321 recomendation and also to ensure the high reproducibility and structural robustness of the molecular docking experiment, three distinct crystal structures of the human MMP-9 catalytic domain were retrieved from the Protein Data Bank (PDB): **8K5Y** [64], **6ESM** [65], and **4XCT** [66]. The selection of these specific targets was rigorously driven by their satisfactory crystallographic resolution and diverse chemical profiles of their co-crystallized inhibitors, enabling a comprehensive cross-docking validation. Specifically, structure **6ESM** offers a high sub-atomic resolution of 1.10 Å, providing an exceptionally precise geometric baseline for the amino acid side chains within the active site. Structure **4XCT** (1.30 Å) features a co-crystallized classical hydroxamate-based inhibitor (ARP101), serving as a benchmark for zinc-coordinating interactions, while the recently resolved structure **8K5Y** (1.52 Å) provides a contemporary template of the catalytic pocket bound to an advanced indole-based derivative. Furthermore, a multi-tiered control protocol was integrated into the docking workflow to establish a definitive benchmark for the compound’s binding energy. The internal validation suite comprised the cross-docking of all three native (co-crystallized) ligands—VP6 (from **8K5Y**), B9Z (BE4, from **6ESM**), and N73 (ARP101, from **4XCT**)—across each protein matrix. Concurrently, three clinically and structurally relevant external standards were evaluated under identical simulation parameters: Doxycycline, an FDA-approved matrix metalloproteinase inhibitor [67]; Marimastat, a potent broad-spectrum hydroxamate inhibitor [68]; and Captopril, a zinc-chelating agent utilized to evaluate thiolated coordination dynamics within the catalytic center [69].

Prior to screening the target compounds, the molecular docking protocol was validated by re-docking the co-crystallized native ligands into their corresponding active sites. The protocol demonstrated high accuracy, successfully reproducing the crystallographic binding poses with heavy-atom RMSD values of 1.54 Å (8K5Y), 1.99 Å (6ESM), and 2.05 Å (4XCT), all accompanied by minimal centroid displacements (≤0.8 Å). These RMSD values confirmed the reliability of the grid box coordinates and docking parameters for subsequent virtual screening.

The comparative analysis of the binding affinities (ΔG) and Ligand Efficiency (LE) [70] of compound **5**, alongside a benchmark grid of co-crystallized inhibitors and reference drugs presented in Table 4.

The molecular docking results in Table 4 reveal that compound **5** exhibits an effective binding affinity across all tested protein conformations, demonstrating its competitive capability within the MMP-9 active site. Specifically, within the **8K5Y** domain, compound **5** displays binding free energy of −7.5 kcal/mol. This value outperforms the external clinical standards Doxycycline (−6.6 kcal/mol), Marimastat (−6.6 kcal/mol), and Captopril (−6.1 kcal/mol), while remaining competitive with the native ligand **VP6** (−13.1 kcal/mol). A similar trend is observed in the high-resolution structure **6ESM** and the **4XCT** matrix, where compound **5** shows binding energies of −7.2 kcal/mol and −8.0 kcal/mol, respectively. Notably, in these configurations, the affinity of compound **5** surpassing the inhibitory scores of Doxycycline (−7.2 and −6.2 kcal/mol), Marimastat (−6.0 and −6.7 kcal/mol), and Captopril (−5.2 and −5.6 kcal/mol), remaining competitive with the native ligand B9Z (BE4, −9.6 and −8.3 kcal/mol) and the native ligand N73 (ARP101, −8.7 and −8.3 kcal/mol).

Because binding free energy generally scales with molecular weight, Ligand Efficiency (*LE*) was calculated to evaluate the binding energy contributed per heavy atom (*N*_heavy_). A threshold of LE ≥ 0.3 kcal/mol/heavy atom is widely accepted as a standard benchmark for promising lead-like small molecules. Regarding Ligand Efficiency values, compound **5** (*N*_heavy_ =28) displayed a balanced profile with *LE* values ranging from 0.26 to 0.29 kcal/mol/heavy atom across all three targets (reaching its peak of 0.29 kcal/mol/heavy atom in 4XCT), indicating an optimized spatial and chemical interaction capability relative to its molecular mass. In comparison with reference drugs, compound **5** significantly outperformed doxycycline (LE = 020–0.22 kcal/mol/heavy atom), which suffers from high molecular weight (N_heavy_ = 32) without a proportional gain in affinity, while maintaining comparable or slightly superior efficiency to Marimastat (LE = 0.27–0.3 kcal/mol/heavy atom). Although captopril yielded higher nominal LE values (LE = 0.38–0.44 kcal/mol/heavy atom), this elevated metric is primarily an artifact of its exceptionally low heavy-atom count (N_heavy_ = 14) rather than superior potency, as evidenced by its overall modest binding affinities (ΔG ≥ −6.1 kcal/mol).

Overall, the combination of high binding affinity (ΔG ≤ −7.2 kcal/mol) and favorable ligand efficiency (LE ≈ 0.27–0.29 kcal/mol/heavy atom) highlights compound **5** as a lead candidate for MMP−9 inhibition, outperforming standard clinical controls in binding affinities.

**Figure 6:**
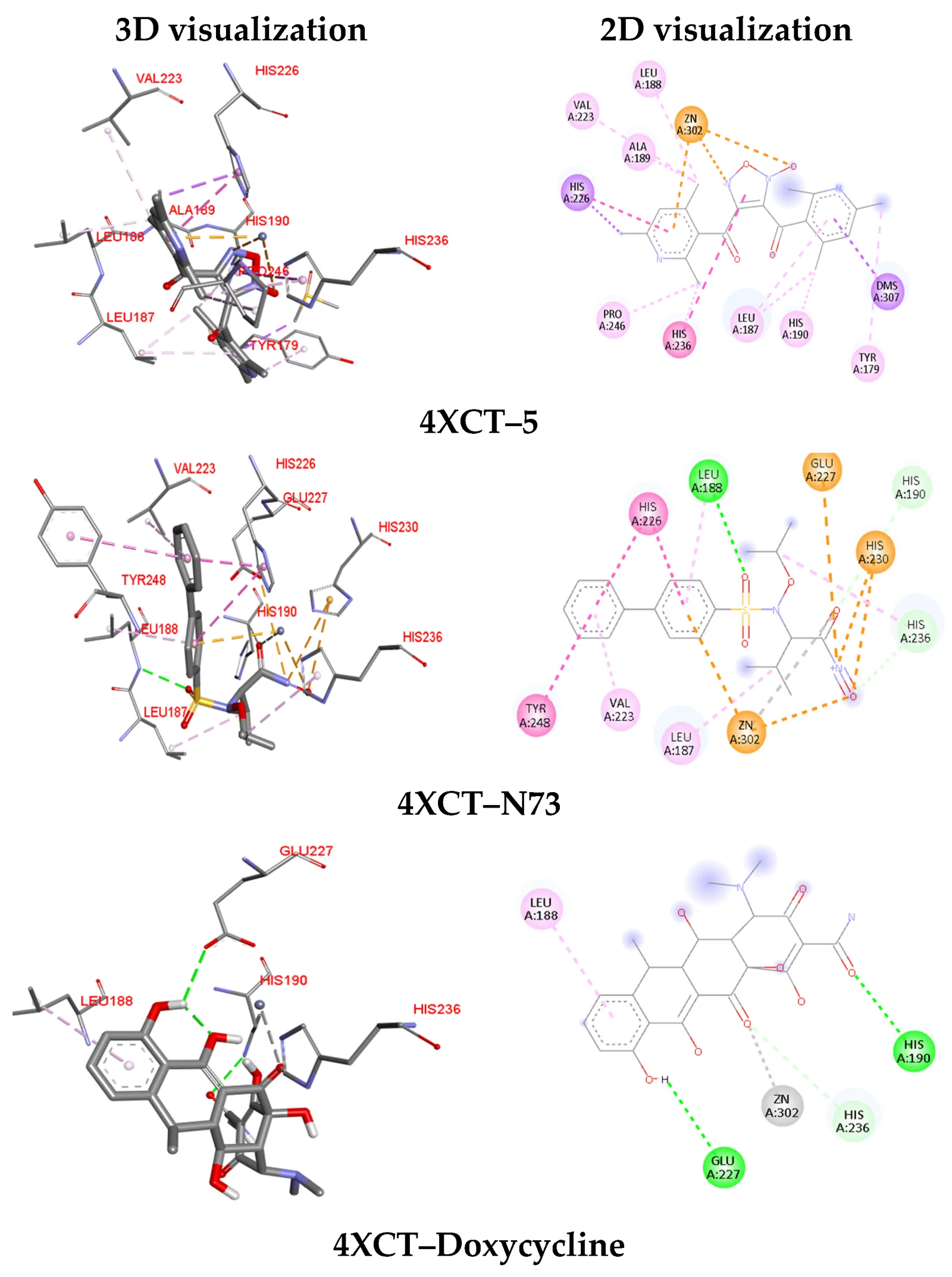
3D and 2D visualization of ligand–amino acid interactions of Compound **5** and Reference Standards within the Active Site of **4XCT** receptor. Residue labels indicate amino acid type, chain ID, and sequence number. Dashed lines and colored circles denote non-covalent interactions: green (conventional hydrogen bonds), orange (metal–acceptor coordination and π–cation interactions), and pink/purple (hydrophobic π–alkyl and π-π contacts).

To gain deeper, atomistic-level insights into the competitive binding modes and to map the non-covalent driving forces stabilizing the complexes, a comprehensive analysis of the intermolecular interaction networks was performed (Table S9, Figure S4). The specific amino acid residues involved in conventional hydrogen bonding, hydrophobic and electrostatic interactions across all simulated ligand-receptor configurations are cataloged in Table S9 and illustrated in Figure S4. For clarity of discussion, the key interaction parameters for compound **5** and representative reference standards are illustrated within the **4XCT** receptor site (Table 5; Figure 6), as this protein yielded the highest binding affinity (ΔG = −8.0 kcal/mol) and optimal ligand efficiency (LE = 0.29 kcal/mol/heavy atom) for the compound **5**, demonstrating superior binding potential compared to all reference drugs.

Analysis of the non-covalent interaction networks within the active sites (Table S9 and Table 5) reveals distinct structural determinants underlying the high binding affinities observed for compound **5**. In the primary target complex with **4XCT**, compound **5** establishes a robust binding profile anchored by a key π-cation contact with the zinc ion (Zn302), mirroring the metal-coordination motifs observed for native reference ligands (**N73** and **VP6**). Furthermore, compound **5** engages in extensive hydrophobic stabilization within the **4XCT** pocket via π-alkyl networks with key residues (**LEU188**, **VAL223**, **ALA189**, **PRO246**, **LEU187**, **HIS190**, and **TYR179**), alongside π-sigma interactions with **HIS226** and π-π stacked interactions with **HIS236**.

Notably, while native ligands and reference drugs rely heavily on conventional hydrogen bonding networks (such as **LEU188** for **N73**, and **GLU227**/**HIS190** for doxycycline), the binding driven by compound **5** across all three MMP-9 targets is predominantly governed by strong lipophilic and coordination-driven contacts, effectively avoiding unfavorable donor–donor or acceptor–acceptor steric clashes and maintaining high structural reproducibility across variable protein conformations.

In summary, in silico profiling elucidated the molecular interaction patterns underlying the binding potential of compound **5**. The alignment between the PASS Online predictions and the molecular docking simulations confirms that this novel furoxan derivative forms a highly stable and energetically favorable non-covalent network within the catalytic site of MMP-9. The in silico predicted MMP-9 inhibition by compound **5** underlines its potential to mitigate extracellular matrix degradation and endothelial barrier disruption, thereby suppressing vascular inflammation and adverse structural remodeling—key mechanisms that support its targeted cardioprotective and hemorheological profile. These in silico insights provide a theoretical rationale for the subsequent experiment in vitro evaluations of its hemorheological activity.

### 2.4. In Vitro Evaluation of Hemorheological Activity of 3,4-bis(2,4,6-Trimethylnicotinoyl)-1,2,5-oxadiazole-2-N-oxide

For the initial assessment of the hemorheological activity of the studied compounds, an in vitro model of high blood viscosity syndrome (HBVS) was used, specially designed to reproduce key pathological changes in the rheological properties of blood characteristic of various clinical conditions. The use of this model is due to its high reproducibility, physiological relevance, and its ability to quantify the effects of the tested compounds. The hyperviscosity model was reproduced by incubating blood samples at a temperature of 43.0 °C for 60 min. It was found that exposure to these temperature conditions leads to a significant increase in blood viscosity due to increased aggregation of red blood cells and a decrease in their deformability. Such changes are key mechanisms for the development of hemorheological disorders characteristic of a wide range of vascular and metabolic pathologies [71]. This protocol made it possible to minimize the effect of the solvent on the rheological properties of blood and to objectively evaluate the effect of the tested compounds. The use of this in vitro model made it possible to simulate HBVS in a laboratory experiment, which is an important tool for early detection and pre-screening of substances with potential hemorheological effects (Table 6).

The results in Table 6 demonstrate the effectiveness of the in vitro blood hyperviscosity model for detecting compounds with promising hemorheological activity. Comparison with the reference drug (pentoxifylline) demonstrated the expected hemorheological effect, thereby confirming the reliability, reproducibility, and physiological relevance of the chosen screening methodology. Meanwhile 3,4-bis(2,4,6-trimethylnicotinoyl)-1,2,5-oxadiazole-2-N-oxide showed the hemorheological activity, contributing to a decrease in blood viscosity under conditions of induced hyperviscosity in vitro.

The experimentally observed in vitro hemorheological activity of compound **5** under induced hyperviscosity conditions provides a robust phenotypic validation of the prior in silico target predictions. The capacity of the synthesized furoxan derivative to significantly decrease blood viscosity compared to the reference drug (pentoxifylline) strongly correlates with its predicted inhibitory mechanism toward Matrix metalloproteinase-9 (MMP-9). At the cellular level, MMP-9 is known to modulate the degradation of cell-surface receptors and extracellular components, directly affecting erythrocyte deformability and accelerating platelet-fibrin interactions under rheological stress. By selectively suppressing MMP-9 pathways, compound **5** likely preserves the structural integrity of erythrocyte membranes and prevents micro-aggregation of blood cells. This alignment between the in silico insights and the verified in vitro blood-viscosity reduction firmly establish compound **5** as a promising multi-target hemorheological agent with an effective mechanism of action.

## 3. Materials and Methods

### 3.1. Reagents and Synthetic Procedures

^1^H and ^13^C NMR spectra were recorded on a Jeol JNM-ECA 400 spectrometer (JEOL Ltd., Tokyo, Japan) (400 and 101 MHz, respectively) and Bruker AVANCE 500 spectrometer (Bruker BioSpin GmbH, Rheinstetten, Germany) (500 and 126 MHz, respectively) instruments using CDCl_3_. The internal standard was TMS or residual solvent signals (7.26 and 77.0 ppm for ^1^H and ^13^C nuclei in CDCl_3_).

Chromato-mass spectrometric studies were carried out on a Trace GC Ultra chromatograph (Thermo Fisher Scientific, Milan, Italy) with a DSQ II mass-selective detector (Thermo Fisher Scientific, Austin, TX, USA) in the electron ionization mode (70 eV) on a Thermo TR-5 MS quartz capillary column (Thermo Fisher Scientific, Bellefonte, PA, USA), 15 m long, 0.25 mm inner diameter, with a film thickness of the stationary phase of 0.25 μm. Splitless input mode was used. Carrier gas discharge 20 mL/min. The velocity of the carrier gas (helium) was 1 mL/min, evaporator temperature was 200 °C, transition chamber temperature was 200 °C, and ion source temperature was 200 °C. The temperature of the column thermostat was changed according to the program: from 15 (5 min delay) to 220 °C at a rate of 20 °C per minute, to 290° at a rate of 15° per minute. The total analysis time was 30 min. The volume of the injected sample is 1 μL. Chromatograms were recorded in TIC mode. The range of mass scanning was 30–450 amu.

Melting points were determined using a Stuart SMP10 hot bench. Monitoring of the reaction course and the purity of the products was carried out by TLC on Sorbfil plates and visualized using iodine vapor or UV light.

1-(2,4,6-trimethylpyridin-3-yl)ethan-1-one **3** was synthesized according to published procedures [17].

Regarding 3,4-bis(2,4,6-trimethylnicotinoyl)-1,2,5-oxadiazole-2-*N*-oxide **5**, a solution of 1.63 g (10.0 mmol) of 3-acetyl-2,4,6-trimethylpyridine **3** in 2 mL of AcOH was added dropwise to a nitrating mixture of 5.1 g (50.0 mmol) KNO_3_ and 2.7 mL (50.0 mmol) H_2_SO_4_ with stirring in an ice bath. At the end of the addition, the pyridine mixture was stirred at 60–70 °C for 24 h. Upon completion of the addition (TLC and GC-MS monitoring), the mixture was poured onto ice water, neutralized with potash, and extracted with EtOAc (3 × 20 mL). The combined organic layers were washed with saturated NaCl solution and dried over anhydrous Na_2_SO_4_. After evaporation of the solvent under vacuum, the oil was added and purified by recrystallization from hexane:methylene chloride (4:1) mixture; yield: 250 mg (12%), white crystals, mp 95–97 °C. ^1^H NMR (400 MHz, CDCl_3_) δ ppm 2.23 (s, 3H, 2-CH_3_), 2.28 (s, 3H, 2′-CH_3_), 2.42 (s, 3H, 6-CH_3_), 2.44 (s, 3H, 6′-CH_3_), 2.49, 2.50 (2 s, 6H, 4,4′-CH_3_), 6.92 (s, 2H, H-6,6′ Py). ^13^C NMR (101 MHz, CDCl_3_) δ ppm 19.2 (CH_3_), 19.6 (CH_3_), 22.9 (CH_3_), 23.6 (CH_3_), 24.6 (CH_3_), 112.0, 122.9, 123.0, 128.8, 129.3, 146.2, 146.4, 154.5, 154.8, 155.1, 160.6, 160.7, 183.7 (C=O), 187.1 (C=O). MS (EI) m/z (I_rel_, %): [M]^+^ 364.07 (6.5), 200.03 (9.5), 148.08 (100), 120.05 (67.8). Anal. calcd for C_20_H_20_N_4_O_4_: C, 63.35; H, 5.15; N, 14.90; found: C, 63.15; H, 5.30; N, 14.73.

Synthetic method for 1-(2,4,6-trimethylpyridin-3-yl)ethan-1-ol **6** was previously described by our group of researchers [34]. To a solution of 3-acetyl-2,4,6-trimethylpyridine **3** (2.0 g, 12.0 mmol) in 8 mL of EtOH-water (5:3), NaBH_4_ (0.9 g, 24.0 mmol) was added with stirring in portions. Stirring was maintained for 2 h after all NaBH_4_ was added, then water (50 mL) was added, and the mixture extracted with EtOAc (3 × 20 mL). The combined organic layers were washed with saturated NaCl solution and dried over anhydrous Na_2_SO_4_. The solvent was evaporated under reduced pressure, and the residue was purified by recrystallization from hexane; yield: 1.8 g (90%), white crystals, mp 94–95 °C. ^1^H NMR (500 MHz, CDCl_3_) δ ppm 1.48 (d, J = 6.8 Hz, 3H, -CHCH_3_), 2.38 (2 s, 6H, 4-CH_3_, 6-CH_3_), 2.48 (s, 3H, 2-CH_3_), 5.28 (q, J = 6.7 Hz, 1H, -CH), 6.73 (s, 1H, H-5). ^13^C NMR (126 MHz, CDCl_3_) δ ppm 20.1, 21.8, 23.2, 23.7, 66.3, 124.3, 133.6, 145.7, 154.9, 155.5. MS (EI) m/z (I_rel_, %): [M]^+^ 165.00 (20), 150.04 (100), 122.11 (44). Anal. calcd for C_10_H_15_NO: C, 72.53; H, 9.37; N, 8.65; found: C, 72.69; H, 9.15; N, 8.48.

### 3.2. Molecular Structure of 3,4-bis(2,4,6-Trimethylnicotinoyl)-1,2,5-oxadiazole-2-N-oxide ***5*** XRD and DFT Study

The X-ray diffraction experiments were carried out at 296(2) K on a Bruker KAPPA APEX II CCD diffractometer (graphite-monochromated Mo Kα radiation). Reflection intensities were corrected for absorption by SADABS2008/1 program [72]. The structures were solved by direct methods using the SHELXS-97 [73] and refined by anisotropic (isotropic for all H atoms) full-matrix least-squares method against *F*^2^ of all reflections by SHELXL2018/3 [74]. The positions of the hydrogen atoms were calculated geometrically and refined in riding model. The N→O oxygen atom within the central heterocycle of compound **5** is statistically disordered at approximate ratio ~4:1. The determination of intermolecular contacts was done with PLATON program [75].

Crystallographic data for the structure **5** have been deposited at the Crystallographic Data Centre as supplementary publication no. CCDC 2572584. Copy of the data can be obtained, free of charge, on application to CCDC, 12 Union Road, Cambridge CB21EZ, UK (fax: +44 122 3336033 or e-mail: deposit@ccdc.cam.ac.uk; internet: www.ccdc.cam.ac.uk, accessed on 12 June 2026).

*Crystallographic data for* **5**: C_20_H_20_N_4_O_4_, *M* 380.40, triclinic, $P\bar{1}$, *a* 8.4541(4), *b* 9.2837(4), *c* 13.2823(6) Å, α 77.403(2), β 76.916(2), γ 80.939(2)°, *V* 984.53(8) Å^3^, *Z* 2, *D*_calcd_ 1.283 g·cm^–3^, *μ*(Mo-*K*α) 0.092 mm^–1^, F(000) 400, (θ 2.26–26.19°, completeness 99.9%), colorless, (0.38 × 0.31 × 0.23) mm^3^, transmission 0.943–0.971, 26,189 measured reflections in index range −10 ≤ h ≤ 10, −11 ≤ k ≤ 11, −16 ≤ l ≤ 16, 3954 independent (*R*_int_ 0.037), 264 parameters, one restraint, *R*_1_ 0.0575 (for 3087 observed *I* > 2*σ*(*I*)), *wR*_2_ 0.1829 (all data), GOOF 1.069, largest difference peak and hole ΔQ_max_ 0.31 and ΔQ_min_ −0.19 e.A^−3^.

Geometry optimizations and frequency calculations (OPT + FREQ) were performed using Gaussian 16 software [45]. Molecular structures were built and visualized in GaussView 6.0 [76]. Full geometry optimizations followed by vibrational frequency calculations (OPT + FREQ) [46] of compound (**5**), starting from the respective CIF data, were systematically carried out both in the gas phase (vacuum) and in a solvent medium (water) utilizing the Conductor-like Polarizable Continuum Model (*CPCM*) [47]. For a comprehensive comparative analysis, three density functional theory (DFT) methods were employed: M06-2X/def2-TZVP [48,49], ωB97X-D/6-311++G(d,p) [50,51], and B3LYP-D3/6-311++G(d,p) [52,53,54]. All optimized geometries were confirmed as true minima by the absence of imaginary frequencies.

HOMO and LUMO frontier molecular orbitals were visualized to assess electron distribution, energy gaps, and donor–acceptor potential [77]. Orbital localization was analyzed to identify reactive centers and evaluate π-conjugation [78]. Molecular electrostatic potential (MEP) maps were generated on the electron density isosurface (0.001 a.u.) to visualize charge distribution.

### 3.3. In Silico Evaluation of Biological Activity Profiles of 3,4-bis(2,4,6-Trimethylnicotinoyl)-1,2,5-oxadiazole-2-N-oxide ***5***

In silico evaluation of the biological activity potential was performed using online predictive analytics tools and molecular docking techniques [79]. The obtained DFT optimized compound **5** molecular geometry was saved in .mol format for PASS analysis and also converted to the .pdb format using Chem3D 22.2.0 [80] for subsequent molecular docking.

#### 3.3.1. PASS Prediction, Target Protein Selection, and Ligand Dataset Preparation

PASS (Prediction of Activity Spectra for Substances) analysis [60] was performed using PASS online platform, available at http://way2drug.com/PassOnline/predict.php, accessed on 25 May 2026. Predictive outputs from PASS are expressed as Pa and Pi probability scores [61]. The Pa score, or probability “to be active,” ranges from 0 to 1 and estimates the likelihood that a compound belongs to the class of biologically active substances, based on structural similarity to known actives in the PASS training set. Conversely, the Pi score, or the probability that the compound belongs to the class of inactive compounds for the predicted biological activity also ranging from 0 to 1, reflects the likelihood that the compound resembles structures typical of inactive substances. To identify target proteins, the inverse virtual screening was performed using the PASS Targets server (https://way2drug.com/passtargets/represent.php, accessed on 25 May 2026) [62]. Based on the PASS Targets server recommendation, three distinct crystal structures of the human MMP-9 catalytic domain were retrieved from the Protein Data Bank (PDB): **8K5Y** [64], **6ESM** [65], and **4XCT** [66]. The internal validation suite comprised the cross-docking of all three native (co-crystallized) ligands—VP6 (from **8K5Y**), B9Z (BE4, from **6ESM**), and N73 (ARP101, from **4XCT**)—across each protein matrix. Concurrently, three clinically and structurally relevant external standards were evaluated under identical simulation parameters: Doxycycline, an FDA-approved matrix metalloproteinase inhibitor [67]; Marimastat, a potent broad-spectrum hydroxamate inhibitor [68]; and Captopril, a zinc-chelating agent utilized to evaluate thiolated coordination dynamics within the catalytic center [69].

#### 3.3.2. Molecular Docking and Validation Protocol

Molecular docking simulations were performed using AutoDock Vina 1.2.0 and AutoDock MGL Tools 1.5.7 (Molecular Graphics Laboratory, the Scripps Research Institute, La Jolla, CA, USA) [62,63]. A semi-flexible docking protocol was applied, wherein the protein receptors were kept rigid to preserve their crystal conformations, while the ligand molecules were treated as fully flexible, allowing freedom for rotatable bonds [81]. The docking calculations were performed using the Monte Carlo iterated local search algorithm coupled with the Broyden–Fletcher–Goldfarb–Shanno (BFGS) gradient-based local optimization, as implemented in AutoDock Vina [82].

Three-dimensional structures of matrix metalloproteinase-9 (MMP-9; PDB IDs: 4XCT, 6ESM, and 8K5Y) were retrieved from the Protein Data Bank. Receptor preparation involved the removal of co-crystallized solvent molecules and non-catalytic native ligands, addition of polar hydrogen atoms, and assignment of Partial Atomic Charges at physiological pH (pH 7.4). For each receptor, a receptor-only structure was generated. The native ligands were extracted, saved separately, and prepared for redocking validation.

The active site coordinates were defined based on the centroids of the respective crystallographic native ligands: center (x = 18.200, y = 8.080, z = −15.110) with grid box size 24 Å × 24 Å × 24 Å for MMP-9 (PDB ID: 8K5Y); center (x = 1.781, y = 50.977, z = 19.670) with grid box size 28 Å × 28 Å × 28 Å for MMP-9 (PDB ID: 6ESM); and center (x = 17.852, y = −17.722, z = 19.188) with grid box size 28 Å × 28 Å × 28 Å for MMP-9 (PDB ID: 4XCT).

To validate the docking procedure, native ligands were independently re-docked back into their respective uncomplexed binding sites under identical search conditions. Validation success was determined using two criteria: structural agreement evaluated via root-mean-square deviation (RMSD) of all heavy atoms between the top-ranked docked pose and the original crystallographic binding mode after optimal superposition (Kabsch algorithm [83]), with a threshold of RMSD ≤ 2.0 Å (or acceptable spatial overlap within ≤ 2.05 Å); reservation of binding site placement was defined as a centroid displacement ≤ 5.0 Å.

In addition to binding energy (ΔG, kcal/mol), Ligand Efficiency (LE) was calculated to normalize binding affinity against molecular size according to Equation (1):

(1) $$LE=-\frac{\Delta G}{N_{\mathrm{heavy}}}$$

where *N*_heavy_—is the number of non-hydrogen (heavy) atoms in the ligand molecule [70].

During the validation of **PDB ID 8K5Y**, a structural anomaly was identified and resolved: the native ligand VP6 is duplicated in the crystallographic file (Chain A in the functional catalytic site and Chain B at an artifactual crystal contact site). Initial grid placement based on Chain B improperly targeted the external area, resulting in a poor score (ΔG = −6.1 kcal/mol). Re-centering the grid box strictly on the catalytic pocket of Chain A (x = 18.200, y = 8.080, z = −15.110) successfully restored the true crystallographic pose, yielding RMSD = 1.54 Å, an improved affinity of ΔG = −13.2 kcal/mol, and LE = 0.38 kcal/mol/heavy atom. The remaining native complexes also confirmed protocol validity: **6ESM/B9Z:** RMSD = 1.99 Å, ΔG = −10.4 kcal/mol, LE = 0.36 kcal/mol/heavy atom; **4XCT/N73:** RMSD = 2.05 Å, ΔG = −9.8 kcal/mol, LE = 0.34 kcal/mol/heavy atom (Table 7).

Following protocol validation, all test compounds (Compound **5**, VP6, B9Z, N73, doxycycline, Marimastat, and captopril) were docked into the validated binding sites under semi-flexible conditions with the following search parameters: exhaustiveness = 32, num_modes = 20, and energy_range = 4 kcal/mol.

Intermolecular interactions (conventional/carbon hydrogen bonds, hydrophobic contacts, π-cation, π-anion, π-alkyl, and coordination with zinc ions) were categorized and visualized using BIOVIA Discovery Studio Visualizer 2021 [84].

### 3.4. In Vitro Evaluation of Hemorheological Activity of 3,4-bis(2,4,6-Trimethylnicotinoyl)-1,2,5-oxadiazole-2-N-oxide

Hyperviscosity syndrome was reproduced in vitro by blood incubation at 43.0 °C for 60 min. Blood viscosity was measured on a Brookfield DV2T rotational viscometer at various spindle speeds (from 2 to 60 rpm). This approach provides a comprehensive assessment of the effect of compounds on both the deformability of erythrocytes at high shear rates and their aggregation properties at low rates [71]. Studies of the hemorheological activity of 3,4-bis(2,4,6-trimethylnicotinoyl)-1,2,5-oxadiazole-2-N-oxide were carried out on 9 Wistar female rats, 12 weeks old, weighing 220–240 g. After blood sampling, the initial blood viscosity was determined in laboratory animals, and then blood samples were incubated with the test substance at a temperature of 43.0 °C for 60 min and then measured the parameters under study. The blood was incubated with the 3,4-bis(2,4,6-trimethylnicotinoyl)-1,2,5-oxadiazole-2-N-oxide dissolved in DMSO; the final concentration of the compounds was 10^−5^ g/mL of blood. Blood samples to which DMSO solvent was added in an equivolume amount served as controls. As a reference drug, a substance with known hemorheological properties, pentoxifylline, was used at a concentration of 10^−5^ g/mL of blood [85]. Blood incubation for 1 h under these conditions was accompanied by the formation of blood hyperviscosity [71]. The initial blood viscosity from each animal was measured once; the blood viscosity after incubation was measured in two samples from each animal, both in control and experimental samples.

### 3.5. Ethical Considerations

The whole research work with laboratory animals was performed in accordance with generally accepted ethical standards for the treatment of animals, based on standard operating procedures that comply with the rules adopted by the European Convention for the Protection of Vertebrate Animals used for Research and other Scientific Purposes (Strasbourg, 1986 [86]). The study protocol of the project “Search for means of pharmacological correction of the syndrome of increased blood viscosity associated with endocrine pathology” was approved on August 07, 2020 by the Local Ethical Commission of the National Center for Biotechnology (IRB00013497 National Center of Biotechnology IRB #1).

### 3.6. Statistical Analysis

Statistical processing of the hemorheological data was carried out using the Microsoft Excel program, with data representation in the “average ± standard error of the average” format, which meets the requirements of international standards for biomedical data processing.

## 4. Conclusions

This work demonstrates an unexpected pseudo-multicomponent transformation of sterically hindered 3-acetyl-2,4,6-trimethylpyridine (**3**), providing access to the highly functionalized bis(nicotinoyl)furoxan derivative **5**. While the initial objective aimed at the selective nitration of the pyridine scaffold, the reaction consistently triggered an oxidative assembly sequence involving nitrozation, in situ nitrile oxide generation, and subsequent [3+2]-cycloaddition to construct the central furoxan core. This unusual chemical behavior is presumably driven by the specific electronic and steric features of the collidine matrix alongside the thermodynamic stability of the resulting 3,4-bis(2,4,6-trimethylnicotinoyl)-1,2,5-oxadiazole 2-oxide (**5**).

Single-crystal X-ray diffraction analysis coupled with DFT calculations provided a definitive structural and electronic profile of compound **5**. The good agreement between the crystallographic parameters and geometries optimized at the ωB97X-D/6-311++G(d,p) level validates the computational model. Crucially, the observed orientational disorder of the N-oxide oxygen atom within the crystal lattice was computationally rationalized by the thermodynamic near-degeneracy (ΔG < 0.63 kcal/mol) of conformers **5a** and **5b**.

Importantly, the frontier molecular orbital analysis provided theoretical insights into the regioselectivity of the cyclization step (Scheme 5). Within the framework of perturbation theory, the strong spatial polarization of the frontier molecular orbitals lowers the activation barrier for the head-to-tail [3+2]-cycloaddition of nitrile oxides (**B** and **B’**), thereby rationalizing the preferential formation of the asymmetric 3,4-diacylfuroxan **5**. Additionally, wide frontier orbital energy gaps (8.13–8.27 eV) and chemical hardness descriptors (η = 4.07–4.14 eV) underscore the high kinetic stability of the synthesized heterocycle.

In silico profiling successfully elucidated the pharmacophore network driving the biological potential of compound **5**. PASS Online predictions combined with molecular docking across diverse Matrix metalloproteinase-9 (MMP-9) crystallographic models confirmed that this furoxan derivative effectively anchors within the catalytic pocket through conventional hydrogen bonds and extensive π-mediated hydrophobic interactions.

In vitro evaluations confirmed the high hemorheological efficacy of compound **5**, which significantly decreased blood viscosity under induced hyperviscosity conditions, systematically outperforming the reference drug pentoxifylline.

The comprehensive structural characterization, theoretical rationale, and functional biological evaluation firmly establish compound **5** as a novel and promising heterocyclic framework with potential hemorheological and cardioprotective applications.

## Figures and Tables

**Table 1:** Calculated Gibbs Free Energies (*G*) and Relative Energies (Δ*G*) for Isomers **5a** and **5b** in Gas Phase and Water.

	G, A.U	Vacuum			CPCM (Water)		
Isomeric Form		M062X	ωB97XD	B3LYP-D3	M062X	ωB97XD	B3LYP-D3
**5a** (Major)		**−1293.583688**	**−1293.538237**	−1294.029401 *	−1293.599446	−1293.555804	−1294.045215
**5b** (Minor)		−1293.583421	−1293.537886	−1294.029333	**−1293.599674**	**−1293.556804**	−1294.045741 *
ΔG, A.U.		0.000267	0.000351	0.000068	−0.000228	−0.001	−0.000526
ΔG, kcal/mol		0.167545036	0.220255834	0.042670646	−0.143072166	−0.6275095	−0.330069997

* Bold values indicate lower Gibbs free energy values (G), corresponding to the thermodynamically more stable isomer.

**Table 2:** Calculated Energy Values of Frontier Molecular Orbitals (HOMO/LUMO), Energy Gaps (ΔE), and Global Reactivity Descriptors for Conformers **5a** and **5b**.

	Parameter	E_HOMO_ (a.u.)	E_LUMO_ (a.u.)	ΔE_HOMO−LUMO_ (a.u.)	ΔE_HOMO−LUMO_ (eV) *	η (eV) *	S (eV^−1^) *
Conformer							
Gas Phase (Vacuum)							
Conformer **5a**		−0.33933	−0.03537	0.30396	8.27	4.14	0.24
Conformer **5b**		−0.33706	−0.03542	0.30164	8.21	4.11	0.24
Water (CPCM model)							
Conformer **5a**		−0.34061	−0.04045	0.30016	8.17	4.09	0.24
Conformer **5b**		−0.33971	−0.04111	0.2986	8.13	4.07	0.25

* ΔE = E_LUMO_ − E_HOMO_; Global chemical hardness η = ΔE/2; Global chemical softness S = 1/(2η) [57]. Conversion factor: 1 a.u. = 27.2114 eV.

**Table 3:** Predicted Biological Activity Profiles of Compound **5**.

Compound	Types of Biological Activity	Pa *	Pi *
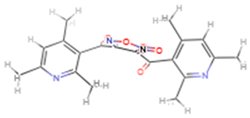 **5a**	Cardiovascular/Cardiotropic **		
	Cardiotonic	0.865	0.004
	Heart failure treatment	0.823	0.004
	Calcium channel (voltage-sensitive) activator	0.517	0.067
	Antianginal	0.48	0.047
	Vasodilator, coronary	0.357	0.079
	Inotropic	0.349	0.021
	Calcium channel activator	0.33	0.029
	Hemorheological/Antiplatelet **		
	Platelet aggregation inhibitor	0.505	0.008
	Platelet adhesion inhibitor	0.369	0.195

* where Pa is the probability of presence of biological activity, and Pi is the probability that the compound belongs to the class of inactive compounds for the predicted biological activity. ** Italicized rows highlight general pharmacological activity classes.

**Table 4:** Binding Affinities (ΔG, kcal/mol) and Ligand Efficiency (LE) of Compound **5** Against Matrix Metalloproteinase-9 (MMP-9) in Comparison with Native Ligands and Reference Drugs.

No.	Compound	Chemical Formula	*N*_heavy_	Binding Affinity Δ*G* (kcal/mol)			LE (kcal/mol/ Heavy Atom) *		
				8K5Y	6ESM	4XCT	8K5Y	6ESM	4XCT
1	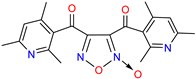 **5**	C_20_H_20_N_4_O_4_	28	−7.5	−7.2	−8.0	0.27	0.26	0.29
	Native (Co-crystallized) Ligands								
2	Native ligand **VP6**	C_21_H_23_FN_4_O_2_	28	−13.1	−7.7	−8.6	0.47	0.28	0.31
3	Native ligand **B9Z** (BE4)	C_24_H_22_O_5_S	30	−8.7	−9.6	−8.3	0.29	0.32	0.28
4	Native ligand **N73** (ARP101)	C_20_H_24_N_2_O_5_S	28	−8.6	−8.7	−8.3	0.31	0.32	0.3
	Reference Drugs (External Standards)								
5	Doxycycline	C_22_H_24_N_2_O_8_	32	−6.6	−7.0	−6.2	0.21	0.22	0.2
6	Marimastat	C_15_H_29_N_3_O_5_	23	−6.0	−6.0	−6.7	0.27	0.27	0.3
7	Captopril	C_9_H_15_NO_3_S	14	−6.1	−5.2	−5.6	0.44	0.38	0.4

* LE = −ΔG/*N*_heavy_, where *N*_heavy_-is the number of non-hydrogen heavy atoms [70]. A value of LE ≥ 0.3 kcal/mol/heavy atom is generally considered as a good LE value for small molecules.

**Table 5:** Intermolecular Interactions of Compound **5** and Reference Standards within the Active Site of **4XCT** receptor.

	Ligand	5	VP6	B9Z	N73	Doxycycline	Marimastat	Captopril
Interaction								
**Conventional Hydrogen Bond**		-	ARG249	HIS190	LEU188	GLU227, HIS190	MET247	-
**Carbon Hydrogen Bond**		-	HIS236	-	HIS190, HIS236	HIS236	ALA189	LEU187
**π-Alkyl**		LEU188,VAL223, ALA189,PRO246, LEU187,HIS190, TYR179	LEU222, VAL223, LEU243	LEU222, VAL223, LEU188	VAL223, LEU187	LEU188	LEU188	LEU188
**π-Cation**		Zn302	Zn302	-	Zn302, HIS230	-	-	-
**π-Anion**		-	-	-	GLU227	-	-	-
**π-Sigma**		HIS226,DMS307	-	-	-	-	-	HIS226
**π-Sulfur**		-	MET247	-	-	-	-	TYR248
**π-π T-shaped**		HIS236	-	HIS236	TYR248	-	-	-
**π-π Stacked**		HIS236	HIS226	TYR248, HIS226	HIS226	-	-	-
**Metal-Acceptor**		-	-	Zn302	Zn302	Zn302	Zn302	-
**Unfavorable** **Donor-Donor**		-	TYR248	-	-	-	-	-

**Table 6:** Hemorheological effects of 3,4-bis(2,4,6-trimethylnicotinoyl)-1,2,5-oxadiazole-2-N-oxide: changes in blood viscosity under in vitro hyperviscosity model conditions.

Characteristic	Blood Viscosity at Different Spindle Speeds (rpm), MPa ×s							
	2	4	6	8	12	20	40	60
Screening of 3,4-bis(2,4,6-trimethylnicotinoyl)-1,2,5-oxadiazole-2-N-oxide (*n* = 3)								
Initial	3.68 ± 0.09	3.44 ± 0.06	3.19 ± 0.04	3.07 ± 0.04	2.43 ± 0.02	2.29 ± 0.02	2.22 ± 0.04	2.19 ± 0.04
After incubation in control	8.41 ± 0.44 *p*1 = 0.0002	7.30 ± 0.42 *p*1 = 0.0004	5.86 ± 0.23 *p*1 = 0.0001	4.65 ± 0.21 *p*1 = 0.0014	4.00 ± 0.10 *p*1 = 0.00001	3.64 ± 0.03 *p*1 = 0.00000001	3.54 ± 0.02 *p*1 = 0.00000001	3.45 ± 0.02 *p*1 = 0.000000005
After incubation with compound KVD-19	7.18 ± 0.68 *p*1 = 0.0096 *p*2 = 0.1560	5.72 ± 0.42 *p*1 = 0.0080 *p*2 = 0.0248 *	5.23 ± 0.31 *p*1 = 0.0029 *p*2 = 0.1373	4.16 ± 0.03 *p*1 = 0.0000001 *p*2 = 0.0459 *	3.66 ± 0.10 *p*1 = 0.0001 *p*2 = 0.0298 *	3.48 ± 0.03 *p*1 = 0.00000004 *p*2 = 0.0039 *	3.32 ± 0.02 *p*1 = 0.00000003 *p*2 = 0.0001 *	3.25 ± 0.02 *p*1 = 0.00000005 *p*2 = 0.0001 *
Screening of the reference drug—pentoxifylline (*n* = 6)								
Initial	5.94 ± 0.59	4.90 ± 0.43	4.10 ± 0.38	3.87 ± 0.34	3.40 ± 0.29	2.69 ± 0.26	2.32 ± 0.12	2.21 ± 0.12
After incubation in control	7.53 ± 0.45 *p*1 = 0.0519	6.36 ± 0.40 *p*1 = 0.0364	5.79 ± 0.44 *p*1 = 0.0250	5.19 ± 0.31 *p*1 = 0.0184	4.37 ± 0.13 *p*1 = 0.0026	3.56 ± 0.15 *p*1 = 0.0065	2.76 ± 0.09 *p*1 = 0.0098	2.53 ± 0.07 *p*1 = 0.0218
After incubation with pentoxifylline	7.03 ± 0.43 *p*1 = 0.1584 *p*2 = 0.4306	5.81 ± 0.30 *p*1 = 0.1009 *p*2 = 0.2800	5.00 ± 0.21 *p*1 = 0.0357 *p*2 = 0.1205	4.56 ± 0.16 *p*1 = 0.0532 *p*2 = 0.0855	4.05 ± 0.10 *p*1 = 0.0171 *p*2 = 0.0631	3.24 ± 0.14 *p*1 = 0.0563 *p*2 = 0.1353	2.56 ± 0.08 *p*1 = 0.0960 *p*2 = 0.0999	2.39 ± 0.07 *p*1 = 0.1887 *p*2 = 0.1590

*n* is the number of animals in the group; *p* is the significance level; *p*1 < 0.05 are statistically significant differences compared to the initial values; *p*2 < 0.05 are statistically significant differences compared to the corresponding values in the control samples (marked *).

**Table 7:** Redocking validation metrics for the native co-crystallized ligands of MMP-9 targets.

PDB ID	Native Ligand	Heavy-Atom RMSD, Å	Centroid Displacement, Å	Validation Status
**6ESM**	B9Z	1.988	0.381	PASS
**8K5Y**	VP6	1.540	0.800	PASS
**4XCT**	N73	2.052	0.181	Marginally Passed (RMSD ≈ 2.0 Å) *

* For 4XCT/N73, although the heavy-atom RMSD slightly exceeds the strict 2.0 Å threshold by 0.052 Å, the minimal centroid displacement (0.181 Å) and preservation of key binding pocket interactions confirm successful spatial repositioning within the catalytic site.

## Data Availability

The data that support the findings of this study are available within the article and the Supplementary Materials. Further data are available from the corresponding author upon reasonable request.

## Supplementary Materials

The following supporting information can be downloaded at https://www.mdpi.com/article/10.3390/molecules31162842/s1. Section S1: Chemical Synthesis and Analytical Data: Table S1: Chemical structures and systematic IUPAC names of compounds **3**, **5**, and **6**; Experimental General Information: Instrumentation, reagents, and analytical conditions; Experimental Procedures: Detailed synthetic protocols and characterization data; Figure S1: Gas-chromatography (GC) chromatogram of the crude reaction mixture; Figure S2: Mass spectrum of compound **5** corresponding to the chromatographic peak at t_R_ = 15.39 min; Figure S3: Copies of 1H and 13C NMR spectra for the synthesized products. Section S2: Crystallographic and Quantum-Chemical (DFT) Data: Table S2: Comparison of selected experimental (XRD) and calculated (DFT) bond lengths (Å) for compound **5** in the gas phase and water; Table S3: Comparison of selected experimental (XRD) and calculated (DFT) valence angles (°) for compound **5** in the gas phase and water; Table S4: Comparison of selected experimental (XRD) and calculated (DFT) torsion angles (°) for compound **5** in the gas phase and water; Table S5: Accuracy assessment and statistical evaluation of the utilized DFT functionals in replicating the XRD experimental geometric parameters of compound **5**. Table S6 Atomic Cartesian coordinates of optimized geometry of **5a**; Table S7: Atomic Cartesian coordinates of optimized geometry of **5b**. Section S3: Biological and Molecular Docking In Silico Screening: Table S8: Predicted biological targets, interaction profiles, and associated functional parameters for compound **5** determined via inverse virtual screening; Table S9: Comprehensive matrix of intermolecular interaction types and specific amino acid residues involved in the binding of the investigated ligands within the human matrix metalloproteinase-9 (MMP-9) catalytic pocket; Figure S4: 3D and 2D spatial visualizations of ligand–amino acid interaction topologies for compound **5** and reference standards within the active sites of diverse human MMP-9 crystallographic domains.
