# Supplementary material for: Unexpected Synthesis of a Furoxan Derivative from 3-Acetyl-2,4,6-Trimethylpyridine: Structural Characterization and Biological Evaluation
Source: Molecules. 2026 Aug 14;31(16):2842. doi: 10.3390/molecules31162842 (PMC13515417; doi:10.3390/molecules31162842)
Supplement: Supplementary file 1 [file molecules-31-02842-s001.zip › molecules-4463795-supplementary.pdf]

SUPPLEMENTARY MATERIALS

Unexpected Synthesis of a Furoxan Derivative from 3-Acetyl-2,4,6-trimethylpyridine: Structural Characterization and Biological Evaluation

Aida S. Rakhimzhanova , Irina A. Pustolaikina[1*], Alfiya F. Kurmanova[1], Ruslan A. Muzaparov[1], Tatyana V. Rybalova[2], Zarina T. Shulgau[3], Alena L. Stalinskaya [4] and Ivan V. Kulakov[1,4]

[1] Department of Physical and Analytical Chemistry, Buketov Karaganda National Research University, Karaganda 100024, Kazakhstan; aida_ekb@mail.ru (A.S.R.), pustolaikina.i@karnu-buketov.edu.kz (I.A.P.); alfiya_kurmanova@mail.ru (A.F.K.), mega.muzaparov@mail.ru (R.A.M.)

[2] N. N. Vorozhtsov Novosibirsk Institute of Organic Chemistry of the Siberian Branch of Russian Academy of Sciences, Novosibirsk 630090, Russia; rybalova@nioch.nsc.ru (T.V.R.)

[3] Department of Science Development, Astana Medical University, Astana 010000, Kazakhstan; shulgau.z@amu.kz (Z.T.S.)

[4] Higher School of Natural Science, University of Tyumen, Tyumen 625003, Russia; a.l.stalinskaya@utmn.ru (A.L.S.), i.v.kulakov@utmn.ru (I.V.K.)

* Correspondence: pustolaikina.i@karnu-buketov.edu.kz (I.A.P.)

List of Content

**Section S1: Chemical Synthesis and Analytical Data**	**S2**
Table **S1** Compounds **3**,**5**,**6** and their IUPAC names	**S2**
Experimental General Information	**S2**
Experimental Procedures	**S2**
Figure **S1** Chromatogram of the reaction mixture	**S3**
Figure **S2** Mass spectrum of the compound **5** with t_R = 15,39 min of the reaction mixture	**S3**
Figure **S3** Copies of NMR Spectra of Products	**S5**
**Section 2: Crystallographic and Quantum-Chemical (DFT) Data**	**S6**
Table **S2** Selected Experimental (XRD) and Calculated (DFT) Bond Lengths (d, Å) for Compound 5 in Gas Phase and Water	**S6**
Table **S3** Selected Experimental (XRD) and Calculated (DFT) Valence Bond Angles (ω, °) for Compound **5** in Gas Phase and Water	**S9**
Table **S4** Selected Experimental (XRD) and Calculated (DFT) Torsion Angles (τ, °) for Compound **5** in Gas Phase and Water	**S10**
Table **S5** Accuracy Assessment of DFT Functionals in Replicating XRD Experimental Geometric Parameters of Compound 5	**S13**
Table **S6** Atomic cartesian coordinates of optimized geometry of **5a**	**S13**
Table **S7** Atomic cartesian coordinates of optimized geometry of **5b**	**S14**
**Section 3: Biological and Molecular Docking In Silico Screening**	**S15**
Table **S8** Predicted biological targets, interaction profiles, and associated functional parameters for the investigated compound 3,4-bis(2,4,6-trimethylnicotinoyl)-1,2,5-oxadiazol-2-N-oxide *5*	**S15**
Table **S9** Types of intermolecular interactions and specific amino acid residues involved in the binding of tested ligands within the MMP-9 receptor site	**S18**
Figure **S4** 3D- and 2-D visualizitaion of ligand – amino acids interactions of Compound 5 and Reference Standards within the Active Site of Matrix Metalloproteinase-9 (MMP-9)	**S20**

Section S1: Chemical Synthesis and Analytical Data

Table S1 Chemical structures and systematic IUPAC names of compounds **3**, **5**, and **6**; Experimental General Information: Instrumentation, reagents, and analytical conditions; Experimental Procedures: De-tailed synthetic protocols and characterization data

№	Compound	IUPAC names
**3**	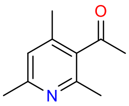	1-(2,4,6-trimethylpyridin-3-yl)ethan-1-one
**5**	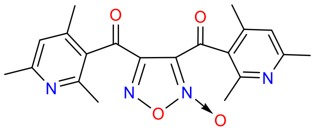	3,4-bis(2,4,6-trimethylnicotinoyl)-1,2,5-oxadiazole-2-N-oxide
**6**	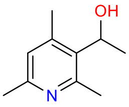	1-(2,4,6-trimethylpyridin-3-yl)ethan-1-ol

Experimental

General Information

^{1}H and ^{13}C NMR spectra were recorded on a Jeol JNM-ECA 400 spectrometr (400 and 101 MHz, respectively) and Bruker AVANCE 500 (500 and 126 MHz, respectively) instruments using $CDCl_3$ the internal standard was TMS or residual solvent signals (7.26 and 77.0 ppm for ^{1}H and ^{13}C nuclei in $CDCl_3$).

Chromato-mass spectrometric studies were carried out on a Trace GC Ultra chromatograph with a DSQ II mass-selective detector in the electron ionization mode (70 eV) on a Thermo TR-5 MS quartz capillary column, 15 m long, 0.25 mm inner diameter, with a film thickness of the stationary phase of 0.25 μm. Splitless input mode was used. Carrier gas discharge 20 ml/min. The velocity of the carrier gas (helium) is 1 ml/min. Evaporator temperature 200°C, transition chamber temperature 200°C, ion source temperature 200°C. The temperature of the column thermostat was changed according to the program: from 15 (5 min delay) to 220°C at a rate of 20°C per minute, to 290° at a rate of 15° per minute. The total analysis time was 30 min. The volume of the injected sample is 1 μl. Chromatograms were recorded in TIC mode. The range of mass scanning is 30 - 450 amu.

Melting points were determined using a Stuart SMP10 hot bench. Monitoring of the reaction course and the purity of the products was carried out by TLC on Sorbfil plates and visualized using iodine vapor or UV light.

Experimental Procedures

1-(2,4,6-trimethylpyridin-3-yl)ethan-1-one **3** was synthesized according to published procedures [Dornow A., Machens H. Uber einige Analoga des Heterovitamins B1. Chemische Berichte, 1947, Vol. 80 (6), 502-505. https://doi.org/10.1002/cber.19470800605].

3,4-bis(2,4,6-trimethylnicotinoyl)-1,2,5-oxadiazole-2-*N*-oxide **5**. A solution of 1.63 g (10.0 mmol) of 3-acetyl-2,4,6-trimethylpyridine **3** in 2 ml of AcOH was added dropwise to a nitrating mixture of 5.1 g (50.0 mmol) KNO_3 and 2.7 ml (50.0 mmol) H_2SO_4 with stirring in an ice bath. At the end of the addition, the pyridine mixture was stirred at 60-70 °C for 24 h. Upon completion of the addition (TLC and GC-MS monitoring), the mixture was poured onto ice water, neutralized with potash, and extracted with EtOAc (3 x 20 ml). The combined organic layers were washed with saturated NaCl solution and dried over anhydrous Na_2SO_4. After evaporation of the solvent under vacuum, the oil was added and purified by recrystallization from hexane:methylene chloride (4:1) mixture. Yield: 250 mg (12%), white crystals,mp 95-97 °C. ^{1}H NMR (400 MHz, $CDCl_3$) δ ppm 2.23 (s, 3H, 2-CH_3), 2.28 (s, 3H, 2′-CH_3), 2.42 (s, 3H, 6-CH_3), 2.44 (s, 3H, 6′-CH_3), 2.49, 2.50 (2 s, 6H, 4,4′-CH_3), 6.92 (s, 2H, H-6,6′ Py). ^{13}C NMR (101 MHz, $CDCl_3$) δ ppm 19.2 (CH_3), 19.6 (CH_3), 22.9 (CH_3), 23.6 (CH_3), 24.6 (CH_3), 112.0, 122.9, 123.0, 128.8, 129.3, 146.2, 146.4, 154.5, 154.8, 155.1, 160.6, 160.7, 183.7 (C=O), 187.1 (C=O). MS (EI) m/z (I_{rel}, %): $[M]^+$ 364.07 (6.5), 200.03 (9.5), 148.08 (100), 120.05 (67.8). Anal. calcd for $C_{20}H_{20}N_4O_4$: C, 63.35; H, 5.15; N, 14.90; found: C, 63.15; H, 5.30; N, 14.73.

Synthetic method for 1-(2,4,6-trimethylpyridin-3-yl)ethan-1-ol **6** was previously described by our group of researches [Shulgau, Z., Stalinskaya, A., Sergazy, S., Zhulikeyeva, A., Kamyshanskiy, Y., Gulyayev, A., Ramankulov, Y., Kulakov, I. Synthesis, hemorheological and antifibrotic activity of newly synthesized 3-acetyl-2, 4, 6-trimethylpyridine

derivatives // Arabian Journal of Chemistry. –2023. – Vol. 16. – Iss. 7. – P. 104821]. To a solution of 3-acetyl-2,4,6-trimethylpyridine 3 (2.0 g, 12.0 mmol) in 8 ml of EtOH-water (5:3) was added with stirring in portions $NaBH_4$ (0.9 g, 24.0 mmol). Stirring was maintained for 2 hours after $NaBH_4$ was all added, then water (50 ml) was added, and the mixture extracted with EtOAc (3 x 20 ml). The combined organic layers were washed with saturated NaCl solution and dried over anhydrous Na_2SO_4. The solvent was evaporated under reduced pressure, and the residue was purified by recrystallization from hexane. Yield: 1.8 g (90%), white crystals, mp 94-95°C. ^{1}H NMR (500 MHz, $CDCl_3$) δ ppm 1.48 (d, J = 6.8 Hz, 3H, -$CHCH_3$), 2.38 (2 s, 6H, 4-CH_3, 6-CH_3), 2.48 (s, 3H, 2-CH_3), 5.28 (q, J = 6.7 Hz, 1H, -CH), 6.73 (s, 1H, H-5). ^{13}C NMR (126 MHz, $CDCl_3$) δ ppm 20.1, 21.8, 23.2, 23.7, 66.3, 124.3, 133.6, 145.7, 154.9, 155.5. MS (EI) m/z (I_{rel}, %): $[M]^+$ 165.00 (20), 150.04 (100), 122.11 (44). Anal. calcd for $C_{10}H_{15}NO$: C, 72.53; H, 9.37; N, 8.65; found: C, 72.69; H, 9.15; N, 8.48.

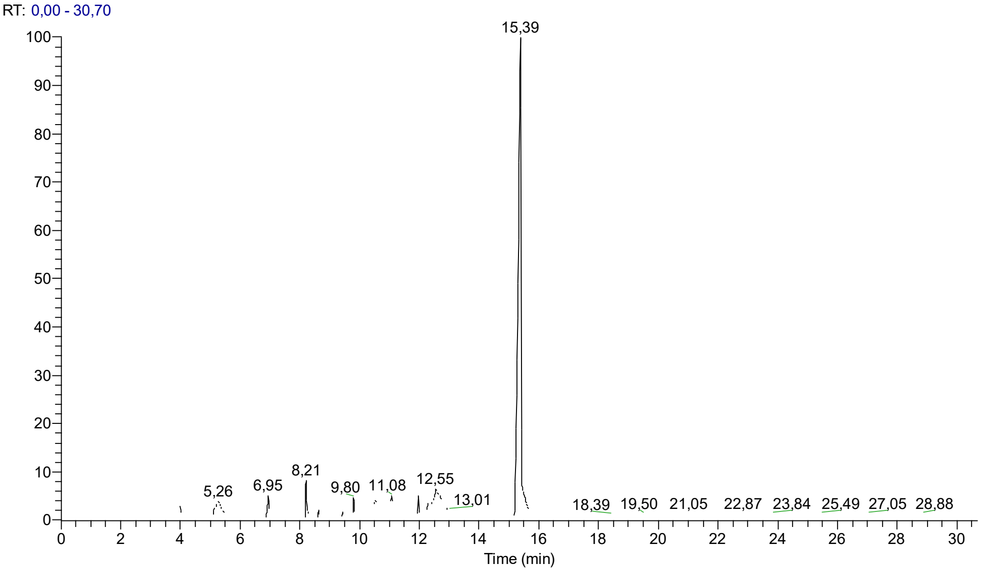

Figure S1. Gas-chromatography (GC) chromatogram of the crude reaction mixture

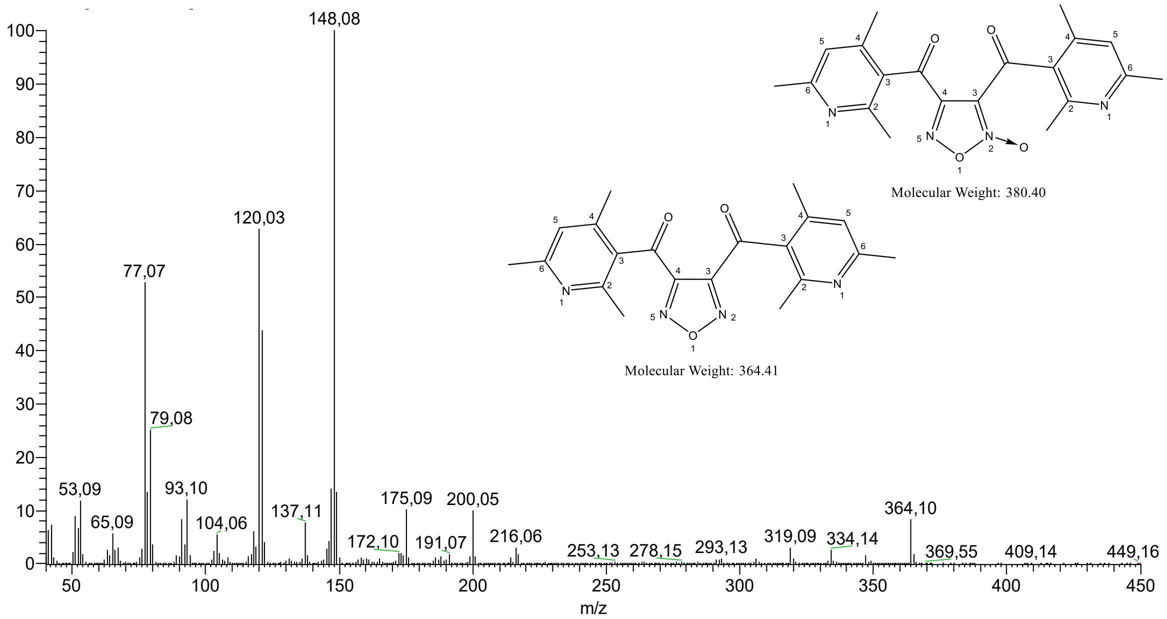

Figure S2. Mass spectrum of compound **5** corresponding to the chromatographic peak at t_R = 15.39 min

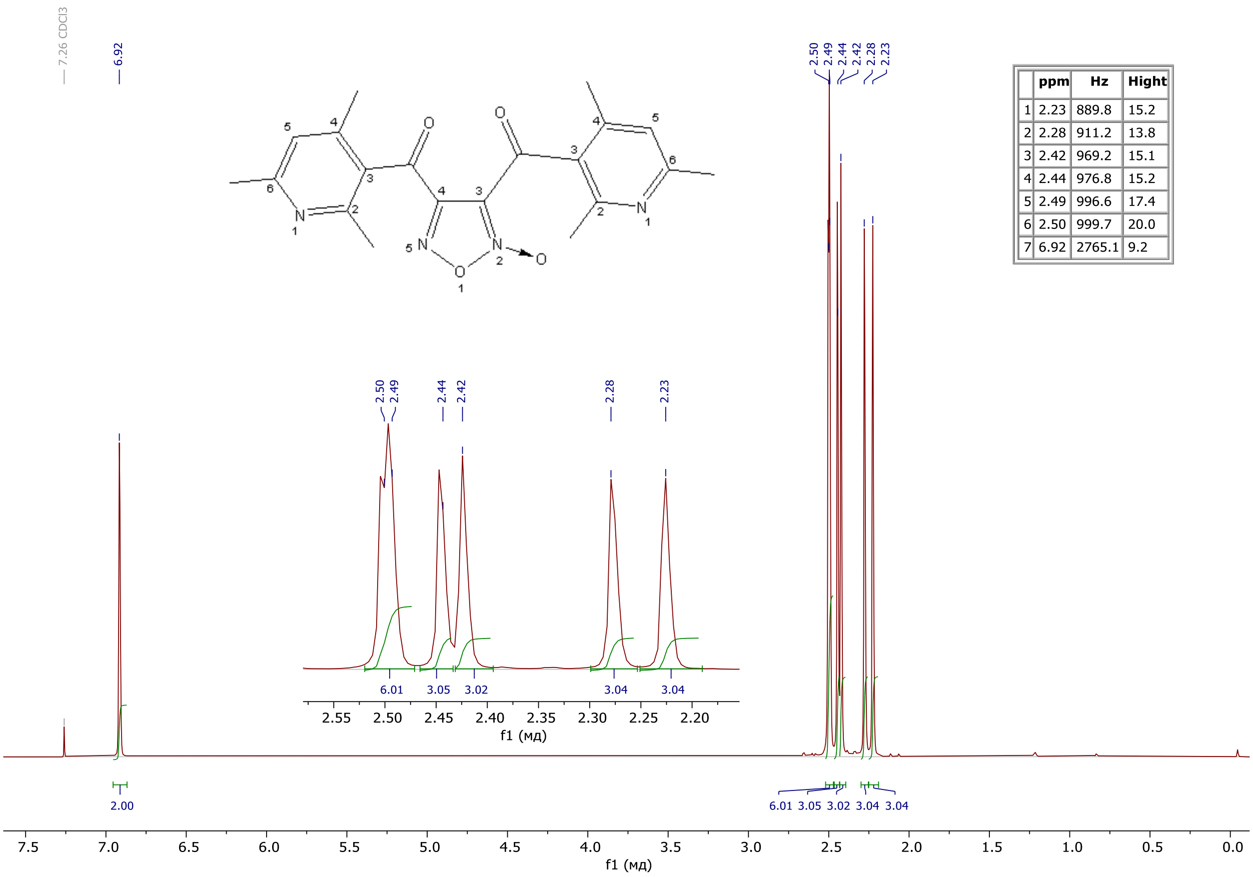

	ppm	Hz	Hight
1	2.23	889.8	15.2
2	2.28	911.2	13.8
3	2.42	969.2	15.1
4	2.44	976.8	15.2
5	2.49	996.6	17.4
6	2.50	999.7	20.0
7	6.92	2765.1	9.2

13C NMR (101 MHz, CHLOROFORM - *D*) δ 187.1, 183.7, 160.7, 160.6, 155.1, 154.8, 154.5, 146.4, 146.2, 129.3, 128.8, 123.0, 122.9, 112.0, 24.6, 23.6, 22.9, 19.6, 19.2.

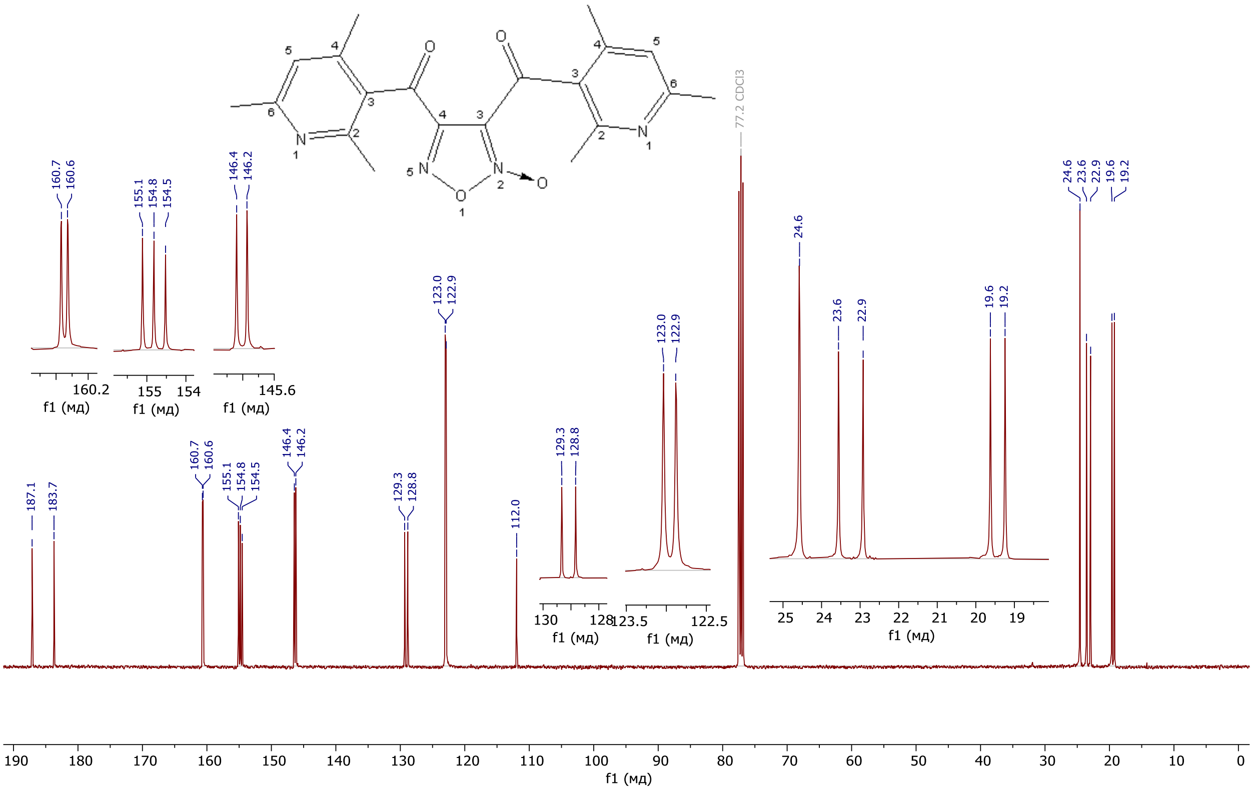

^{1}H (400 MHz, $CDCl_3$) and ^{13}C (101 MHz, $CDCl_3$) NMR Spectra of **5**

	ppm	Hz	Hight
1	1.47	737.3	9472.7
2	1.49	744.1	9388.2
3	2.37	1185.4	17609.0
4	2.38	1191.1	16653.6
5	2.48	1238.0	16359.4
6	5.26	2628.9	679.8
7	5.27	2635.6	2236.3
8	5.28	2642.3	2227.5
9	5.30	2649.1	661.3
10	6.73	3368.0	4466.1

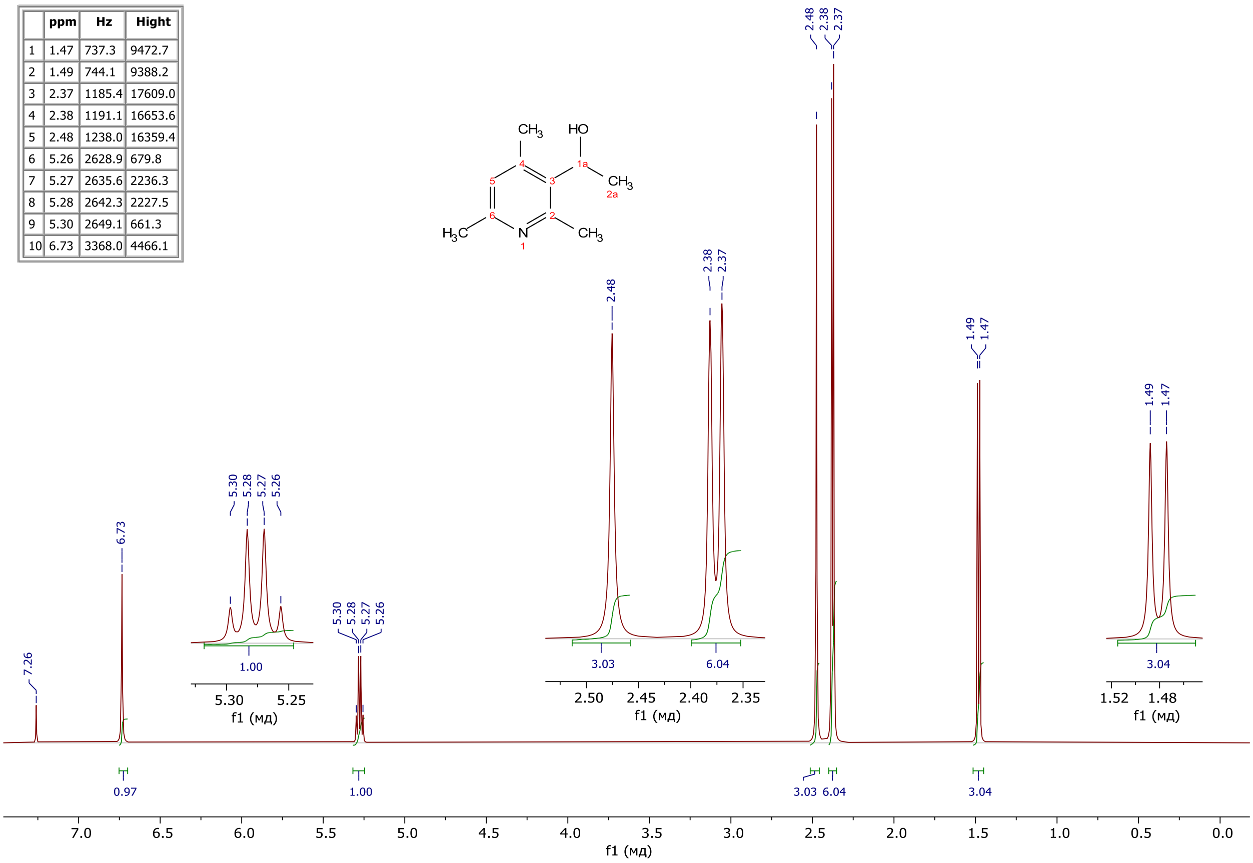

13C NMR (126 MHz, $CDCl_3$) δ 155.5, 154.9, 145.7, 133.6, 124.3, 66.3, 23.7, 23.2, 21.8, 20.1.

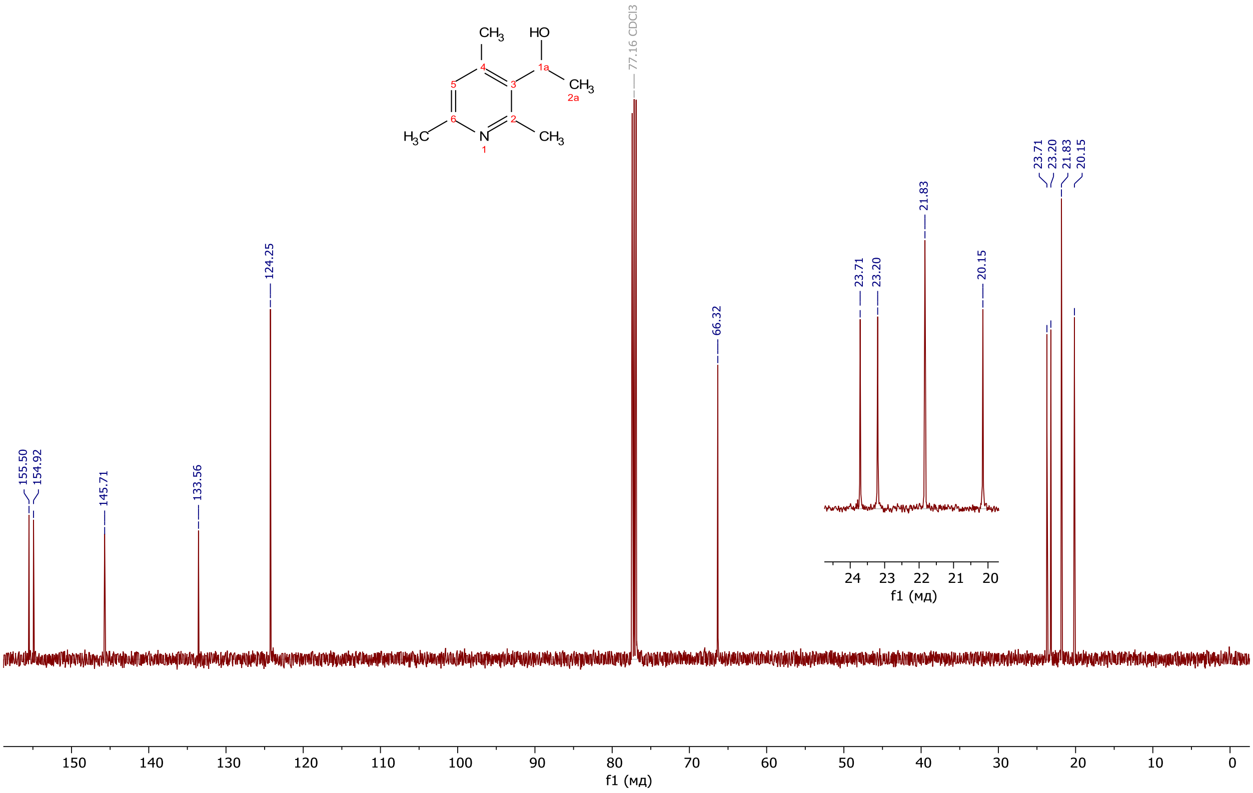

^{1}H (500 MHz, $CDCl_3$) and ^{13}C (126 MHz, $CDCl_3$) NMR Spectra of **6**

Figure S3. Copies of 1H and 13C NMR spectra for the synthesized products

Section S2: Crystallographic and Quantum-Chemical (DFT) Data

Table S2. Comparison of selected experimental (XRD) and calculated (DFT) bond lengths (Å) for compound **5** in the gas phase and water

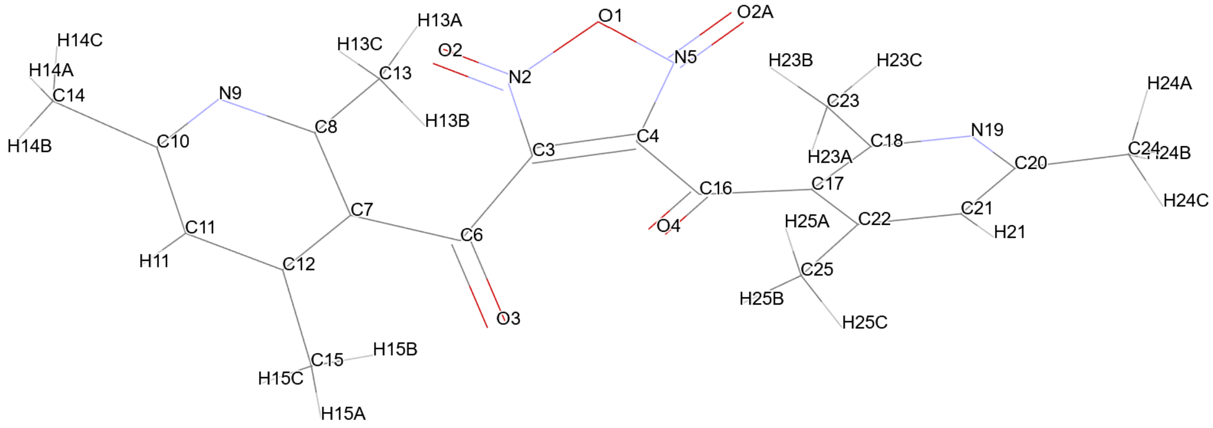

No	Atom 1	Atom 2	d, Å XRD (Exp.)	Structural Group Type	d, Å (vacuum)			d, Å (CPCM, water)		
					M062X	wB97XD	B3LYP-D3	M062X	wB97XD	B3LYP-D3
Central Oxadiazole (Furoxan) and *N*-Oxide Fragments										
1	O1	N2	1.446(2)	Endocyclic	1.40017	1.42530	1.49229	1.35399	1.35076	1.35794
2	O1	N5	1.365(4)	Endocyclic	1.35597	1.35143	1.35512	1.39315	1.41536	1.47316
3	N2	O2	1.172(3)	Exocyclic (major N–O)	1.20784	1.20654	1.20814	***	***	***
4	N2	C3	1.317(3)	Endocyclic	1.32390	1.32744	1.32913	1.29198	1.29740	1.30204
5	N5	O2A	1.10(1)	Exocyclic (minor disordered N–O)	***	***	***	1.21121	1.20934	1.21071
6	C4	N5	1.300(4)	Endocyclic	1.29227	1.29791	1.30268	1.32393	1.32845	1.33195
7	C3	C4	1.417(3)	Endocyclic	1.41917	1.41835	1.42369	1.42114	1.42042	1.42594
Carbonyl Linkers										
8	C3	C6	1.501(3)	Exocyclic	1.48830	1.49089	1.49332	1.50533	1.50591	1.50758
9	C6	O3	1.210(3)	Exocyclic (C=O)	1.20911	1.21107	1.21853	1.20564	1.20887	1.21645
10	C4	C16	1.495(3)	Exocyclic	1.50563	1.50567	1.50608	1.48676	1.48835	1.48980
11	C16	O4	1.207(3)	Exocyclic (C=O)	1.20427	1.20777	1.21544	1.20936	1.21153	1.21939
First Nicotinoyl Fragment (C7–C12, N9 ring)										
12	C6	C7	1.485(3)	Exocyclic	1.47961	1.48132	1.47899	1.47916	1.48083	1.47907
13	C7	C8	1.401(3)	Endocyclic	1.40489	1.40630	1.41446	1.40442	1.40659	1.41484
14	C7	C12	1.397(2)	Endocyclic	1.40125	1.40350	1.41104	1.40242	1.40504	1.41237
15	C8	N9	1.344(3)	Endocyclic	1.32736	1.33023	1.33403	1.32906	1.33180	1.33575
16	N9	C10	1.347(3)	Endocyclic	1.33317	1.33486	1.33971	1.33606	1.33831	1.34313
17	C10	C11	1.379(4)	Endocyclic	1.38861	1.39065	1.39503	1.38902	1.39036	1.39459
18	C11	C12	1.373(4)	Endocyclic	1.38616	1.38786	1.39157	1.38659	1.38886	1.39227
19	C8	C13	1.501(3)	Exocyclic (methyl)	1.50536	1.50736	1.51072	1.50574	1.50702	1.51080
20	C10	C14	1.510(5)	Exocyclic (methyl)	1.49858	1.50082	1.50370	1.49807	1.50030	1.50311
21	C12	C15	1.504(4)	Exocyclic (methyl)	1.50173	1.50375	1.50657	1.50075	1.50288	1.50578

Second Nicotinoyl Fragment (C17–C22, N19 ring)										
22	C16	C17	1.490(3)	Exocyclic	1.47977	1.48110	1.47978	1.48103	1.48357	1.48170
23	C17	C18	1.400(3)	Endocyclic	1.40789	1.40946	1.41748	1.40534	1.40592	1.41469
24	C17	C22	1.384(4)	Endocyclic	1.39899	1.40216	1.40894	1.39902	1.40116	1.40802
25	C18	N19	1.339(4)	Endocyclic	1.32763	1.33006	1.33397	1.32962	1.33208	1.33578
26	N19	C20	1.335(6)	Endocyclic	1.33415	1.33616	1.34114	1.33630	1.33898	1.34380
27	C20	C21	1.379(4)	Endocyclic	1.38796	1.38945	1.39374	1.38835	1.38972	1.39379
28	C21	C22	1.367(4)	Endocyclic	1.38688	1.38904	1.39296	1.38664	1.38909	1.39268
29	C18	C23	1.488(5)	Exocyclic (methyl)	1.50190	1.50393	1.50698	1.50303	1.50482	1.50797
30	C20	C24	1.515(6)	Exocyclic (methyl)	1.49866	1.50084	1.50384	1.49823	1.50061	1.50326
31	C22	C25	1.507(4)	Exocyclic (methyl)	1.50534	1.50706	1.51052	1.50345	1.50563	1.50863
C–H Bonds (Geometrically Constrained)										
32	C11	H11	0.93	Aromatic CH	1.08246	1.08421	1.08393	1.08213	1.08373	1.08339
33	C21	H21	0.93	Aromatic CH	1.08260	1.08434	1.08408	1.08222	1.08383	1.08346
34	C13, 14, 15	H	0.96	Methyl groups (C13, C14, C15)	1.08715/ 1.08819/ 1.08829	1.08934/ 1.09101/ 1.09067	1.08963/ 1.09118/ 1.09090	1.08747/ 1.08759/ 1.08776	1.08956/ 1.09027/ 1.09008	1.08989/ 1.09041/ 1.09030
35	C23, 24, 25	H	0.96	Methyl groups (C23, C24, C25)	1.08651/ 1.08821/ 1.08864	1.08878/ 1.09105/ 1.09092	1.08892/ 1.09125/ 1.09126	1.08684/ 1.08766/ 1.08804	1.08912/ 1.09031/ 1.09017	1.08931/ 1.09048/ 1.09047

Table S3. Comparison of selected experimental (XRD) and calculated (DFT) valence angles (°) for compound 5 in the gas phase and water

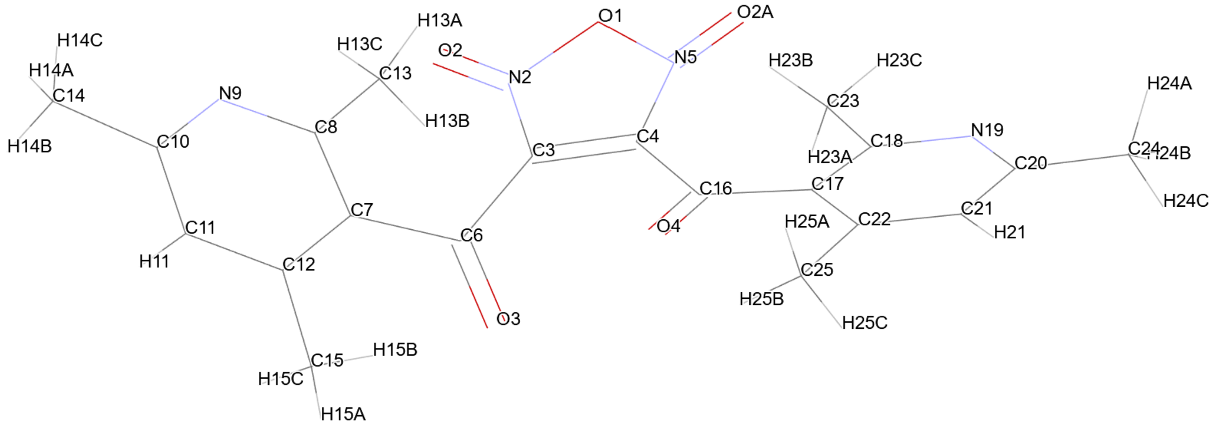

No	Ato m 1	Atom 2 (top)	Atom 3	Bond angle (ω, °)	ω, °(vacuum)			ω, ° (CPCM, water)		
					M062X	wB97XD	B3LYP-D3	M062X	wB97XD	B3LYP-D3
Central Oxadiazole (Furoxan) and *N*-Oxide Fragments										
1	N2	O1	N5	107.4(2)	108.75942	108.63154	107.69876	108.79102	108.69523	107.86365
2	O1	N2	O2	114.2(2)	117.55125	117.77619	118.10708	***	***	***
3	O1	N2	C3	107.0(2)	107.56543	106.85307	105.41118	107.11156	107.30374	107.80397
4	O2	N2	C3	138.7(2)	134.88240	135.36128	136.47510	***	***	***
5	N2	C3	C4	106.9(2)	105.64322	106.07797	107.24990	110.84663	111.02718	111.58270
6	C3	C4	N5	111.0(2)	111.01350	111.17684	111.71786	105.40606	105.76858	106.73908
7	O1	N5	C4	107.7(2)	107.01718	107.25777	107.91953	107.84436	107.20431	106.00984
Disordered position:										
8	O1	N5	O2A	97.8(6)	***	***	***	117.18216	117.37676	117.63606
9	O2A	N5	C4	152.8(6)	***	***	***	134.97305	135.41371	136.35129
**Conjugation angles of bridging carbonyl groups**										
10	N2	C3	C6	121.5(2)	125.17869	124.45796	124.19953	120.79259	120.51697	120.71553
11	C4	C3	C6	131.1(2)	128.56805	128.91969	127.81446	127.90290	127.92107	127.14475
12	O3	C6	C3	117.4(2)	116.37395	116.92581	116.41648	117.27761	117.45326	117.07923
13	O3	C6	C7	125.8(2)	123.85051	124.13018	124.32324	124.69818	124.73390	124.71503
14	C3	C6	C7	116.7(2)	119.73040	118.91781	119.20847	117.87386	117.68960	118.06047
15	N5	C4	C16	118.9(2)	121.57349	121.39487	121.83011	125.15216	124.60021	124.44895
16	C3	C4	C16	129.7(2)	126.74877	126.70844	125.60967	128.84278	129.10643	128.04486
17	O4	C16	C4	118.3(2)	116.56120	116.78393	116.33421	116.70680	117.26286	116.71008
18	O4	C16	C17	124.7(2)	124.93483	124.89603	124.81649	123.75390	123.84307	123.85669
19	C4	C16	C17	116.8(2)	118.37154	118.22339	118.73700	119.50942	118.88304	119.38595
First Nicotinoyl Fragment (C7–C12, N9 ring)										
20	C6	C7	C8	120.3(2)	121.39208	121.00222	121.35915	120.73104	120.56791	121.03367
21	C6	C7	C12	120.4(2)	119.20478	119.61672	119.49351	119.67438	119.92738	119.73192
22	C8	C7	C12	119.2(2)	119.38495	119.36661	119.13020	119.58673	119.49430	119.22270
23	C7	C8	N9	121.8(2)	121.80141	121.88037	121.85971	121.69215	121.79949	121.78787
24	C7	C8	C13	122.4(2)	123.10820	123.07802	123.23533	122.76000	122.77184	122.98532
25	N9	C8	C13	115.8(2)	115.06273	115.02146	114.87325	115.50940	115.40261	115.18426
26	C8	N9	C10	118.1(2)	119.44844	119.34144	119.50681	119.43170	119.34688	119.50784
27	N9	C10	C11	122.5(2)	122.01095	122.06333	121.93154	121.98362	121.99955	121.86674
28	N9	C10	C14	116.0(2)	116.23623	116.20869	116.23718	116.45626	116.44534	116.46684
29	C11	C10	C14	121.5(3)	121.75202	121.72767	121.83074	121.55892	121.55457	121.66541
30	C10	C11	C12	120.2(2)	120.20821	120.22478	120.34645	120.28022	120.33379	120.44829
31	C7	C12	C11	117.7(2)	117.08662	117.07741	117.14483	116.97105	116.98069	117.08967

32	C7	C12	C15	123.0(2)	123.09366	122.89671	122.96969	123.06716	123.10507	123.15169
33	C11	C12	C15	119.2(2)	119.81454	120.01723	119.87687	119.95398	119.90520	119.74723
Second Nicotinoyl Fragment (C17–C22, N19 ring)										
34	C16	C17	C18	119.3(2)	119.39496	119.61115	119.43935	119.02461	119.43697	119.06797
35	C16	C17	C22	121.3(2)	121.12282	121.00532	121.37874	121.30222	120.86727	121.52517
36	C18	C17	C22	119.3(2)	119.47428	119.37260	119.16990	119.66246	119.68607	119.39879
37	C17	C18	N19	121.3(3)	121.59206	121.74755	121.77877	121.49179	121.58691	121.63329
38	C17	C18	C23	122.9(3)	122.53926	122.59934	122.61652	122.54288	122.39550	122.57191
39	N19	C18	C23	115.7(3)	115.86370	115.64561	115.59721	115.95913	116.01035	115.78563
40	C18	N19	C20	118.8(3)	119.52221	119.41407	119.50623	119.54795	119.42229	119.54660
41	N19	C20	C21	122.4(3)	122.08625	122.10360	121.98995	121.97127	122.01236	121.87091
42	N19	C20	C24	118.0(3)	116.18794	116.15584	116.17284	116.48013	116.44800	116.46356
43	C21	C20	C24	119.6(3)	121.72503	121.73973	121.83557	121.54791	121.53904	121.66458
44	C20	C21	C22	119.9(3)	120.10630	120.17400	120.33437	120.16759	120.18959	120.36191
45	C17	C22	C21	118.3(2)	117.20218	117.17511	117.20240	117.14650	117.09256	117.17620
46	C17	C22	C25	123.2(2)	123.28314	123.32123	123.45162	123.31958	123.14889	123.43149
47	C21	C22	C25	118.5(2)	119.47747	119.47921	119.30802	119.51836	119.75031	119.37899
**Geometrically fixed angles with hydrogen atoms (C–H, methyls)**										
48	C10 / C20	C11 / C21	H11 / H21	~120.0	119.88422/119.94640	119.86749/ 119.92844	119.82187/ 119.85522	119.80761/119.89394	119.78830/ 119.89706	119.73522/ 119.81911
49	H11 / H21	C11 / C21	C12 / C22	~120.1	119.90738/119.94428	119.90723/ 119.89552	119.83034/ 119.80641	119.91161/119.93643	119.87738/ 119.91208	119.81475/ 119.81596
50	C	C (Methyl)	H (angles in methyl groups C13,14,15,23,24,25)	~109.5	108.07167/ 109.59418/ 110.05467/ 110.32568/ 109.61199/ 109.82601	110.34694/ 109.73300/ 110.32564/ 110.45191/ 109.77040/ 110.04151	110.41474/ 109.81318/ 110.49105/ 108.71828/ 109.85179/ 110.11562	108.55279/ 109.77726/ 110.05169/ 108.77223/ 109.78944/ 109.90121	108.73945/ 109.93350/ 110.26110/ 109.01582/ 109.97871/ 110.16304	108.74093/ 110.00885/ 110.40628/ 109.07976/ 110.02423/ 110.22748
51	H	C (Methyl)	H (tetrahedral angles inside methyls)	~109.5	109.16433/ 109.25610/ 108.94259/ 109.27092/ 109.29121/ 108.26560	109.03221/ 109.02938/ 108.84008/ 109.56000/ 109.04274/ 108.20523	109.06576/ 108.97256/ 108.91807/ 109.74011/ 108.99135/ 108.05396	108.68129/ 109.08653/ 109.02540/ 109.50522/ 109.06330/ 108.41798	108.55792/ 108.85386/ 108.67152/ 109.12219/ 108.85638/ 108.11848	108.58207/ 108.82513/ 108.79228/ 109.34382/ 108.80013/ 108.09676

Table S4. Comparison of selected experimental (XRD) and calculated (DFT) torsion angles (°) for compound **5** in the gas phase and water

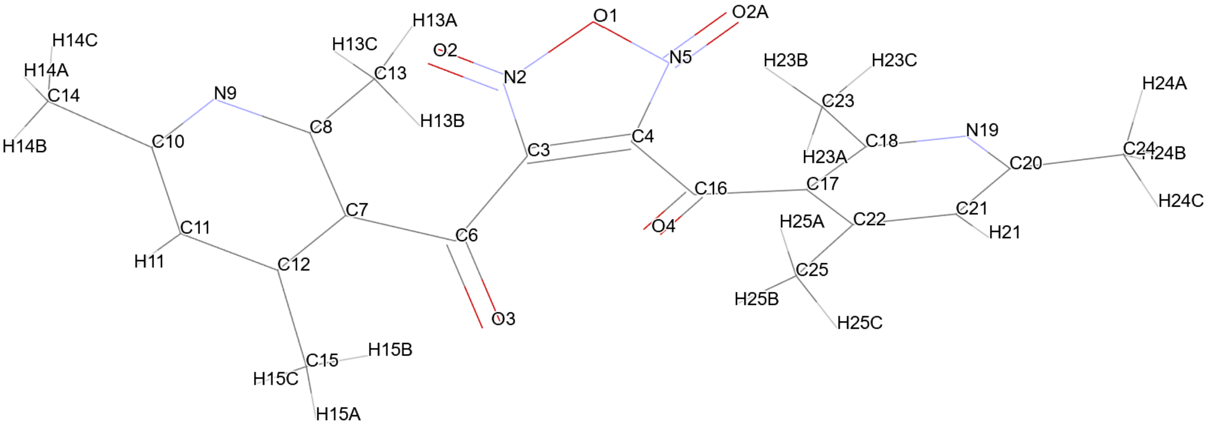

No	Atom 1	Atom 2	Atom 3	Atom 4	Torsion Angle (τ, °)	Conformational Fragment Description	τ, ° (vacuum)			τ, ° (CPCM, water)		
							M062X	wB97XD	B3LYP-D3	M062X	wB97XD	B3LYP-D3
**Planarity of the Central Heterocycle (1,2,5-Oxadiazole / Furoxan Ring)**												
1	N5	O1	N2	C3	0.2(2)	Furoxan ring geometry (nearly perfectly planar)	-0.33727	-0.31661	-0.48573	0.05383	0.21241	0.07034
2	N2	O1	N5	C4	-0.0(3)	Furoxan ring geometry	0.35671	0.52132	0.53210	0.07465	-0.02522	0.11201
3	O1	N2	C3	C4	-0.4(2)	Furoxan ring geometry	0.18079	0.00000	0.24716	-0.15631	-0.31545	-0.22192
4	N2	C3	C4	N5	0.4(3)	Furoxan ring geometry	0.03854	0.33476	0.07315	0.20290	0.30072	0.29707
5	C3	C4	N5	O1	-0.2(3)	Furoxan ring geometry	-0.24681	-0.53198	-0.39348	-0.15963	-0.15481	-0.23216
**Orientation of the N-Oxide Group (Major and Minor Positions)**												
6	N5	O1	N2	O2	-179.3(2)	Coplanarity of the major O2 atom with the ring	179.95970	-179.36812	-179.70141	***	***	***
7	O2	N2	C3	C4	178.9(3)	Orientation of the major O2 oxygen atom	179.80920	178.80575	179.24256	***	***	***
8	N2	O1	N5	O2A	170.5(6)	Deviation of the minor O2A atom from the ring plane	***	***	***	-179.72212	-179.32150	-179.37705
9	C3	C4	N5	O2A	-159(1)	Orientation of the minor disordered O2A oxygen	***	***	***	179.58482	178.95499	179.11205
10	C16	C4	N5	O2A	28(1)	Spatial arrangement of O2A relative to carbonyl C16	***	***	***	7.81003	6.62141	8.46060
**Twisting of Carbonyl Linkages Relative to the Central Heterocycle**												
11	O1	N2	C3	C6	-173.2(2)	Rotation of the C6=O3 group relative to furoxan	-171.49649	-172.18205	-170.58110	-172.98150	-172.57007	-172.25251
12	O2	N2	C3	C6	6.1(4)	Relative rotation of major O2 and C6=O3 group	8.13192	6.62355	8.41430	***	***	***
13	N2	C3	C4	C16	172.4(2)	Rotation of the C16=O4 group relative to furoxan	170.73382	170.63671	169.66261	171.56536	172.16532	170.50386

14	C6	C3	C4	N5	172.2(2)	Relative arrangement of two carbonyl substituents	171.33516	172.04638	170.46688	172.38802	171.83732	171.69722
15	C6	C3	C4	C16	-15.7(4)	Conjugation distortion between C6=O and C16=O	-17.96956	-17.65167	-19.94366	-16.24953	-16.29808	-18.09599
16	C16	C4	N5	O1	-173.3(2)	Twisting of C16 linker out of the heterocycle plane	-171.50032	-171.42889	-170.43624	-171.93442	-172.48838	-170.88361
**Mutual Rotation of Oxadiazole and Nicotinoyl Fragments**												
17	N2	C3	C6	O3	142.4(2)	Conformation of the C3–C6=O3 linkage	141.72343	137.55375	136.10897	130.51524	130.00767	128.00649
18	N2	C3	C6	C7	-34.1(3)	Dihedral angle of the first pyridine ring (C7) to furoxan	-35.92678	-40.66146	-41.38675	-45.23964	-46.14989	-47.82137
19	C4	C3	C6	O3	-28.5(4)	Spatial direction of carbonyl oxygen O3	-28.00731	-32.77512	-32.78125	-40.97804	-40.81639	-42.68570
20	C4	C3	C6	C7	155.1(2)	Deviation of C7 pyridine ring from conjugation plane	154.34248	149.00967	149.72303	143.26707	143.02604	141.48644
21	C3	C4	C16	O4	-25.1(4)	Spatial direction of carbonyl oxygen O4	-41.85342	-41.24819	-41.12486	-21.88533	-24.94674	-22.93496
22	C3	C4	C16	C17	159.6(2)	Dihedral angle of the second pyridine ring (C17) to furoxan	142.12172	142.14972	142.54123	160.02798	156.21488	159.46907
23	N5	C4	C16	O4	146.4(3)	Conformation of the C4–C16=O4 linkage	127.94237	128.14775	127.47921	147.91483	145.52990	145.67276
24	N5	C4	C16	C17	-28.8(3)	Deviation of C17 pyridine ring from conjugation plane	-48.08249	-48.45434	-48.85469	-30.17185	-33.30849	-31.92321
**Conformation of the First Nicotinoyl Ring (C7–C12, N9)**												
25	O3	C6	C7	C8	132.6(2)	Rotation of C=O linker relative to the pyridine core	137.57963	133.59363	137.86333	137.28151	134.14110	138.07113
26	O3	C6	C7	C12	-50.8(3)	Rotation of C=O linker relative to the pyridine core	-40.85926	-45.01484	-40.61748	-41.69541	-44.67919	-40.67431
27	C3	C6	C7	C8	-51.2(3)	Conjugation angle between oxadiazole and pyridine core	-44.95538	-48.32884	-44.85250	-47.30837	-50.00848	-46.44879
28	C3	C6	C7	C12	125.3(2)	Conjugation angle between oxadiazole and pyridine core	136.60573	133.06269	136.66670	133.71471	131.17124	134.80577
29	C6	C7	C8	N9	170.6(2)	Planarity of substitution in the pyridine ring	-179.89993	-179.85193	179.47208	179.42559	179.81945	179.15403
30	C12	C7	C8	N9	-6.1(3)	Pyridine ring internal planarity (all values near 0–5°)	-1.46380	-1.24002	-2.04171	-1.59660	-1.35519	-2.09425
31	C7	C8	N9	C10	0.5(3)	Pyridine ring internal planarity (all values near 0–5°)	-0.82397	-0.77712	-0.55908	-0.57389	-0.63385	-0.43279
32	C8	N9	C10	C11	4.4(4)	Pyridine ring internal planarity (all values near 0–5°)	2.11852	1.91341	2.37773	1.90946	1.77739	2.21279
33	N9	C10	C11	C12	-3.5(4)	Pyridine ring internal planarity (all values near 0–5°)	-1.09366	-1.00495	-1.55425	-1.04772	-0.90592	-1.42711

34	C10	C11	C12	C7	-2.3(3)	Pyridine ring internal planarity (all values near 0–5°)	-1.18212	-1.00606	-1.05517	-1.10605	-1.06869	-1.10096
35	C6	C7	C12	C11	-169.8(2)	Spatial alignment of the core methyl substituents	-179.14144	-179.30523	-178.73695	-178.65363	-179.03062	-178.46946
**Conformation of the Second Nicotinoyl Ring (C17–C22, N19)**												
36	O4	C16	C17	C18	-54.0(4)	Rotation of C=O linker relative to the second pyridine	-40.58541	-43.84121	-40.58540	-46.61277	-51.66752	-46.96942
37	O4	C16	C17	C22	127.4(3)	Rotation of C=O linker relative to the second pyridine	138.38398	134.94819	138.14336	132.19275	127.19599	131.99063
38	C4	C16	C17	C18	120.9(3)	Conjugation angle of oxadiazole and second pyridine	135.07680	132.46005	135.41201	131.33144	127.08924	130.44445
39	C4	C16	C17	C22	-57.7(3)	Conjugation angle of oxadiazole and second pyridine	-45.95381	-48.75056	-45.85923	-49.86304	-54.04725	-50.59550
40	C16	C17	C18	N19	-176.7(3)	Planarity of substitution in the second pyridine ring	-179.72167	179.78623	-179.82538	179.65986	179.26385	179.69717
41	C18	C17	C22	C21	-1.2(4)	Second pyridine ring internal planarity (deviations < 1.5°)	-0.23228	0.04398	-0.33814	0.18891	0.54109	0.30972
42	C17	C18	N19	C20	-0.8(5)	Planarity of the second pyridine ring	-1.32698	-1.24986	-1.43360	-1.17645	-0.98747	-1.20536
43	C18	N19	C20	C21	-1.2(5)	Planarity of the second pyridine ring	0.32065	0.51056	0.38910	0.49892	0.65141	0.67023
44	N19	C20	C21	C22	1.9(5)	Planarity of the second pyridine ring	0.72720	0.50818	0.67326	0.53211	0.29743	0.36681
45	C20	C21	C22	C17	-0.7(4)	Planarity of the second pyridine ring	-0.72586	-0.74888	-0.65269	-0.83969	-0.86746	-0.82581
**Orientation of Peripheral Methyl Groups (Constrained Geometries)**												
46	–	C8 / C18	C13 / C23	H	~ ±30...179	Conformational positions of ortho-methyl hydrogens	87.40170/ -34.68993/ -155.51678/ -94.48220/ 143.42617/ 22.59932/ 75.69594/ -43.20490/ -164.66942/ 14.53019/ 135.99471/ -105.10445	87.45502/ -34.53983/ -155.42519/ -94.15182/ 143.85333/ 22.96797/ 74.95329/ -44.35863/ -165.45613/ 13.56331/ 134.66081/ -106.02728	87.93352/ -33.97877/ -154.94284/ -94.08318/ 144.00453/ 23.04046/ 74.77476/ -43.93959/ -165.43785/ 13.58171/ 135.07998/ -106.20567	89.68300/ -31.75935/ -152.14033/ -92.53946/ 146.01820/ 25.63722/ 80.01661/ -39.54211/ -160.81788/ 18.28826/ 139.56403/ -100.87726	88.72094/ -32.69577/ -153.18344/ -93.10701/ 145.47629/ 24.98862/ 79.45591/ -40.36923/ -161.36701/ 17.66773/ 138.66550/ -101.50936	88.40137/ -33.00111/ -153.49709/ -93.93532/ 144.66220/ 24.16623/ 79.82710/ -39.56517/ -160.94029/ 17.97400/ 139.34912/ -101.25861
47	–	C10 / C20	C14 / C24	H	~ ±19...160	Conformational positions of para-methyl hydrogens	121.70538/ -120.82512/ 0.34855/ 59.49482/ -57.97468/ -179.33151/ 119.72945/ -122.82434/ -1.41799/ -60.58412/ 56.86209/ 178.26844	121.23108/ -121.07324/ 0.00799/ 59.12609/ -58.56959/ -179.79269/ 120.58340/ -121.75155/ -0.52067/ -59.73972/ 57.92533/ 179.15621	121.04891/ -121.43385/ -0.27134/ 58.82801/ -58.68923/ 179.99051/ 120.44753/ -122.05170/ -0.71268/ -60.01005/ 57.49072/ 178.82974	120.30709/ -121.85681/ -0.77219/ 58.53395/ -59.30215/ 179.61857/ 121.32667/ -120.80754/ 0.26992/ -58.96888/ 58.89691/ 179.97437	120.46154/ -121.44004/ -0.50413/ 58.82167/ -59.27675/ 179.75758/ 121.91553/ -119.93758/ 0.94405/ -58.36011/ 59.78678/ -179.33159	119.75306/ -122.34015/ -1.27978/ 58.01907/ -59.88772/ 179.07944/ 120.82890/ -121.23572/ -0.17823/ -59.52004/ 58.41534/ 179.47283

48	–	C12 / C22	C15 / C25	H	~ ±10…173	Conformational positions of ortho-methyl hydrogens	13.52967/-106.79694/134.58409/-44.56366/74.05531/-165.61808/20.65350/140.91636/-98.18087/84.10201/-36.80076/-157.06362	10.94033/-109.52202/131.53933/-47.35909/71.57955/-167.95809/20.09162/140.46940/-98.61145/83.23952/-37.67963/-158.05741	11.22143/-109.40893/132.23156/-46.67000/71.68950/-167.68013/19.41730/139.83821/-99.40627/82.89975/-37.85577/-158.27668	14.10229/-105.79518/135.29209/-43.66000/75.25273/-164.84980/20.77863/141.38713/-97.42355/84.05361/-37.13571/-157.74421	13.63551/-106.21122/134.59949/-44.27239/74.91690/-165.23637/20.71834/141.38369/-97.65881/83.41687/-37.54063/-158.20598	13.09249/-106.97217/134.32816/-44.40656/74.29311/-165.64223/21.68160/142.41637/-96.59624/84.77044/-36.21695/-156.95172

Table S5. Accuracy assessment and statistical evaluation of the utilized DFT functionals in replicating the XRD experimental geometric parameters of compound **5**

<table>
<tr><th rowspan="2">Geometry Evaluation Criterion</th><th colspan="3">(vacuum)</th><th colspan="3">(CPCM, water)</th><th rowspan="2">Accuracy Leader</th></tr>
<tr><th>M06-2X</th><th>ωB97X-D</th><th>B3LYP-D3</th><th>M06-2X</th><th>ωB97X-D</th><th>B3LYP-D3</th></tr>
<tr><td rowspan="2">Integral core accuracy (Bond lengths and valence angles)</td><td>~97.5%</td><td>>98.5%</td><td>~94.0%</td><td>~91.5%</td><td>~91.0%</td><td>~89.5%</td><td>ωB97X-D (vacuum)</td></tr>
<tr><td colspan="7">Description ωB97X-D provides an accurate description of the central heterocycle. M06-2X somewhat underestimates the O–N bond lengths. B3LYP-D3 systematically overestimates the heteroatom bond lengths (up to +0.05 Å). In water, the accuracy of the structure description decreases due to the inversion of bond lengths in the core during stabilization of the minor state.</td></tr>
<tr><td rowspan="2">Conformational planarity (Ring and N-oxide flatness)</td><td>~98.5%</td><td>>99.0%</td><td>~97.5%</td><td>~99.0%</td><td>>99.5%</td><td>~99.0%</td><td>ωB97X-D (water)</td></tr>
<tr><td colspan="7">DescriptionIn vacuum, ωB97X-D reproduces well the coplanarity of the main O2 atom. In water, the planarity of the furoxan ring in all functionals approaches >99.5% convergence, accurately reproducing the ideal planar distribution of the electron density of the minor form</td></tr>
<tr><td rowspan="2">Conformational twist (Free rotation of pyridine rings)</td><td>~93.0%</td><td>~90.0%</td><td>~87.5%</td><td>~92.5%</td><td>>95.5%</td><td>~94.0%</td><td>ωB97X-D (water)</td></tr>
<tr><td colspan="7">Description In vacuum, the M06-2X method more accurately determines the first wing of collidine, but all methods tend to achieve a symmetric vacuum minimum for the second wing. Taking into account the polar solvent, the ωB97X-D method (water) achieves a precision of >95.5%, completely reconstructing the asymmetric rotation of the second wing observed in the crystal lattice</td></tr>
</table>

Table S6 Atomic Cartesian coordinates of optimized geometry of **5a**

```
---------------------------------------------------------------------
 Center     Atomic      Atomic             Coordinates (Angstroms)
 Number     Number       Type             X           Y           Z
 ---------------------------------------------------------------------
      1          8           0        0.065061   -0.332547    2.822928
      2          7           0       -1.041428    0.059962    2.014778
      3          8           0       -2.080369    0.318415    2.571134
      4          8           0       -0.814605    1.240109   -1.217884
      5          6           0       -0.617431    0.062768    0.756876
      6          8           0        1.124036   -1.327829   -1.253672
      7          6           0        0.745404   -0.327691    0.800852
      8          7           0        1.126864   -0.562227    2.019067
```

9	6	0	-1.408334	0.574384	-0.398749
10	6	0	-2.858911	0.280506	-0.460196
11	6	0	-3.343232	-1.023382	-0.252852
12	7	0	-4.641661	-1.307418	-0.306869
13	6	0	-5.515885	-0.328254	-0.549384
14	6	0	-5.110999	0.980657	-0.787561
15	1	0	-5.851131	1.745917	-0.992753
16	6	0	-3.763449	1.311430	-0.758211
17	6	0	-2.442689	-2.203772	0.007584
18	1	0	-2.233517	-2.294071	1.078572
19	1	0	-1.494617	-2.145839	-0.530616
20	1	0	-2.969408	-3.107372	-0.296948
21	6	0	-6.964738	-0.719742	-0.555522
22	1	0	-7.137040	-1.492210	-1.308638
23	1	0	-7.614757	0.131380	-0.763764
24	1	0	-7.236002	-1.145304	0.413582
25	6	0	-3.330886	2.728418	-1.015707
26	1	0	-2.826812	2.806428	-1.980642
27	1	0	-2.625278	3.076857	-0.258155
28	1	0	-4.194668	3.394332	-1.014715
29	6	0	1.622950	-0.666452	-0.374820
30	6	0	3.040114	-0.237581	-0.337629
31	6	0	4.055019	-1.157277	-0.670338
32	7	0	5.340618	-0.816606	-0.655177
33	6	0	5.683928	0.435846	-0.340811
34	6	0	4.738802	1.401226	-0.016245
35	1	0	5.055677	2.411628	0.217151
36	6	0	3.387929	1.078236	-0.000430
37	6	0	3.764420	-2.585402	-1.041580
38	1	0	3.356823	-2.641342	-2.053185
39	1	0	3.030216	-3.041282	-0.373970
40	1	0	4.694744	-3.149493	-0.999912
41	6	0	7.152322	0.745258	-0.365140
42	1	0	7.677592	0.100926	0.343931
43	1	0	7.352511	1.787652	-0.112680
44	1	0	7.558908	0.534294	-1.357013
45	6	0	2.374622	2.143118	0.331915
46	1	0	2.073307	2.070486	1.380973
47	1	0	1.477303	2.069623	-0.286784
48	1	0	2.808678	3.132470	0.180618

Method: DFT ωB97XD /6-311++G(d,p)
Key word: OPT FREQ
E_{total} = -1293.853140 a.u.

Table S7 Atomic Cartesian coordinates of optimized geometry of **5b**

Center Number	Atomic Number	Atomic Type	Coordinates (Angstroms) X	Y	Z
1	8	0	-0.094967	0.394003	2.820905
2	7	0	-1.155311	0.620869	2.013102
3	8	0	2.055537	-0.237993	2.576189
4	8	0	-1.148605	1.443455	-1.229058
5	6	0	-0.768398	0.389278	0.796511
6	8	0	0.796837	-1.105978	-1.255971
7	6	0	0.596666	0.004792	0.756145
8	7	0	1.015870	0.009206	2.016423

9	6	0	-1.642125	0.740287	-0.378638
10	6	0	-3.051524	0.289663	-0.369308
11	6	0	-3.387635	-1.031427	-0.017220
12	7	0	-4.648702	-1.454625	0.004814
13	6	0	-5.629599	-0.603799	-0.305795
14	6	0	-5.374870	0.707440	-0.692359
15	1	0	-6.198414	1.363275	-0.951520
16	6	0	-4.071032	1.181286	-0.743649
17	6	0	-2.357297	-2.080265	0.313711
18	1	0	-2.097712	-2.037019	1.375738
19	1	0	-1.442917	-1.974201	-0.274012
20	1	0	-2.792778	-3.059400	0.118129
21	6	0	-7.027081	-1.144360	-0.221559
22	1	0	-7.133800	-2.001255	-0.890869
23	1	0	-7.770850	-0.391456	-0.486471
24	1	0	-7.225444	-1.498918	0.792725
25	6	0	-3.806716	2.600171	-1.164875
26	1	0	-3.335130	2.626076	-2.148838
27	1	0	-3.126822	3.104788	-0.475133
28	1	0	-4.740489	3.162371	-1.206017
29	6	0	1.393231	-0.487902	-0.403464
30	6	0	2.850929	-0.219030	-0.435669
31	6	0	3.743582	-1.272680	-0.710118
32	7	0	5.058390	-1.081128	-0.761129
33	6	0	5.549673	0.146217	-0.567196
34	6	0	4.729728	1.236186	-0.302768
35	1	0	5.165027	2.219639	-0.164615
36	6	0	3.353014	1.070929	-0.222245
37	6	0	3.279142	-2.684836	-0.940370
38	1	0	2.821228	-2.781353	-1.927034
39	1	0	2.532102	-2.994221	-0.205809
40	1	0	4.141160	-3.347131	-0.880279
41	6	0	7.041617	0.283649	-0.656194
42	1	0	7.516209	-0.346764	0.099624
43	1	0	7.364188	1.315611	-0.509817
44	1	0	7.388494	-0.063450	-1.632283
45	6	0	2.477364	2.265625	0.056246
46	1	0	2.226934	2.317517	1.120528
47	1	0	1.546649	2.245684	-0.514357
48	1	0	3.008209	3.184718	-0.195794

Method: DFT ωB97XD /6-311++G(d,p)

Key word: OPT FREQ

E_{total} = -1293.853200 a.u.

Section S3: Biological and Molecular Docking In Silico Screening

Table S8. Predicted biological targets, interaction profiles, and associated functional parameters for compound **5** determined via inverse virtual screening

3 biological processes associated with probable targets:

- extracellular matrix organization;
- positive regulation of apoptotic process;
- metabolic process

3 protein classes of probable targets:

- enzyme;
- membrane receptor;
- transcription factor

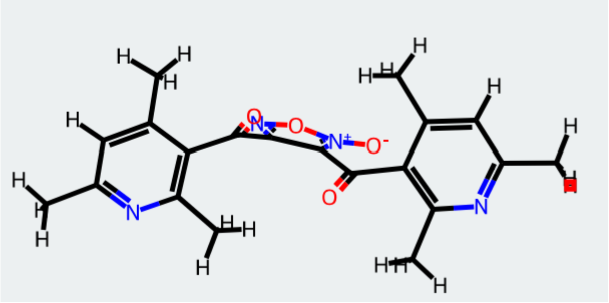

2.3

Name	Activity Score	Propensity Score	Type of Interaction	ChEMBL ID
Matrix metalloproteinase-9	0.5879	-1	I	CHEMBL321
Cyclin-dependent kinase 3	0.3943	-1	D	CHEMBL4442
Histamine H2 receptor	0.3937	-1	D	CHEMBL1941
Glutathione reductase, mitochondrial	0.3226	0.2887	D	CHEMBL2755
dCTP pyrophosphatase 1	0.2975	-1	D	CHEMBL3769292
Epidermal growth factor receptor	0.2864	-1	I	CHEMBL203_L858R
Indoleamine 2,3-dioxygenase 1	0.2242	-1	I	CHEMBL4685_S167A
Steroid hormone receptor ERR1	0.2210	-1	I	CHEMBL3429
Myosin light chain kinase, smooth muscle	0.2091	0.0640	D	CHEMBL2428
Histone acetyltransferase KAT2A	0.2047	0.0637	D	CHEMBL5501
Prostaglandin G/H synthase 1	0.2003	0.0972	D	CHEMBL221
Peptidyl-prolyl cis-trans isomerase D	0.2001	-1	D	CHEMBL1697657
cGMP-dependent protein kinase 1	0.1817	-1	D	CHEMBL4273
Tumor necrosis factor	0.1771	-1	D	CHEMBL1825
Ribosomal protein S6 kinase alpha-5	0.1770	0.1261	D	CHEMBL4237
Aryl hydrocarbon receptor	0.1755	-1	D	CHEMBL3201
Bis(5'-adenosyl)-triphosphatase	0.1684	-1	D	CHEMBL1795151
Maternal embryonic leucine zipper kinase	0.1674	-1	I	CHEMBL4578
Glucose-6-phosphate 1-dehydrogenase	0.1647	-1	I	CHEMBL5347
Transcription factor p65	0.1626	-1	D	CHEMBL5533
Muscarinic acetylcholine receptor M4	0.1574	-1	D	CHEMBL1821
Dual specificity calcium/calmodulin-dependent 3',5'-cyclic nucleotide phosphodiesterase 1C	0.1549	-1	I	CHEMBL4619
Prostaglandin G/H synthase 2	0.1504	0.1235	D	CHEMBL230
Thioredoxin reductase 1, cytoplasmic	0.1494	0.0292	I	CHEMBL1927
Sortilin	0.1398	0.0759	D	CHEMBL3091
Probable global transcription activator SNF2L2	0.1395	-1	I	CHEMBL2362979
3',5'-cyclic-AMP phosphodiesterase 7B	0.1374	0.0375	D	CHEMBL4716
Muscarinic acetylcholine receptor M5	0.1281	-1	D	CHEMBL2035
Catenin beta-1	0.1230	-1	D	CHEMBL5866
RNA demethylase ALKBH5	0.1186	0.0308	I	CHEMBL3621037
Ectonucleotide pyrophosphatase/phosphodiesterase family member 3	0.1143	-1	I	CHEMBL5580

Transient receptor potential cation channel subfamily M member 2	0.1095	-1	I	CHEMBL1250402
Carbonic anhydrase 12	0.1082	-1	D	CHEMBL3242
Pyruvate kinase PKM	0.1067	-1	I	CHEMBL1075189
MIF/CD74 (Macrophage migration inhibitory factor and HLA-DR antigens-associated invariant chain)	0.1039	-1	I	CHEMBL2111430
Fructose-1,6-bisphosphatase 1	0.1020	-1	D	CHEMBL3975
Eukaryotic elongation factor 2 kinase	0.1005	-1	D	CHEMBL5026

Table S9. Comprehensive matrix of intermolecular interaction types and specific amino acid residues involved in the binding of the investigated ligands within the human matrix metalloproteinase-9 (MMP-9) catalytic pocket

Ligand	Receptor	Conventional Hydrogen Bond	Unfavorable Donor- Donor	π-Cation	π-anion	π-Alkyl	π-Sigma	π-Sulfur	Carbon Hydrogen Bond	Unfavorable acceptor-acceptor	π-π T-shaped	Metal-Acceptor	π-π Stacked	Halogen
**5**	**6ESM**	PZE307	-	-	-	PRO193, LEU187	-	-	HIS236	-	-	Zn301	HIS230	-
B9Z	**6ESM**	LEU188	-	-	-	VAL223, LEU243	TYR248	-	ALA242, ARG249	-	-	-	HIS226	-
VP6	**6ESM**	PRO246	-	-	-	LEU188, HIS230, HIS236	-	-	LEU187	-	-	-	-	-
N73	**6ESM**	LEU188, GLN227	-	Zn301	-	VAL223, HIS236	TYR248	-	ARG301	-	-	-	HIS226	-
Doxycycline	**6ESM**	HIS190, ALA189	-	-	-	LEU188	-	-	HIS236	-	-	Zn301		-
Marimastat	**6ESM**		-	-	-	HIS230, LEU188	-	-	-	ALA189	-	Zn301		-
Captopril	**6ESM**	LEU188	-	-	-	LEU188	HIS226	TYR248	-	-	-	-	-	-
**5**	**8K5Y**	-	-	-	-	PRO193, HIS230, VAL101, PHE110	LEU114	-	-	-	PHE107	-	-	-
B9Z	**8K5Y**	LEU114	-	-	-	PRO102, VAL101, PRO193	-	-	ASP235	-	HIS190		PHE107, TYR179, PHE110	-
VP6	**8K5Y**	GLN108	-	-	-	VAL101, PHE192, TYR179	PRO193, HIS190		PHE107	-	-	-	-	ARG106
N73	**8K5Y**	-	-	-	-	LEU114, PRO193	PHE107	-	-	-	TYR179, PHE110			
Doxycycline	**8K5Y**	-	-	-	-	VAL101, LEU114, PRO193	-	-	-	-	-	-	-	-
Marimastat	**8K5Y**	GLY100, ARG106, ARG51	-	-	-	PRO102	TYR179	-	GLY178, PRO180	-	-	-	-	-
Captopril	**8K5Y**	-	-	-	-	PRO102, ARG51, PHE192	-	HIS190, TYR179	-	-	-	-	-	-
**5**	**4XCT**	-	-	Zn302	-	LEU188, VAL223, ALA189, PRO246, LEU187, HIS190, TYR179	HIS226, DMS307	-	-	-	HIS236	-	HIS236	-
VP6	**4XCT**	ARG249	TYR248	Zn302	-	LEU222,	-	MET247	HIS236	-	-	-	HIS226	-

						VAL223,<br>LEU243								
B9Z	**4XCT**	HIS190	-	-	-	LEU222,<br>VAL223,<br>LEU188	-	-	-	-	HIS236	Zn302	TYR248,<br>HIS226	-
N73	**4XCT**	LEU188	-	Zn302,<br>HIS230	GLU227	VAL223,<br>LEU187	-	-	HIS190,<br>HIS236	-	TYR248	Zn302	HIS226	
Doxycycline	**4XCT**	GLU227,<br>HIS190	-	-	-	LEU188	-	-	HIS236	-	-	Zn302	-	-
Marimastat	**4XCT**	MET247	-	-	-	LEU188	-	-	ALA189	-	-	Zn302	-	-
Captopril	**4XCT**		-	-	-	LEU188	HIS226	TYR248	LEU187	-	-	-	-	-

Figure S4. 3D and 2D spatial visualizations of ligand–amino acid interaction topologies for compound 5 and reference standards within the active sites of diverse human MMP-9 crystallographic domains.

8K5Y – 5

3D-visualizitaion	2D-visualizitaion
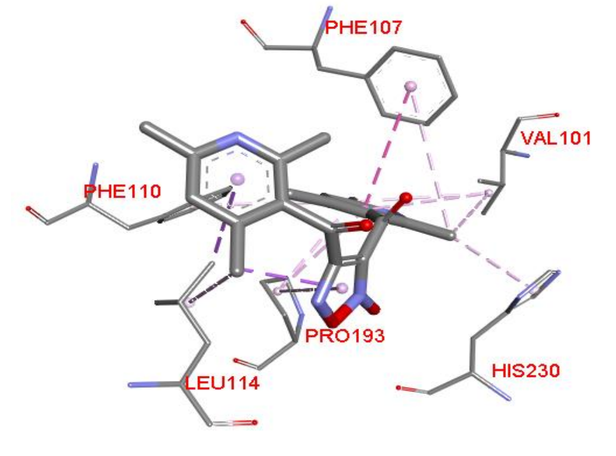	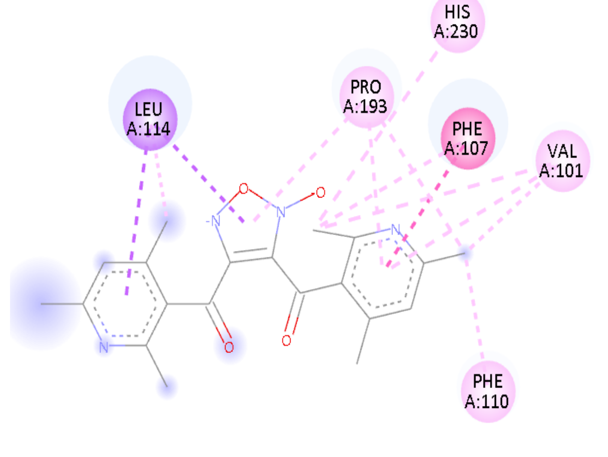

8K5Y – VP6

3D-visualizitaion	2D-visualizitaion
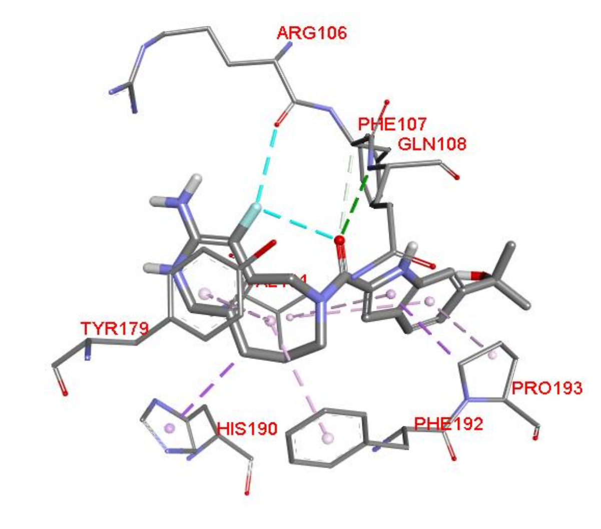	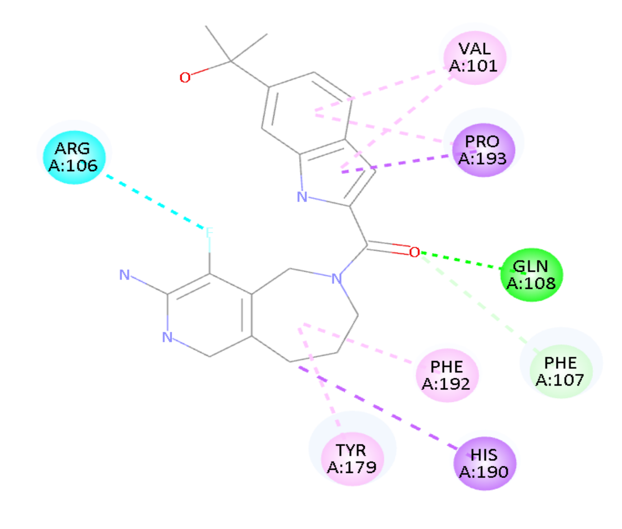

8K5Y – B9Z

3D-visualizitaion	2D-visualizitaion

<table>
<tr><td>
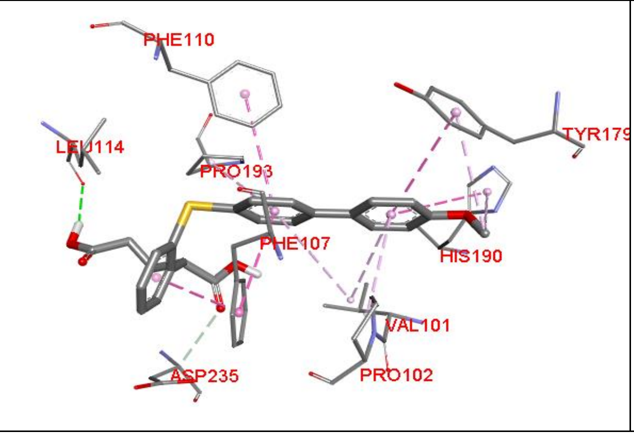

</td><td>
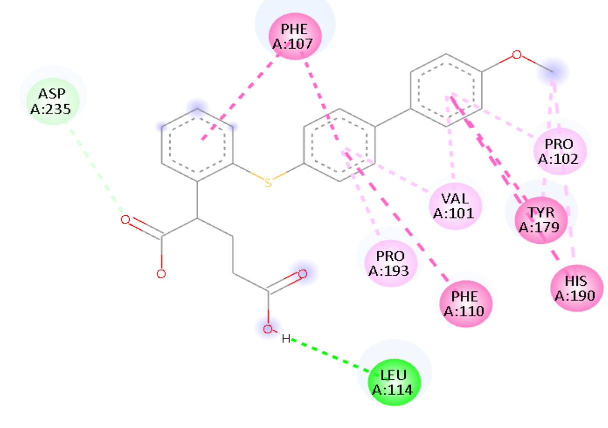

</td></tr>
</table>

8K5Y – N73

<table>
<tr><th>3D-visualizitaion</th><th>2D-visualizitaion</th></tr>
<tr><td>
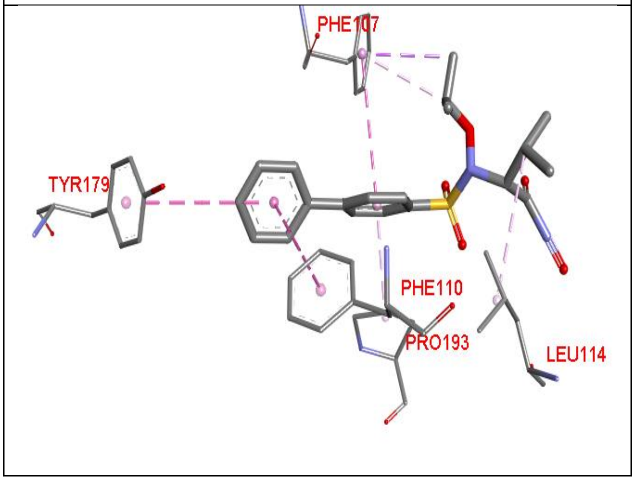

</td><td>
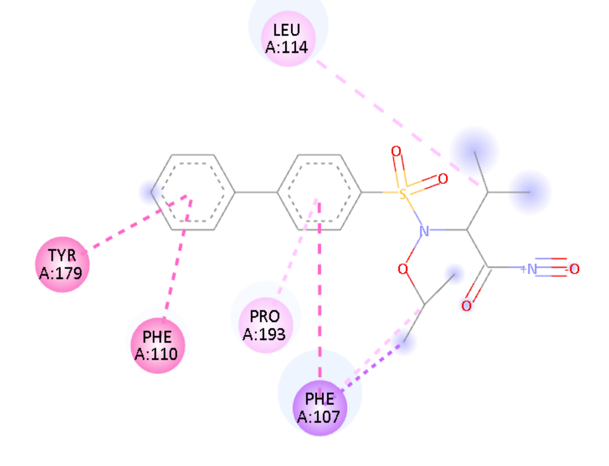

</td></tr>
</table>

8K5Y – doxycycline

<table>
<tr><th>3D-visualizitaion</th><th>2D-visualizitaion</th></tr>
<tr><td>
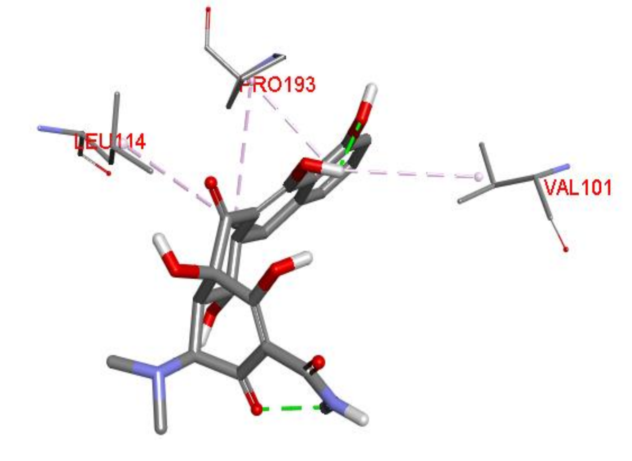

</td><td>
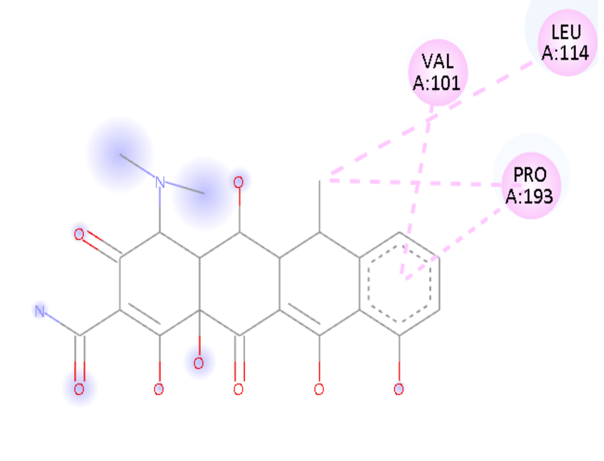

</td></tr>
</table>

8K5Y – marimastat

3D-visualizitaion	2D-visualizitaion

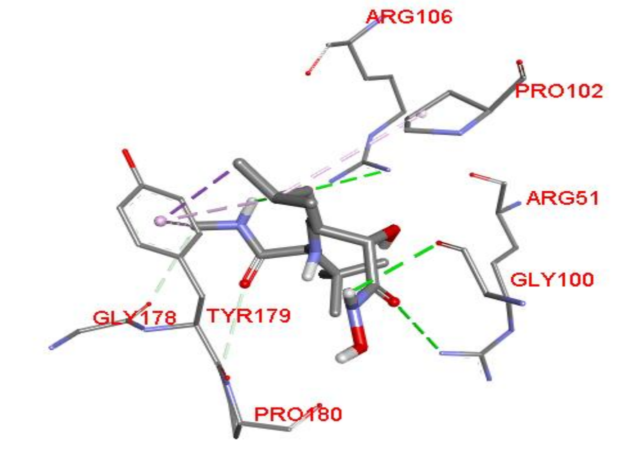	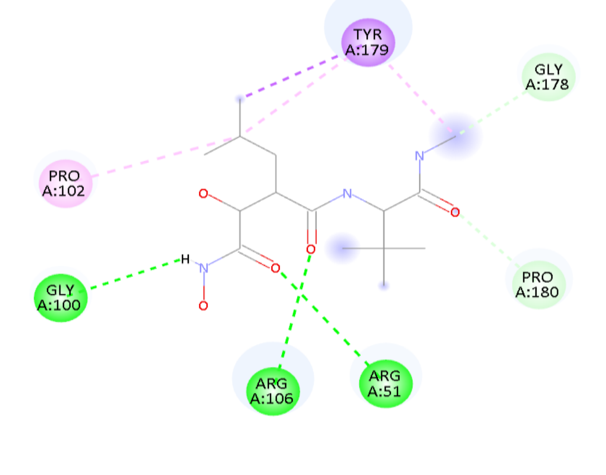

8K5Y – captopril

3D-visualizitaion	2D-visualizitaion
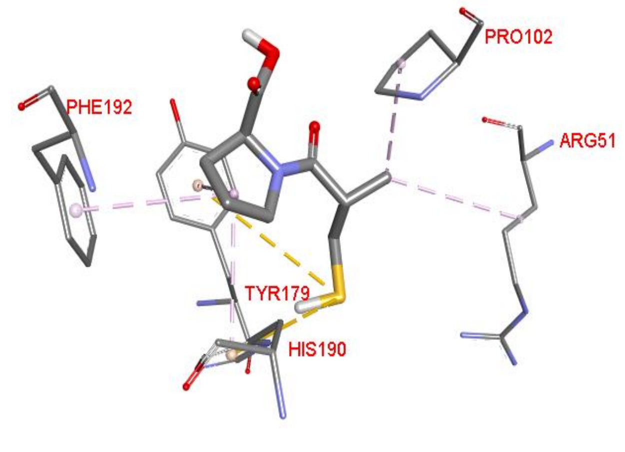	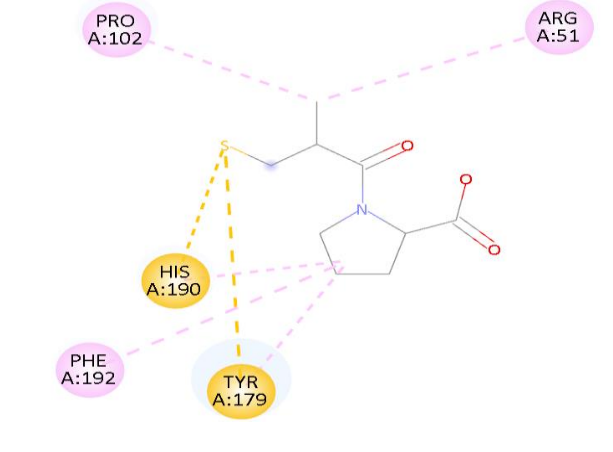

6ESM – 5

3D-visualizitaion	2D-visualizitaion
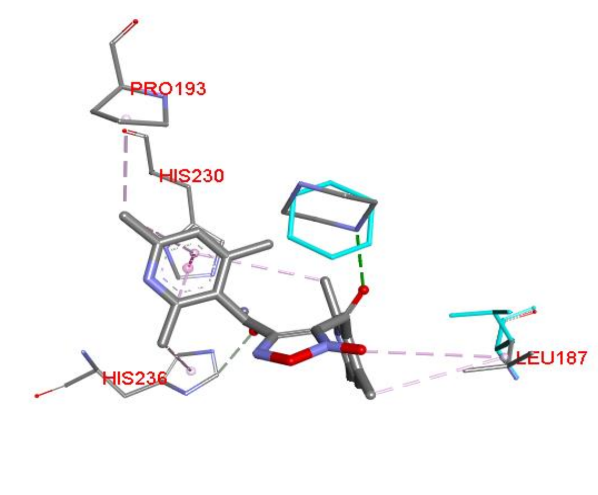	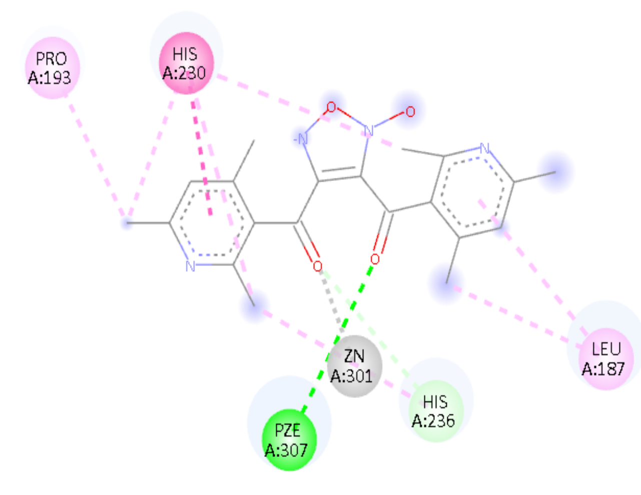

6ESM –VP6

3D-visualizitaion	2D-visualizitaion

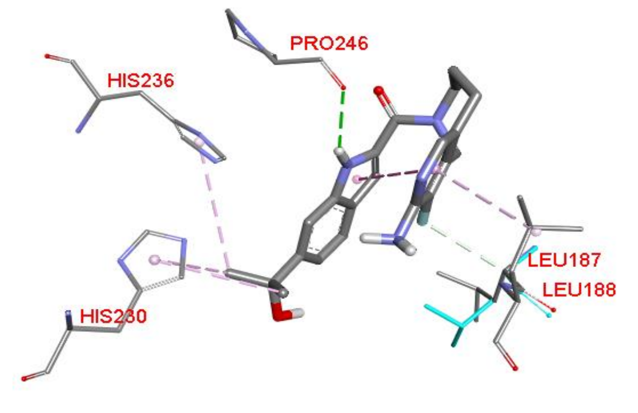	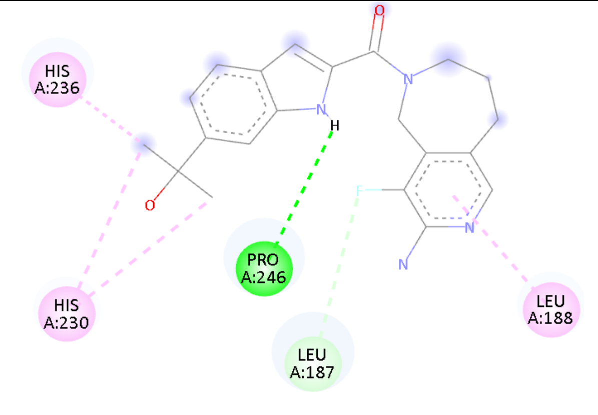

6ESM – B9Z

3D-visualizitaion	2D-visualizitaion
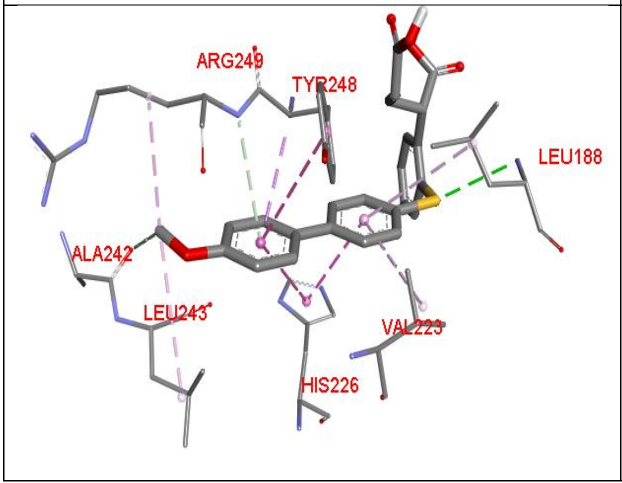	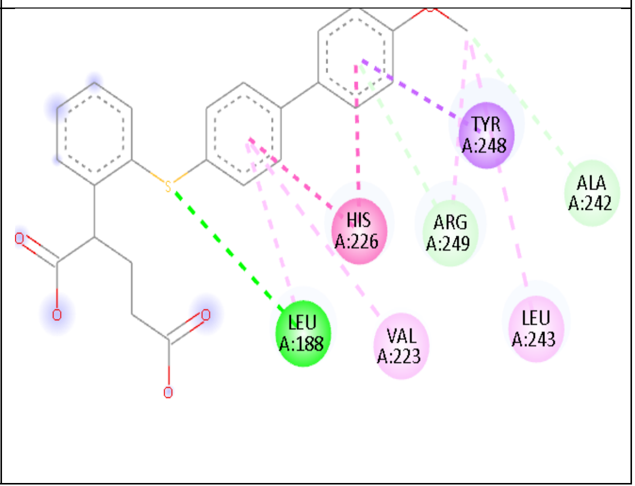

6ESM – N73

3D-visualizitaion	2D-visualizitaion
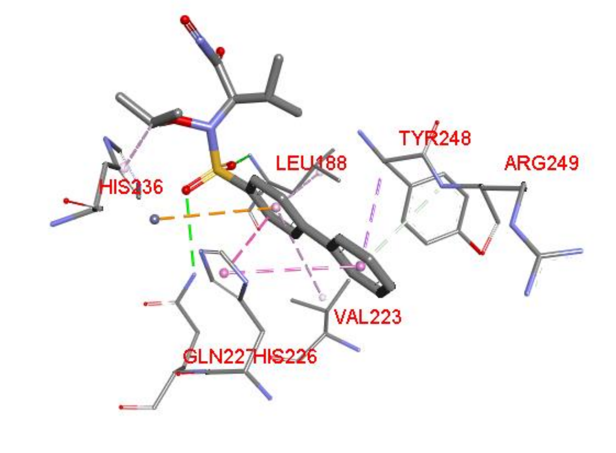	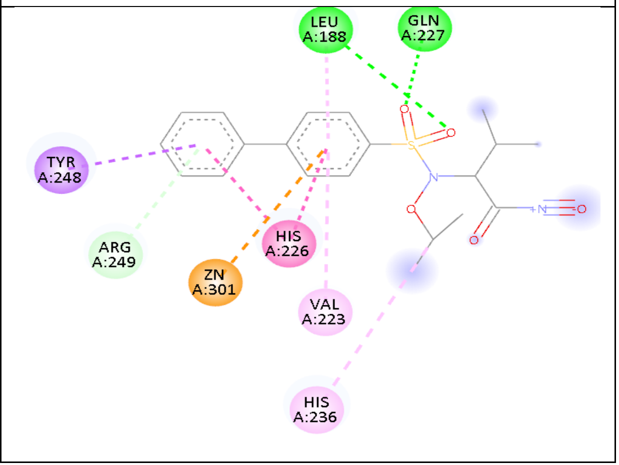

6ESM – doxycycline

3D-visualizitaion	2D-visualizitaion

<table>
<tr><td>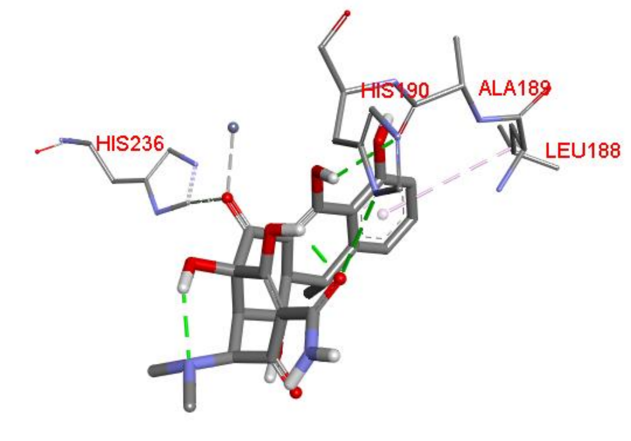
</td><td>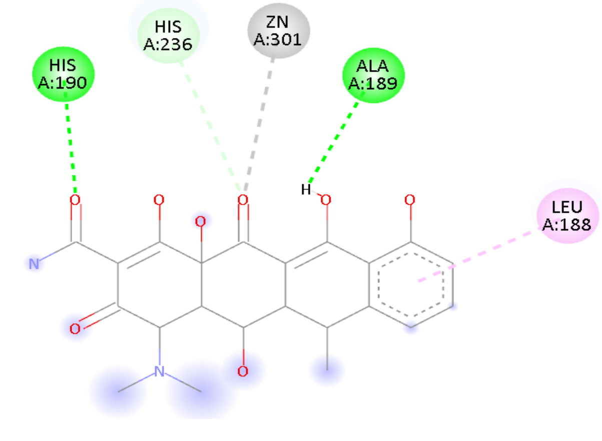
</td></tr>
</table>

6ESM – marimastat

<table>
<tr><th>3D-visualizitaion</th><th>2D-visualizitaion</th></tr>
<tr><td>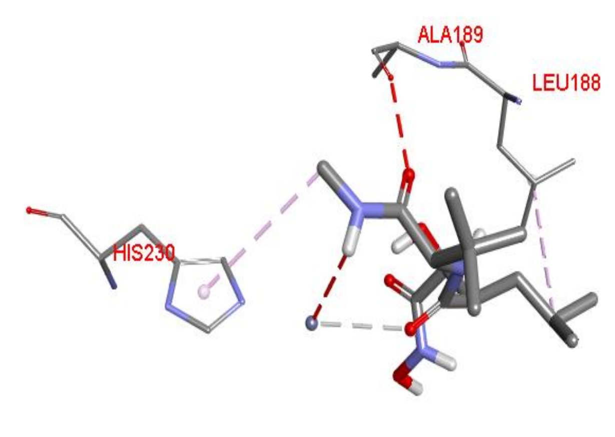
</td><td>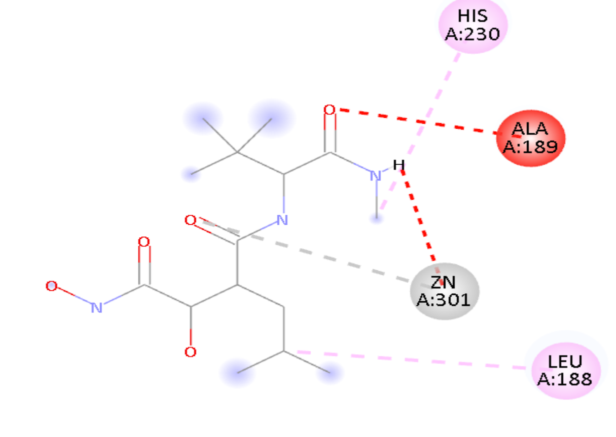
</td></tr>
</table>

6ESM – captopril

<table>
<tr><th>3D-visualizitaion</th><th>2D-visualizitaion</th></tr>
<tr><td>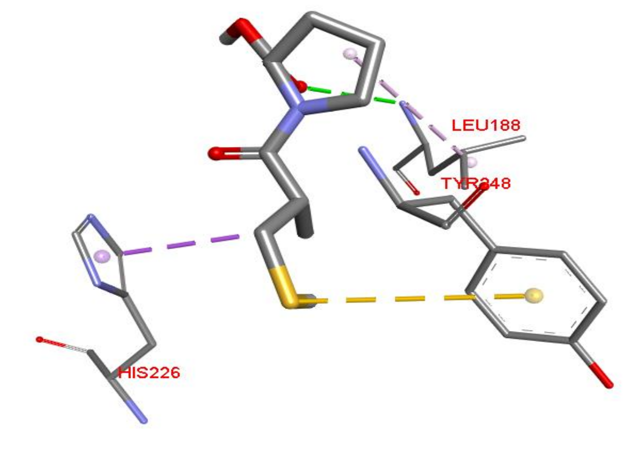
</td><td>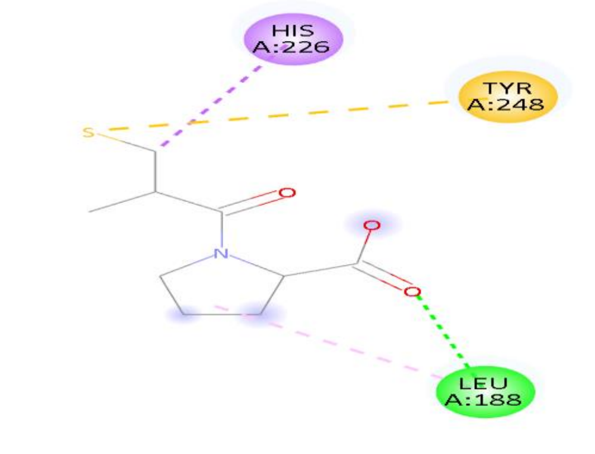
</td></tr>
</table>

4XCT – 5

3D-visualizitaion	2D-visualizitaion

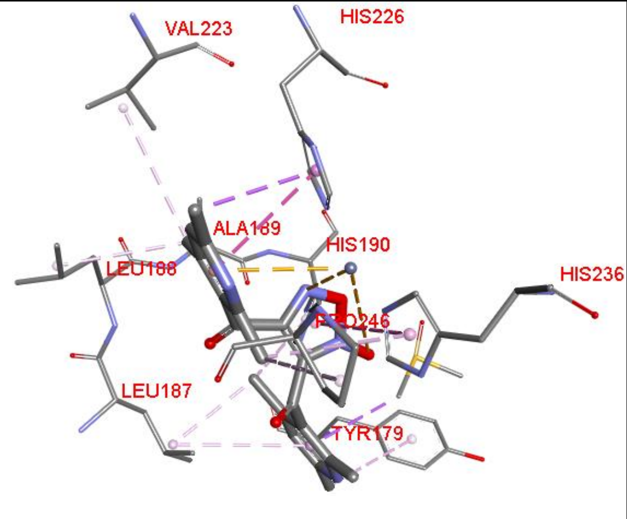

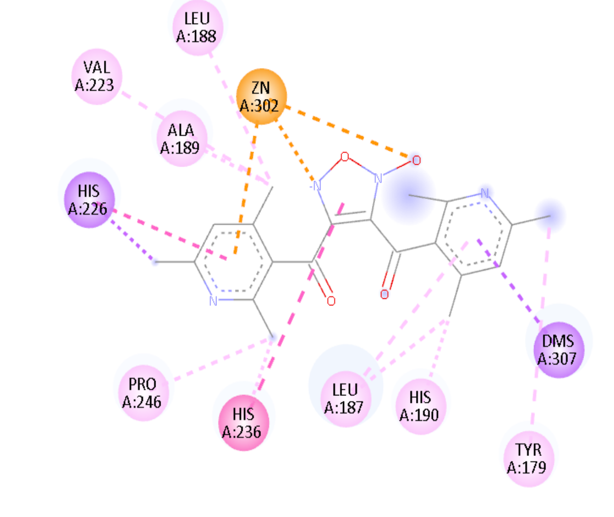

4XCT – VP6

3D-visualizitaion	2D-visualizitaion

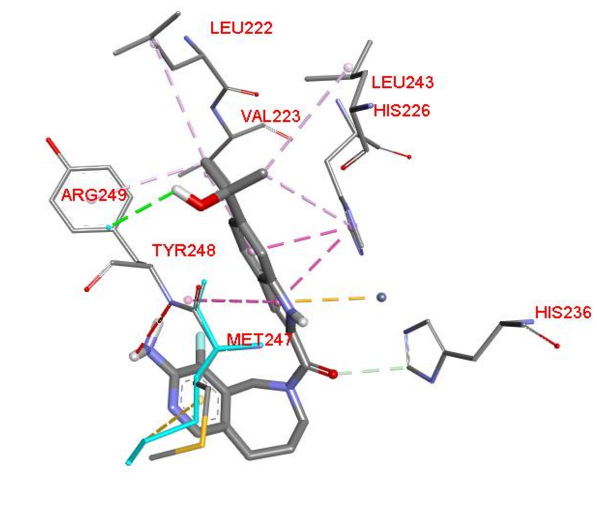

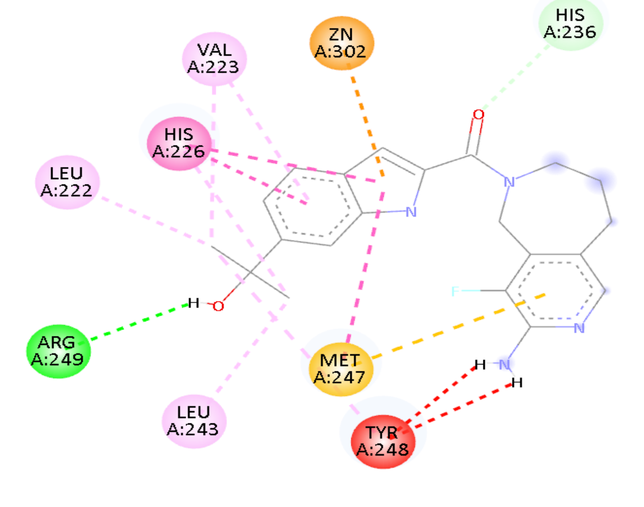

4XCT – B9Z

3D-visualizitaion	2D-visualizitaion

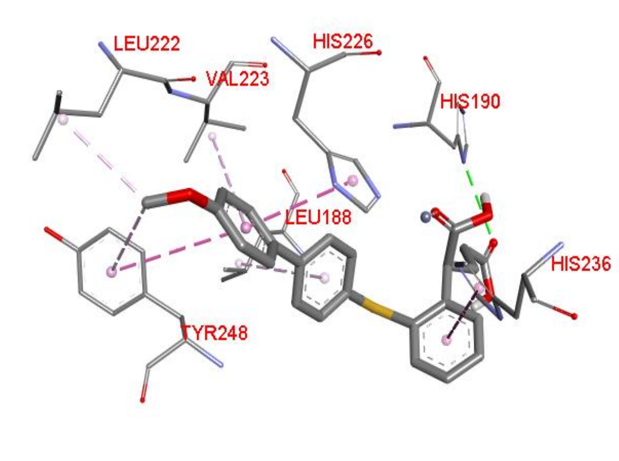

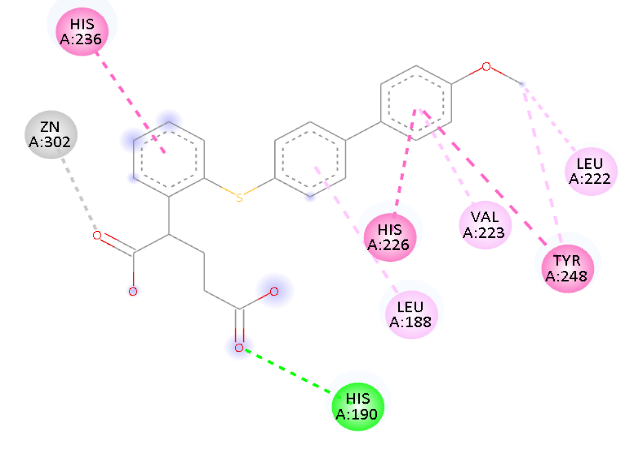

4XCT – N73

3D-visualizitaion	2D-visualizitaion

<table>
<tr>
<td>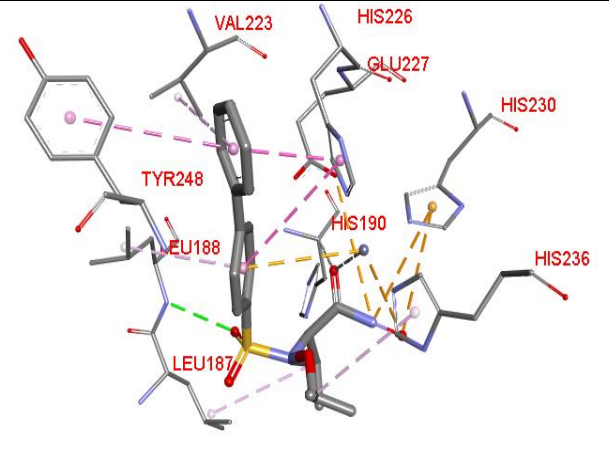
</td>
<td>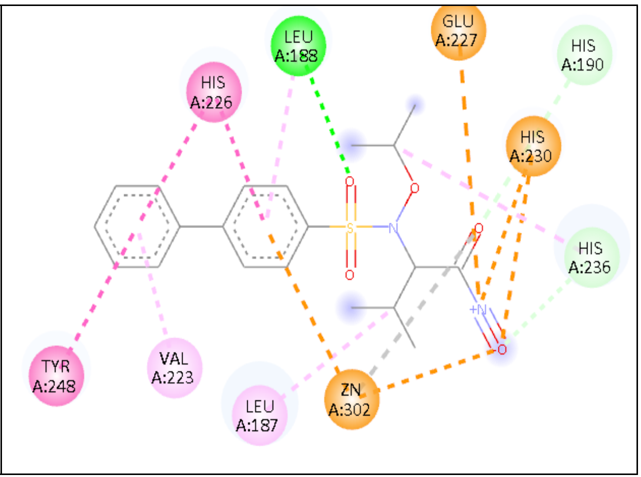
</td>
</tr>
</table>

4XCT – doxycycline

<table>
<tr><th>3D-visualizitaion</th><th>2D-visualizitaion</th></tr>
<tr>
<td>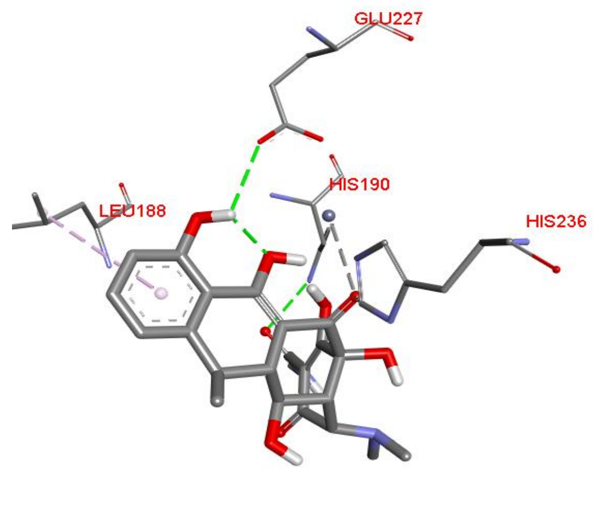
</td>
<td>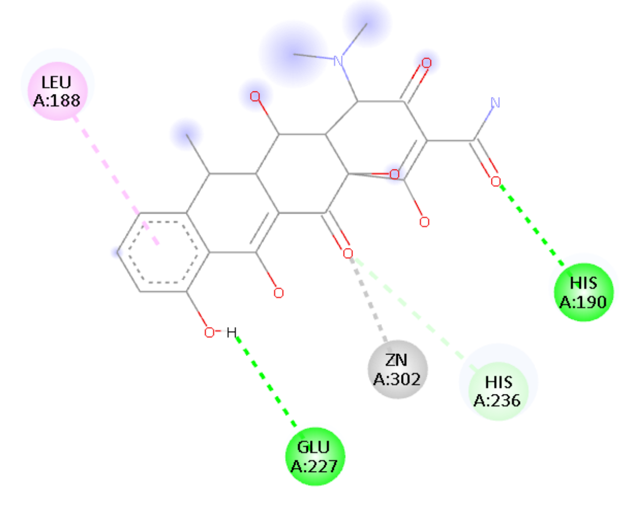
</td>
</tr>
</table>

4XCT – marimastat

<table>
<tr><th>3D-visualizitaion</th><th>2D-visualizitaion</th></tr>
<tr>
<td>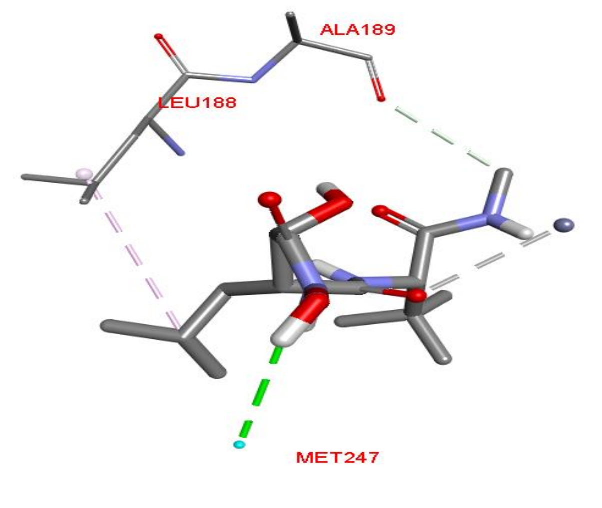
</td>
<td>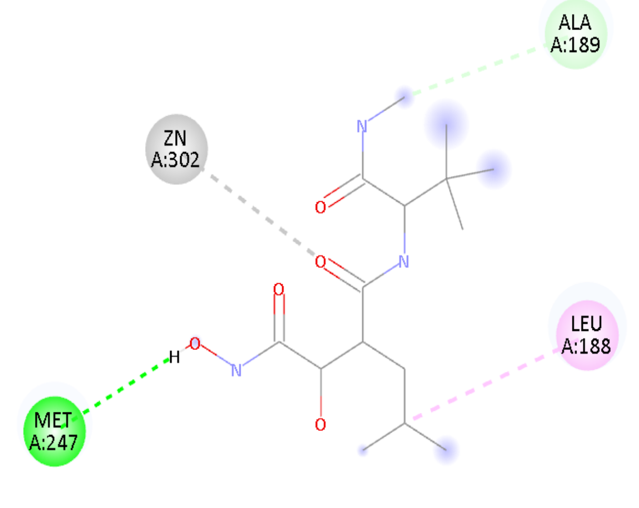
</td>
</tr>
</table>

4XCT – captopril

3D-visualizitaion	2D-visualizitaion
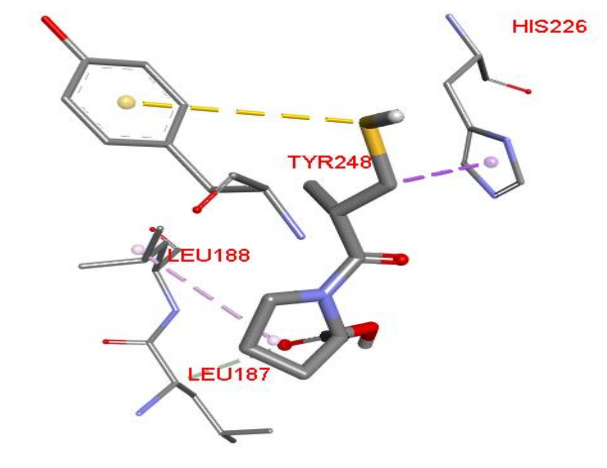	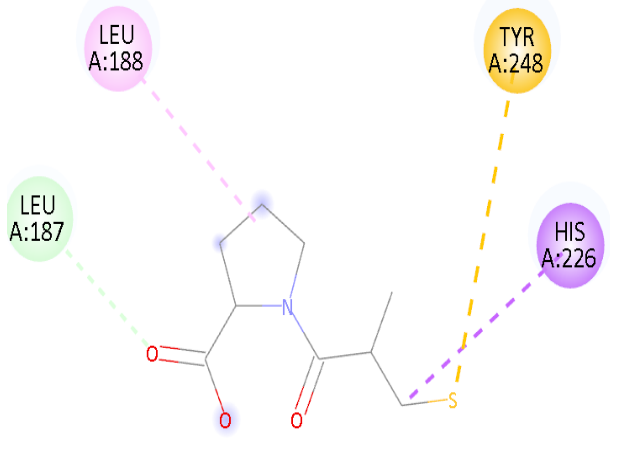
